# Human PNPase causes RNA stabilization and accumulation of R-loops in the *Escherichia coli* model system

**DOI:** 10.1038/s41598-023-38924-x

**Published:** 2023-07-21

**Authors:** Federica A. Falchi, Francesca Forti, Cristina Carnelli, Aurelia Genco, Roberto Pizzoccheri, Caterina Manzari, Giulio Pavesi, Federica Briani

**Affiliations:** 1 Dipartimento di Bioscienze, Università degli Studi di Milano, 20133 Milan, Italy; 2 Dipartimento di Bioscienze, Biotecnologie e Biofarmaceutica, Università degli Studi di Bari “Aldo Moro”, 70121 Bari, Italy

**Keywords:** RNA metabolism, Bacterial genetics, Genetics, Molecular biology

## Abstract

Polyribonucleotide phosphorylase (PNPase) is a phosphorolytic RNA exonuclease highly conserved throughout evolution. In *Escherichia coli*, PNPase controls complex phenotypic traits like biofilm formation and growth at low temperature. In human cells, PNPase is located in mitochondria, where it is implicated in the RNA import from the cytoplasm, the mitochondrial RNA degradation and the processing of R-loops, namely stable RNA–DNA hybrids displacing a DNA strand. In this work, we show that the human PNPase (hPNPase) expressed in *E. coli* causes oxidative stress, SOS response activation and R-loops accumulation. Hundreds of *E. coli* RNAs are stabilized in presence of hPNPase, whereas only few transcripts are destabilized. Moreover, phenotypic traits typical of *E. coli* strains lacking PNPase are strengthened in presence of the human enzyme. We discuss the hypothesis that hPNPase expressed in *E. coli* may bind, but not degrade, the RNA, in agreement with previous in vitro data showing that phosphate concentrations in the range of those found in the bacterial cytoplasm and, more relevant, in the mitochondria, inhibit its activity.

## Introduction

Polyribonucleotide phosphorylase (PNPase) is an evolutionarily conserved phosphorolytic exonuclease that degrades the RNA from 3′- to 5′-end in presence of phosphate (Pi) and Mg^2+^, producing NDPs. In vitro, it also catalyses the reverse template-independent NDP polymerization reaction leading to de novo synthesis of RNA molecules or 3′-end tailing of pre-existing RNAs^1–3^. *Escherichia coli* PNPase (hereafter EcPNPase) in vitro activity on RNA and its cellular function in RNA turnover and in the control of complex phenotypic traits, like biofilm formation and adaptation to the growth at sub-optimal temperature, have been extensively studied by different groups over the many years since bacterial PNPase discovery^1,4,5^. Global mRNA profiling of *E. coli* mutants lacking EcPNPase by RNA-Seq showed that the enzyme controls—directly or indirectly—the level of around 200 transcripts, with the 59% of RNAs increased and the others decreased in EcPNPase absence^6^. In a previous genome-wide analysis of *E. coli* transcription profile performed by microarray, an even higher number of RNAs whose level was modulated by EcPNPase was found (i.e. more than 500)^7^. Auto-regulation of the EcPNPase *pnp* gene is considered a paradigmatic example of post-transcriptional regulation. EcPNPase promotes *pnp* mRNA degradation by digesting a short non-coding RNA that protects it from RNase E-dependent endonucleolytic cleavage^8^. EcPNPase regulates its own expression also via other pathways not requiring the enzyme phosphorolytic activity, namely by binding the *pnp* mRNA and eliciting premature transcription termination and probably, translational repression^9–12^. Far less understood aspects related to EcPNPase are how (and if) small molecules reported to bind and modulate EcPNPase in vitro impact the enzyme activity in the bacterial cells^13–16^, and the EcPNPase role in DNA repair and oxidative stress response. In vitro, the enzyme degrades ssDNA molecules in presence of Pi and Mn^2+^ producing dNDPs. It can also catalyse template-independent polymerization of dNDPs and 3′-end tailing of DNA molecules^17,18^. In vivo, EcPNPase enhances homologous recombination, and *pnp* mutants are sensitive to UV radiation and defective in DNA repair of double-stranded breaks^17,19,20^. The mechanistic function of EcPNPase in DNA repair is unknown. It is still unclear also its role in the protection against oxidative stress, which was imputed to EcPNPase-dependent removal of oxidized RNA, for which such enzyme has high affinity^21^.

The studies on the human orthologous protein (hereafter hPNPase) began much more recently, when the human PNPase (hPNPase) cDNA was cloned and the protein shown to localize in mitochondria^22,23^. hPNPase is essential in mice and human cells, and severe pathological conditions with a wide spectrum of symptoms are caused by mutations in the hPNPase *PNPT1* gene^24–27^. RNA import from cytoplasm into mitochondria and degradation of mitochondrial RNAs are the main activities credited to hPNPase fractions located in the intermembrane space and in the matrix, respectively^28^. Interestingly, also EcPNPase can switch from an RNA degrading to an RNA carrier (and stabilizing) mode depending on the RNA substrate and the interaction with other proteins like the DEAD-box RNA helicase RhlB, which can unfold RNA secondary structures inhibiting EcPNPase activity^6,7,29,30^. Similarly, in the mitochondrial matrix hPNPase forms a complex with the DExH-box RNA helicase SUV3 able to efficiently degrade dsRNA^31–33^. The complex is involved in mitochondrial DNA homeostasis, a function that has been linked to its ability of removing R-loops, i.e. stable RNA–DNA hybrids displacing a DNA strand and potentially interfering with DNA replication^34^. Whether the human enzyme can bind and degrade DNA like its bacterial counterpart is unknown. Conversely, it is known that hPNPase can bind, but not degrade, oxidized RNA^35,36^. hPNPase is 40% identical to EcPNPase (Supplementary Fig. S1), and orthologues of the main EcPNPase interactors, namely RhlB and RNase E^1^, are absent in human cells. Human and *E. coli* PNPases differ as for their optimal Pi concentration for in vitro RNA phosphorolysis, which is around a 100-fold higher for the bacterial than the human enzyme (i.e. 10 vs. 0.1 mM). Indeed, hPNPase phosphorolytic activity was reported to be inhibited in vitro at phosphate concentrations in the range of those found in the *E. coli* cytoplasm^37–40^.

In this work, to get insight into hPNPase activity in a cellular context simpler than that of the human cell and containing high Pi^38^, we analysed the phenotype and global transcription profile of an *E. coli* strain expressing the human enzyme instead of the natural orthologue. We found that hPNPase cannot complement, and actually worsens, several phenotypes due to the lack of EcPNPase. hPNPase deeply impacts *E. coli* gene expression and triggers the SOS response, most likely by eliciting oxidative stress and accumulation of R-loops.

## Results

### Expression in *E. coli* of the *PNPT1*_*Ec*_ gene

**Figure 1:**
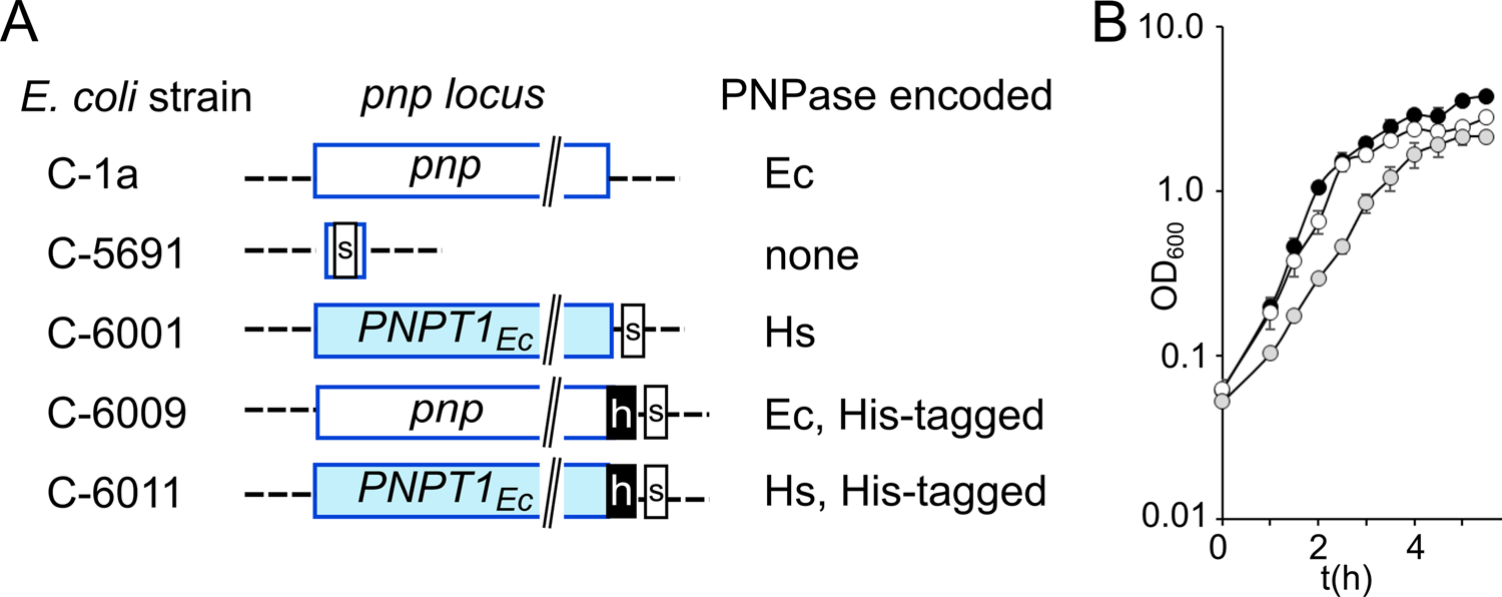
*pnp* locus in recombinant *E. coli* strains and growth of the hPNPase expressing strain*.* (**A**) The structure of the *pnp* locus in the *E. coli* strains listed on the left is shown. Empty blue box, *pnp* ORF; filled blue box, *PNPT1*_*Ec*_ ORF; dashed lines, 5′- and 3′-UTR of the *E. coli pnp* gene; s, FRT scar^41^; h, His-tag. On the right, PNPase variant expressed by each strain. Ec, *E. coli* PNPase; Hs, human PNPase. (**B**) Growth in LD broth. Cultures (N = 3) were grown at 37 °C with shaking in LD measuring the optical density at intervals. The symbols indicated average with standard deviation (StD). C-1a (*pnp*^+^), black symbols; C-5691 (Δ*pnp*), empty symbols; C-6001 (*PNPT1*_*Ec*_), grey symbols.

We constructed by λ RED recombination^41^ an *E. coli* strain expressing human PNPase instead of the natural EcPNPase from the *pnp* chromosomal locus (strain C-6001, carrying the mutation Δ*pnp*::*PNPT1*_*Ec*_; Table 1 and Fig. 1A). The recombinant strain was designed so that transcription of the Δ*pnp*::*PNPT1*_*Ec*_ region (hereafter *PNPT1*_*Ec*_) from the *pnp* promoter produced an mRNA with the same 5′-UTR as the *pnp* transcript and the hPNPase open reading frame (ORF) with the *E. coli* codon usage^15^.

**Table 1:** Bacterial strains and plasmids.

Name	Relevant features	References
Bacterial strains		
C-1a	Prototroph	^42^
C-5691	C-1a Δ*pnp*-751	^43^
C-5692	C-5691 (DE3)	^13^
C-6001	C-1a Δ*pnp::PNPT1*_*Ec*_	This work
C-6009	C-1a *pnp-*His tag	This work
C-6011	C-1a Δ*pnp::PNPT1*_*Ec*_*-*His tag	This work
Plasmids		
pCP20	Flippase encoding plasmid	^41^
pKD4	kanR encoding plasmid	^41^
pKD46	λRED plasmid	^41^
pET28b-H6-hPNPase	hPNPase coding sequence with the *E. coli* codon usage (*PNPT1*_*Ec*_) and N-ter 6 × His tag	^14^
pETD-hPNP	Carries IPTG-inducible *PNPT1*_*Ec*_ cloned in pETDuet-1 (Novagen)	This work

We found that *PNPT1*_*Ec*_ cultures had longer generation time (g) in exponential phase and reached lower optical density after 24 h than both *pnp*^+^ and Δ*pnp* cultures (Table 2 and Fig. 1B), showing that the presence of hPNPase affected growth more than the simple lack of EcPNPase.

**Table 2:** Growth of the *PNPT1*_*Ec*_ strain.

	*pnp*^+^	Δ*pnp*	*PNPT1*_*Ec*_
g (min)^a^	24.7 ± 1.3	32.6 ± 1.7	39.7 ± 3.4
OD_600_^a,b^	5.9 ± 0.2	4.0 ± 0.3	2.8 ± 0.2

^a^Cultures of C-1a (*pnp*^+^), C-5691 (Δ*pnp*), and C-6001 (Δ*pnp*::*PNPT1*_*Ec*_) grown with aeration at 37 °C in LD broth. Mean (N = 3) ± standard deviation is reported.

^b^OD_600_ reached by the culture after 24 h. All cultures were in stationary phase.

**Figure 2:**
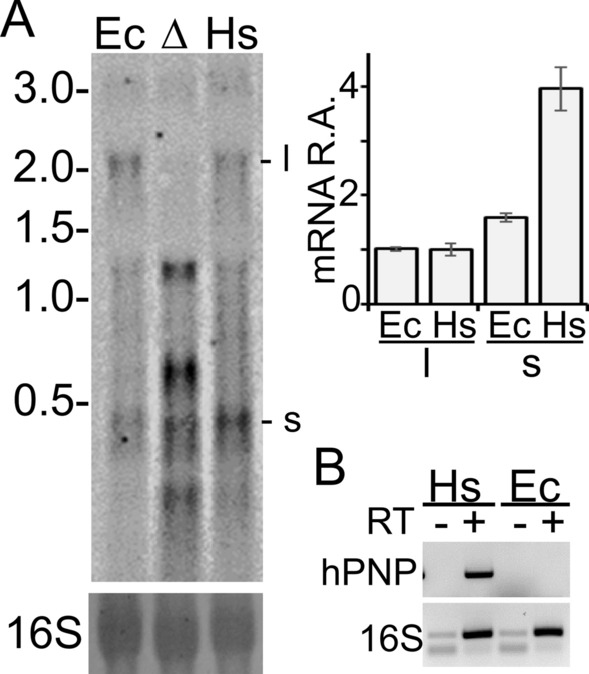
*PNPT1*_*Ec*_ gene transcription pattern. (**A**) Northern blotting. RNA was extracted from exponential cultures of C-1a (*pnp*^+^; Ec), C-5691 (Δ*pnp*; Δ) and C-6001 (*PNPT1*_*Ec*_; Hs). 20 µg of RNA were run on 1.5% denaturing agarose gel, blotted onto a nylon filter and hybridized with the RNA pnp-5′ radiolabelled riboprobe as detailed in Methods. Upper left panel, MW marker length and position in the gel are reported on the left in kb, whereas the main signals corresponding to 2.2 kb (l) and < 0.5 kb (s) transcripts are indicated on the right; lower left panel, 16S rRNA stained with methylene blue before hybridization as loading control; right panel, mRNA Relative Amount (R.A.). l and s signals were quantified with ImageQuant and values normalized by l value in *E. coli*. Bars represent average (N = 3) with standard deviation (StD). (**B**) RT-PCR analysis. 1 µg of RNA extracted from exponential cultures of C-1a (Ec) and C-6001 (Hs) grown as described above was incubated with random hexamers and with (+) or without (−) reverse transcriptase. The cDNA and control samples were PCR-amplified with primers specific for either the *PNPT1*_*Ec*_ mRNA (hPNP) or 16S rRNA (16S) and run on a 2% agarose gel.

**Figure 3:**
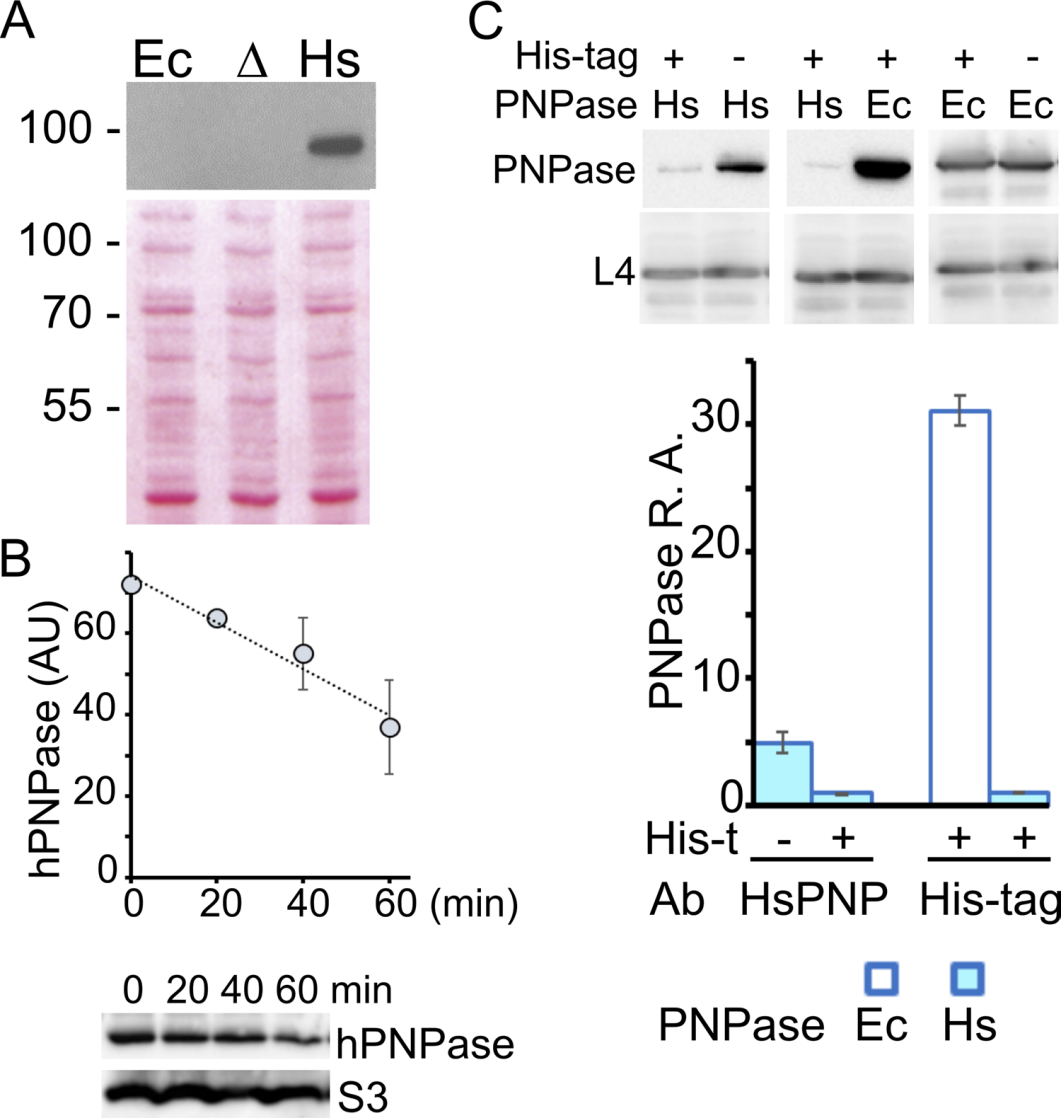
Abundance and stability of hPNPase in the *PNPT1*_*Ec*_ strain. (**A**) Western blotting of proteins extracted from exponential cultures of C-1a (*pnp*^+^; Ec), C-5691 (Δ*pnp*; Δ) and C-6001 (*PNPT1*_*Ec*_; Hs). The proteins (10 µg) were run on 10% polyacrylamide-SDS gel and blotted onto a nitrocellulose membrane. The filter was stained with Ponceau S (lower panel) to check loading and hybridized with hPNPase-specific monoclonal antibodies (upper panel). The position of MW markers is reported on the left in kDa. (**B**) hPNPase stability. 15 µl of *PNPT1*_*Ec*_ strain protein extracts (0.375 OD_600_) were loaded onto 10% polyacrylamide gel, blotted onto a nitrocellulose membrane and hybridized with polyclonal anti-hPNPase and anti-S3 antibodies. The results of a typical experiment are shown under the graph. To plot the decay curve, hPNPase signals were quantified with ImageLab (Bio-Rad), using S3 signals as loading control. Symbols in the plot represent mean with StD (N = 3). AU, arbitrary units. (**C**) Upper part. Western blotting of proteins extracted from exponential cultures of C-6009 (+, Hs); C-6001 (−, Hs), C-6011 (+, Ec) and C-1a (−, Ec) grown at 37 °C in LD up to OD_600_ = 0.4. The proteins were run on a 12% polyacrylamide- SDS gel, blotted onto a nitrocellulose membrane and hybridized with hPNPase—(upper left panel), His tag—(upper central panel), EcPNPase—(upper right panel) or L4—specific (lower panels, loading control) antibodies. Lower part, PNPase Relative Amount (R. A.). The bars represent average with StD (N = 3) of quantification of PNPase signals with Image Lab (Bio-Rad).

*PNPT1*_*Ec*_ gene expression analysis by Northern blotting and RT-PCR showed that the gene was transcribed during exponential growth at 37 °C (Fig. 2A,B). The main transcripts were a ca. 2.2 kb long mRNA, potentially covering the whole *PNPT1*_*Ec*_ gene, and a shorter RNA, less than 0.5 kb long. Overall, the *PNPT1*_*Ec*_ transcription profile was similar to that of the *pnp* locus in the *pnp*^+^ strain, although the short RNA was more abundant in *PNPT1*_*Ec*_ than in the *pnp*^+^ (Fig. 2A). Western blotting with a hPNPase-specific antibody showed a single signal in *PNPT1*_*Ec*_ corresponding to a protein migrating between the 70 and the 100 kDa molecular weight (MW) markers, compatible with the predicted MW of hPNPase lacking the mitochondrial localization signal (i.e. 81 kDa; Fig. 3A). The half-life of the protein at 37 °C was 1.3 ± 0.5 h (Fig. 3B), indicating that like EcPNPase^44^, the human protein is relatively stable in *E. coli* exponential cultures.

To compare the level of hPNPase in the *PNPT1*_*Ec*_ strain with that of EcPNPase in the *pnp*^+^, we constructed two strains expressing histidine-tagged variants of either EcPNPase or hPNPase (C-6009 and C-6011, respectively; Fig. 1A) from the *pnp* locus. As shown in Fig. 3C, the expression of His-tagged EcPNPase was around 30-fold higher than that of His-tagged hPNPase. However, the untagged hPNPase in *PNPT1*_*Ec*_ was five-fold more abundant than its His-tagged version, whereas the tagged and untagged variants of EcPNPase were equally expressed (Fig. 3C). Based on these data, we evaluated that in mid-exponential cultures at 37 °C, hPNPase should be around sixfold less abundant in the *PNPT1*_*Ec*_ than EcPNPase in the *pnp*^+^ strain. Since the amount of EcPNPase has been estimated to be *ca*. 5700 molecules/cell^45^, hPNPase should be at 900–1000 molecules/cell, more abundant than the median *E. coli* protein (i. e. 526 molecules/cell)^45^. We did not investigate the mechanism determining low abundance of the tagged hPNPase, which could be due to decreased mRNA or protein stability.

### Phenotypes due to the lack of *E. coli* PNPase are strengthened in presence of hPNPase

**Figure 4:**
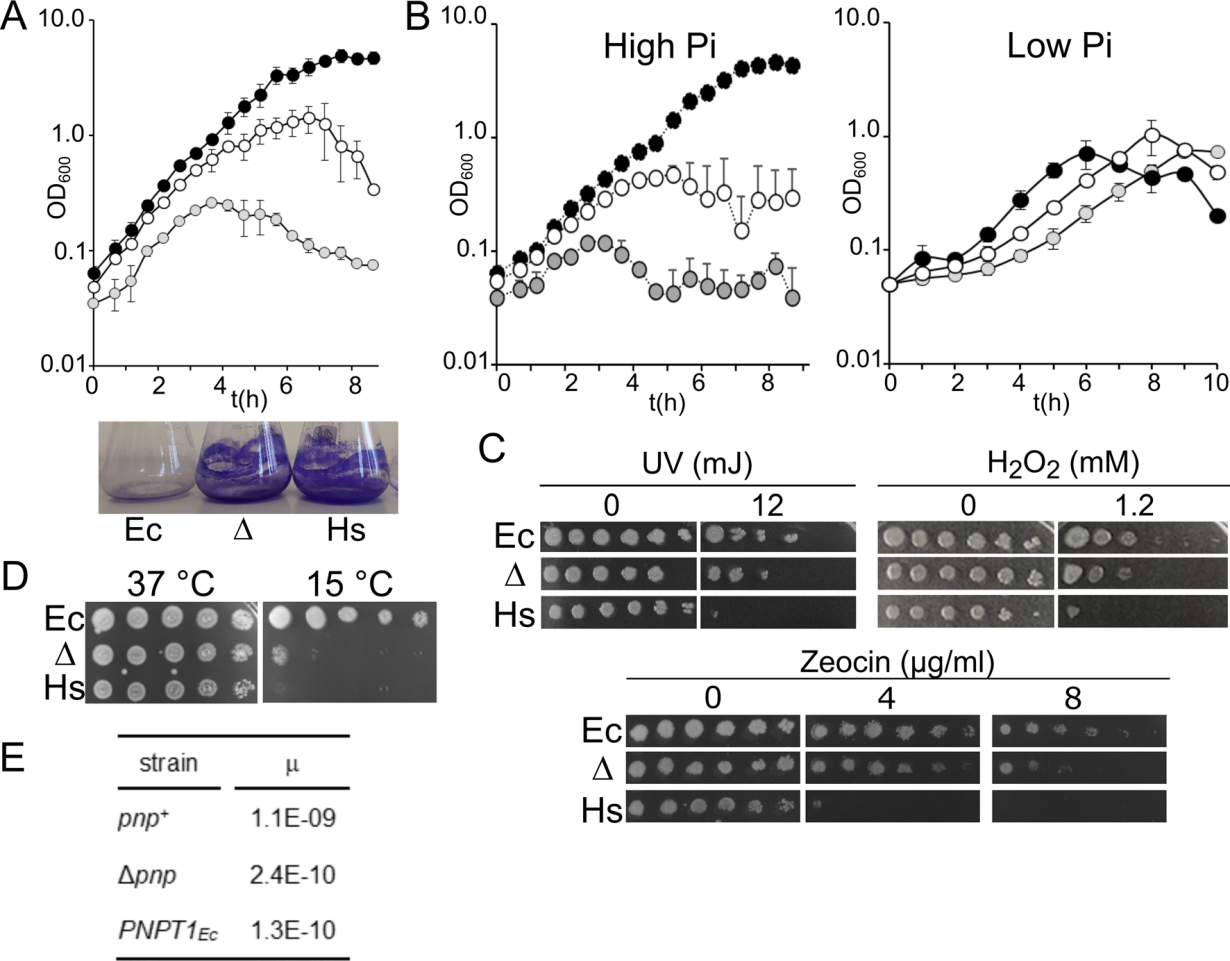
hPNPase effect on EcPNPase-dependent phenotypes. (**A**, **B**) Growth in minimal media of C-1a (*pnp*^+^), black symbols; C-5691 (Δ*pnp*), empty symbols; C-6001 (*PNPT1*_*Ec*_), grey symbols. (**A**) Cultures (N = 3) were grown at 37 °C with shaking in M9/supp measuring the optical density at intervals. The symbols indicated average with StD. After overnight incubation, cultures grown in M9 were discarded and the flasks stained with crystal violet to colour adherent cells (lower panel). Ec, C-1a (*pnp*^+^); Δ, C-5691 (Δ*pnp*); Hs, C-6001 (*PNPT1*_*Ec*_). (**B**) Cultures were grown at 37 °C with shaking in M9 (High Pi) or M9/2.5 (Low Pi) with 0.4% glucose measuring the optical density at intervals. The symbols indicated average with range (N = 2, High Pi) or with StD (N = 3, Low Pi). (**C**) Serial dilutions of overnight cultures were plated on LD-agar and incubated overnight at 37 °C in presence or absence of zeocin or H_2_O_2_. To test UV sensitivity, the plates were exposed to the indicated dose of UV rays (λ = 254 nm) in a Stratalinker 2400 (Stratagene) before incubation. Strains are indicated as in (**A**). (**D**) Serial dilutions of overnight cultures were plated on LD-agar and incubated overnight at 37 °C or 7 days at 15 °C. Strains are indicated as in A. (**E**) Streptomycin resistance mutation rate (µ) determined by fluctuation test on 12 independent cultures per strain.

We tested whether hPNPase could compensate the lack of EcPNPase and complement phenotypes caused by *pnp* null mutations in *E. coli*. In particular, we assayed (i) aggregation of cultures growing in minimal medium with glucose as sole carbon source, detectable as a drop in the optical density of late exponential cultures^46^. This phenotype was tested also at low Pi concentration, which should increase and decrease, respectively, hPNPase and EcPNPase phosphorolytic activity^28^; (ii) grow inhibition at temperatures below 18–20°C^11,47^; (iii) increased sensitivity to H_2_O_2_, causing oxidative damage, and to mutagenic agents like UV rays and zeocin, an antibiotic inducing DNA double-strand breaks (DSBs)^17,19–21^; and iv) reduced frequency of spontaneous mutations^19,20^. As shown in Fig. 4A, the turbidity of *PNPT1*_*Ec*_ cultures growing in minimal medium started decreasing when the cultures were at OD_600_ = 0.2–0.3, whereas the Δ*pnp* strain aggregated at higher cell density. Interestingly, we observed that low Pi concentration promoted the aggregation of the *pnp*^+^ strain, whereas it prevented *PNPT1*_*Ec*_ aggregation and had a modest effect, if any, on the Δ*pnp* strain (Fig. 4B).

The *PNPT1*_*Ec*_ strain was more sensitive to H_2_O_2,_ UV rays and zeocin than the Δ*pnp* (Fig. 4C) and cold sensitive like the Δ*pnp* (Fig. 4D). Moreover, its spontaneous mutation frequency was even lower than that of the Δ*pnp* (Fig. 4E). Cold sensitivity and increased adhesion and aggregation were exhibited also by a Δ*pnp* mutant overexpressing hPNPase from a multicopy plasmid (Supplemental Fig. S2), implying that lack of compensation of these Δ*pnp-*linked phenotypes by hPNPase was not due to the lower expression of the *PNPT1*_*Ec*_ gene with respect to *pnp*.

### hPNPase deeply affects the *E. coli* transcriptional profile

**Figure 5:**
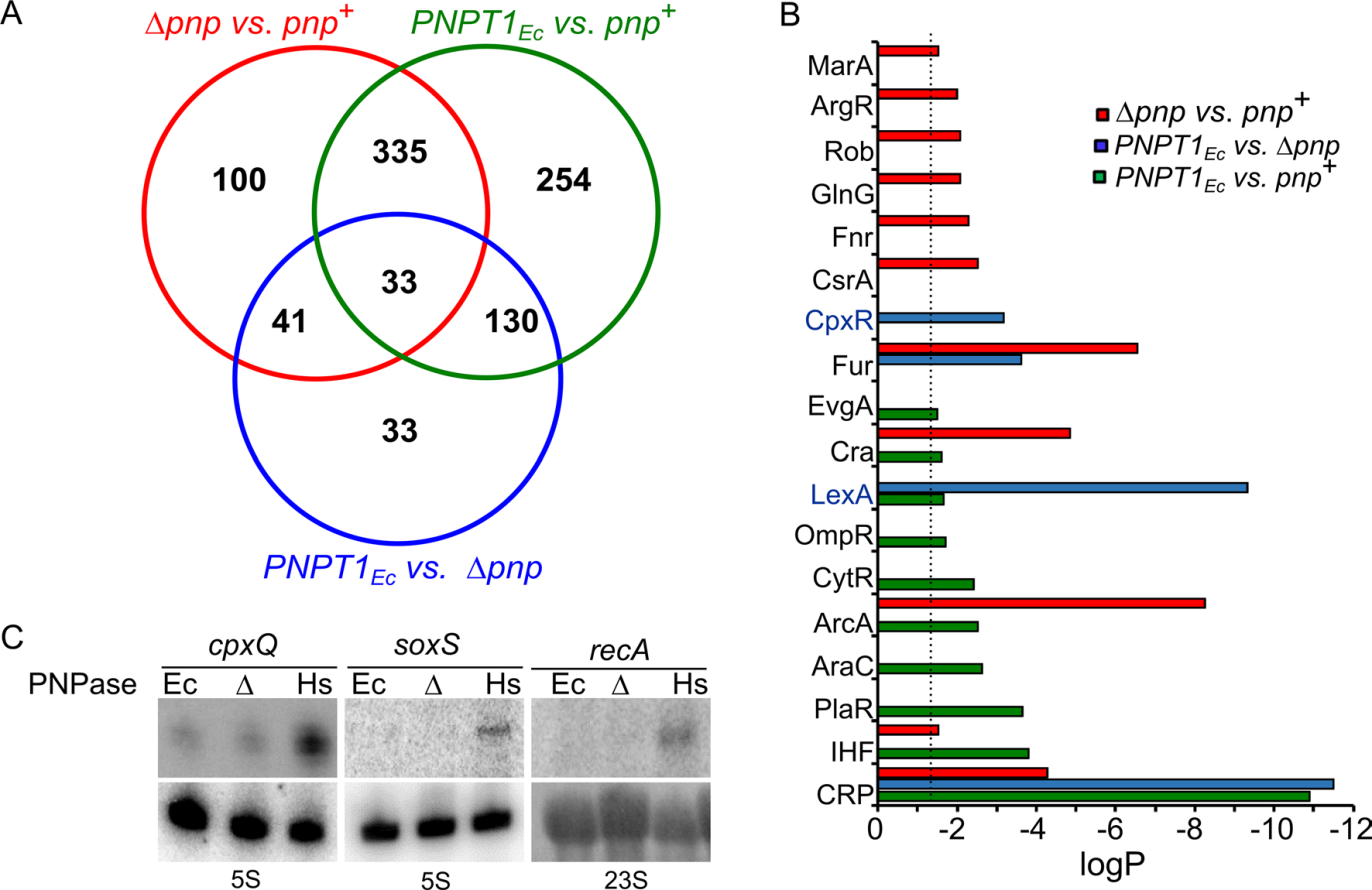
Transcriptome analysis and differentially expressed regulons. (**A**) Venn-diagrams of differentially expressed genes (DEGs). Venn diagrams representing the number of DEGs in the indicated pairwise comparisons. *pnp*^+^, C-1a; Δ*pnp*, C-5691; *PNPT1*_*Ec*_, C-6001. (**B**) Differentially expressed regulons (*P* < 0.5; dotted line) in pairwise comparison. Enrichment analysis was performed with the Pathway tool available at the EcoCyc website^51^. Colour code and strain tags are as in (**A**). (**C**) Northern blotting analysis of gene expression. RNA was extracted from exponential cultures of C-1a (Ec), C-5691 (Δ) and C-6001 (Hs). 20 and 10 µg of RNA were run on 1.5% denaturing agarose gel (*recA* panel) or 6% denaturing polyacrylamide gel (*cpxQ* and *soxS* panels), respectively, blotted onto nylon filters and hybridized with radiolabelled oligonucleotides complementary to the mRNAs under analysis. 5S and 23S rRNA signals were used as loading controls.

To assess the impact of hPNPase on *E. coli* physiology, we analysed by RNA-Seq the global transcription profile of the *PNPT1*_*Ec*_ strain in comparison with that of the *pnp*^+^ and the Δ*pnp*. RNA was extracted from exponential cultures grown in broth at 37 °C and the transcriptomes of the three strains were compared to identify differentially expressed genes (DEGs) with FDR < 0.01 and |log_2_(FoldChange)|≥ 1 as thresholds (Fig. 5A; Supplemental Table S1). DEGs found in each pairwise comparison are listed in Supplemental Table S1. 307 and 202 genes were down- and up-regulated in the Δ*pnp* mutant versus the *pnp*^+^. These genes are directly or indirectly regulated by PNPase. 421 and 331 genes were down- or up-regulated, respectively, in *PNPT1*_*Ec*_ versus *pnp*^+^, showing that replacing the natural *pnp* gene with the human ortholog alters the transcriptional profile more than deleting the bacterial gene. Accordingly, 237 genes had different expression in the Δ*pnp* with respect to the *PNPT1*_*Ec*_ strain, with 135 and 102 down- and up-regulated genes, respectively, in *PNPT1*_*Ec*_. The genes with the largest decrease in expression levels in presence of hPNPase (log_2_(FoldChange) < − 3) were genes belonging to *tna* and *tdc* operons controlling tryptophan and threonine degradation, and the *glpABC* genes encoding the anaerobic glycerol-3-phosphate dehydrogenase^48,49^. Most of the genes with highest relative expression in *PNPT1*_*Ec*_ (log_2_(FoldChange) > 3), like *tisB, umuD, sulA, dinI, recN* and *recA*, belonged to the SOS response, a regulatory mechanism that allows the coordinated induction of tens of genes, mainly involved in DNA repair, in presence of DNA damage^50^.

To better understand how hPNPase impacts the bacterial cell physiology, we looked for regulons moving in the pairwise comparisons between the *PNPT1*_*Ec*_ and the Δ*pnp* strains and maintaining similar expression in presence or absence of the EcPNPase (i.e. in the *pnp*^+^ vs. Δ*pnp* comparison) (Fig. 5B). These regulons should respond to the human, and not to the bacterial, enzyme. We found that the LexA and CpxR regulons were activated in presence of the human enzyme, as confirmed also by Northern blotting showing enhanced expression of *recA* and *cpxQ* genes in *PNPT1*_*Ec*_ (Fig. 5B,C). LexA is the repressor of the SOS response^50^, whereas CpxR is the regulator of the Cpx pathway mediating adaptation to envelope stress at the inner membrane^52^.

Overall, these data indicate that hPNPase has differential activity with respect to the bacterial enzyme and that may cause genotoxic and membrane stress.

### hPNPase causes SOS response induction

The SOS response is induced by LexA repressor self-cleavage stimulated by activated RecA (RecA*), which is a RecA filament assembled on a ssDNA scaffold^50^. Constitutive induction of the SOS response in *PNPT1*_*Ec*_ indicates that this strain may have high levels of ssDNA, which typically accumulates when cells attempt to replicate damaged DNA.

**Figure 6:**
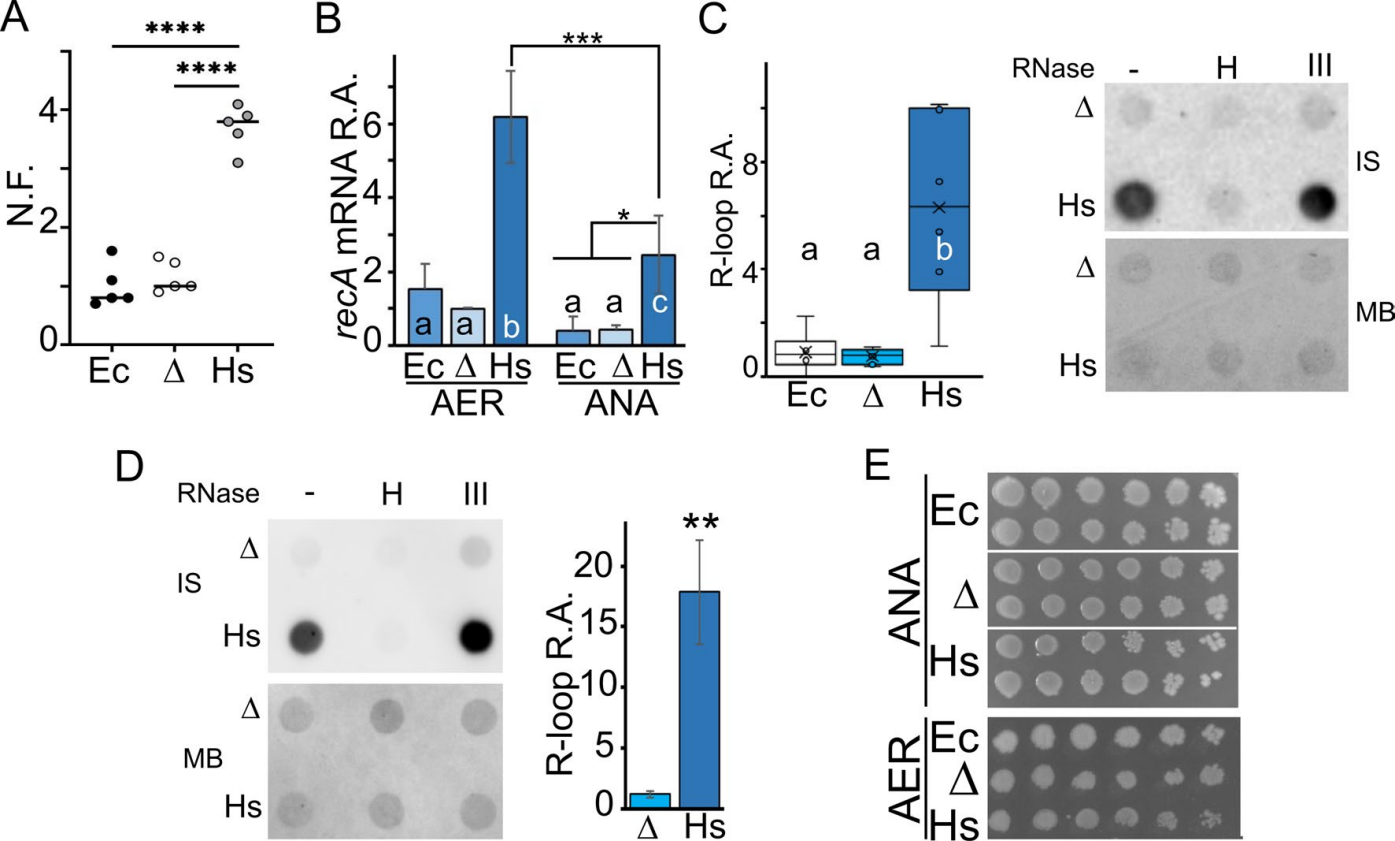
recA induction and ROS and R-loop accumulation in *PNPT1*_*Ec*_. (**A**) ROS accumulation. Normalized fluorescence (N. F.) of C-1a (*pnp*^+^, Ec); C-5691 (Δ*pnp*, Δ); C-6001 (*PNPT1*_*Ec*_, Hs) cultures (N = 5) in presence of DCFH-DA. Significance was evaluated with ANOVA and Tukey post hoc analysis. ****, *P* < 0.0001.  (**B**) RT-qPCR analysis of *recA* expression. RNA was extracted from replicate cultures (N = 3) grown in aerobiosis (AER) or anaerobiosis (ANA). ΔCt between the gene of interest and the 16S gene was arbitrarily set at 1 for one of the samples extracted from C-1a AER (reference condition). The bars represent relative amount (R.A.) with respect to the reference condition and show the average with standard deviations of the values obtained on three biological replicates, each performed in duplicate. Bars sharing the same letter represent averages not significantly different from each other according to ANOVA and Tukey post hoc comparisons. ***, *P* ≤ 0.001; *, *P* < 0.05. (**C**, **D**) Dot blot quantification of R-loops. Strains are indicated as in A. DNA extracted from cultures grown at high (**C**) or low (**D**) oxygen were analysed. C, left panel. Dot blot signals obtained by immunostaining of genomic DNA with the S9.6 antibody were quantified with ImageQuant. The median (N = 6) is reported inside the boxes (line). The whiskers represent the minimum and maximum values observed. Boxes sharing the same letter are not significantly different from each other according to ANOVA and Tukey posthoc comparisons. (**C**) Right panel, and (**D**) left panel. Genomic DNA samples were dot blotted after incubation with RNase H or III, as described in Methods. –, mock incubation without RNase addition. Immunostaining (IS) with S9.6 antibody. MB, staining of the filter with methylene blue as loading control. (**D**) Right panel. Dot blot signals obtained by immunostaining of genomic DNA with the S9.6 antibody were quantified with ImageQuant. Bars represent average (N = 3) with standard deviation. Significance of the difference was estimated with two-tail t-test. **, *P* < 0.01. (**E**) Serial dilution of C-1a (Ec), C-5691 (Δ) and C-6001 (Hs) grown on LD-agar at 37 °C in aerobiosis (AER) or anaerobiosis (ANA). Duplicate cultures are shown in the ANA panel.

The SOS response can be induced in *E. coli* by Reactive oxygen species (ROS), which are the major source of endogenous DNA damage^53,54^. Considering that hPNPase induces ROS when overexpressed in human cells^55^, we speculated that hPNPase may either induce ROS or interfere with ROS detoxification. In fact, ROS levels measured using the fluorescent probe DCFH-DA resulted significantly higher in *PNPT1*_*Ec*_ than in *pnp*^+^ (Fig. 6A), and the *soxS* gene, a transcriptional activator of the oxidative stress genes^54^, was induced in presence of hPNPase (Fig. 5C; Supplemental Fig. S3A,B). To check whether oxidative damage could be the cause of constitutive SOS induction, we tested *recA* gene expression, taken as a measure of SOS response induction, in anaerobic cultures. In these conditions, the extent of oxidative damage should be reduced^54^. As shown in Fig. 6B, *recA* expression was diminished in anaerobiosis, although it remained higher in the *PNPT1*_*Ec*_ than in the other strains. Thus, oxidative damage may contribute to *recA* induction and SOS response activation but it is not the only cause of this phenomenon.

### hPNPase causes the accumulation of R-loops

hPNPase was reported to participate in R-loop removal from mitochondrial genome together with the SUV3 RNA helicase^34^. Since the SOS response is induced in mutants lacking RNase HI, which processes R-loops, and ssDNA, which is the SOS response inducing stimulus^50^, is exposed in R-loops^56,57^, we tested whether the R-loop level was enhanced in presence of hPNPase. Dot blot analysis of genomic DNA with the S9.6 antibody, which primarily recognizes DNA-RNA hybrids though having some affinity also for dsRNA^58^, showed sixfold stronger signals with the *PNPT1*_*Ec*_ DNA than with the DNA of the other *pnp*^+^ and Δ*pnp* strains (Fig. 6C). Digestion of genomic DNA with the DNA-RNA hybrid-specific nuclease RNase H before the dot blot analysis eliminated the difference in signal intensity among the genomic DNAs extracted from the three strains, difference which was instead maintained after DNA treatment with the dsRNA-specific RNase III (Fig. 6C). Thus, hPNPase stimulates R-loops in *E. coli*.

In mammals, DNA damage and replication slowing induced by ROS causes R-loop formation^59–61^. To assess whether oxidative damage could be involved in hPNPase-dependent R-loop accumulation, we measured R-loops in bacteria grown anaerobically. We found that also in these condidtions, the amount of R-loops was strongly increased in presence of hPNPase (Fig. 6D), making unlikely that R-loops accumulate in presence of hPNPase only as a consequence of oxidative damage.

Another mechanism that could cause R-loop increase may be low expression of the R-loop processing enzyme RNase HI in presence of hPNPase. However, the *rnhA* gene, encoding RNase HI, was not differentially expressed in the *PNPT1*_*Ec*_ (Supplemental Table S1). Consistent with it, the *PNPT1*_*Ec*_ growth was not impaired in anaerobiosis (Fig. 6E), a condition in which *rnhA* mutants cannot grow^57^.

### hPNPase is essentially devoid of RNA degradation activity in *E. coli*

To assess whether the human enzyme had catalytic activity in the bacterial context, we measured the overall stability of *E. coli* RNAs in presence or absence of the human enzyme. This was done by analysing by RNA-Seq transcript levels (measured in counts per million, CPM) of the *PNPT1*_*Ec*_, *pnp*^+^ and Δ*pnp* strains immediately before (T0) and 4 min after blocking transcription initiation with rifampicin (T4). The residual RNA amounts of the different *E. coli* RNA species at T4 should depend on the stability of each of them in the three strains. Stability was thus estimated for each gene according to the percentage of its RNA found remaining at T4, expressed as the percentage of the CPM values at T0 maintained at T4 (Supplementary Fig. S3C). For simplicity, genes were classified in four categories from stable RNAs to unstable RNAs, as follows:

1. RNA remaining > 75%;
2. 50% < RNA remaining < 75%;
3. 25% < RNA remaining < 50%;
4. RNA remaining < 25%.

**Figure 7:**
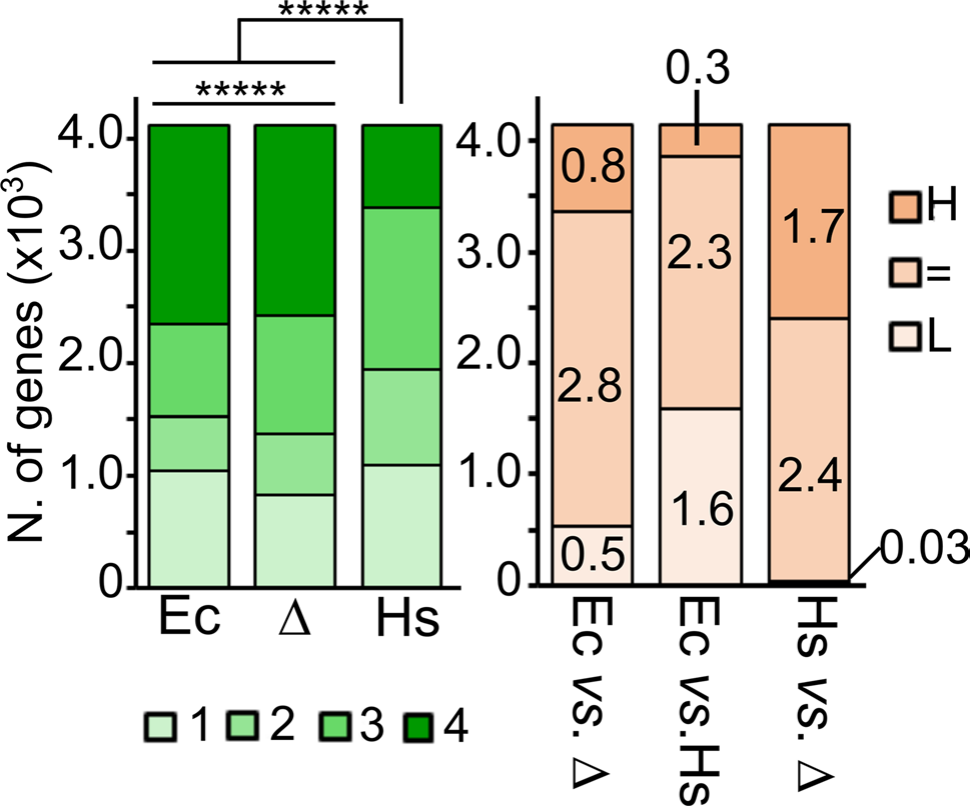
Stability of *E. coli* mRNA in presence of hPNPase. Left panel. mRNAs extracted from exponential cultures at 37 °C of C-1a (*pnp*^+^*;* Ec), C-5691 (Δ*pnp*; Δ) and C-6001 (*PNPT1*_*Ec*_; Hs) were divided in four categories with decreasing stability (from 1, most stable, to 4, least stable). Significance of difference of the distribution was estimated with Pearson’s Chi-Square test. *****, *P* < 0.00001. Right panel. Distribution of mRNAs with lower (L), equal (=) or higher (H) stability in (i) C-1a (*pnp*^+^*;* Ec) than in C-5691 (Δ*pnp*; Δ), Ec versus Δ; (ii) C-1a (*pnp*^+^*;* Ec) than in C-6001 (*PNPT1*_*Ec*_; Hs), Ec versus Hs; and (iii) in C-6001 (*PNPT1*_*Ec*_; Hs) than in C-5691 (Δ*pnp*; Δ), Hs versus Δ. The figures on column sectors indicate thousands of genes belonging to each category.

As shown in Fig. 7 and Supplemental Table S2, hPNPase caused a drastic redistribution of the mRNA levels among the categories, with a strong decrease of the most unstable mRNAs (i.e. those in cat. 4). In particular, only 27 RNAs where less stable in presence of hPNPase than in Δ*pnp*, whereas 521 RNAs were less stable in presence of EcPNPase than in Δ*pnp* (Fig. 7, right panel, *pnp*^+^ vs. Δ*pnp*). Conversely, the transcripts more stable in the presence of hPNPase were more than double those stabilized in the presence of EcPNPase (1738 and 786 in *PNPT1*_*Ec*_ and *pnp*^+^, respectively, in comparison with Δ*pnp*). These data are consistent with a very limited, if any, RNA degrading activity of hPNPase in *E. coli* cells.

## Discussion

In this work, we characterized an *E. coli* strain that carries the *PNPT1*_*Ec*_ gene encoding the human PNPase inserted into the *pnp* locus. Our data clearly show that human PNPase cannot compensate the phenotypic effects due to the lack of the natural EcPNPase. On the contrary, the PNPase of spinach chloroplast complemented the cold sensitive phenotype of an *E. coli pnp* mutant^62^. This difference may be due to higher similarity of chloroplast PNPase to EcPNPase, as both the RPH2 and the KH-S1 domains of EcPNPase are more similar to those of spinach PNPase than to hPNPase ones. Indeed, only spinach PNPase, and not hPNPase, can form heterotrimers with EcPNPase subunits^37,62^. Since residues 327–331 of EcPNPase, with which RNase E forms hydrogen-bonding interactions^63^, are not conserved in the human enzyme, it is very unlikely that hPNPase could be recruited in the RNase E-based RNA degradosome. Thus, it seems sound that hPNPase cannot replace the endogenous enzyme in all those activities in which EcPNPase participates as a component of the multisubunit RNA degradosome.

The comparison of total RNA stability between the Δ*pnp* and the *PNPT1*_*Ec*_ strains showed that the bacterial enzyme caused the destabilization of more than 500 transcripts, whereas only 27 were modestly destabilized in *PNPT1*_*Ec*_, suggesting that the human enzyme may be substantially devoid of degradative activity in the bacterial context. Previous in vitro analyses showed that the phosphorolytic activity of hPNPase is strongly impaired when Pi concentration is greater than 2.5 mM^37^. Since in the cytoplasm of *E. coli* growing cells Pi concentration is around 10 mM^38^, this would explain why hPNPase has no degradation activity in the bacterial cell. Consistent with this hypothesis, at low Pi, which should improve hPNPase phosphorolytic activity and worsen that of EcPNPase, the strong aggregative phenotype shown in M9 by *PNPT1*_*Ec*_ is suppressed, whereas the *pnp*^+^ strain aggregates. Although these data should be taken with some caution, giving the pleiotropic effects that Pi concentration modulation may have, they support the hypothesis that hPNPase may be inactive in *E. coli* because of high Pi level. Interestingly, Pi should be at about 10 mM in the mammalian cell cytoplasm^64,65^ and, as a consequence, also in the mitochondrial intermembrane space, as this compartment has similar composition to the cytosol with respect to ions and small molecules to which the mitochondrial outer membrane is highly permeable^66,67^. Indeed, phosphate concentration in whole mitochondria has been estimated to be greater than 2.5 mM^64,68,69^, implying that hPNPase phosphorolytic activity should be essentially inhibited or limited to sub-mitochondrial regions in which Pi concentration is locally and/or temporarily low. In agreement with this hypothesis, mitochondrial RNA (mtRNA) degradation by the hPNPase-hSUV3 complex, namely the mitochondrial degradosome, has been reported to take place in relatively few spots inside the mitochondrial matrix^32^.

Lack of catalytic activity and impaired interaction with other components of the bacterial RNA degradation machinery explain why hPNPase cannot compensate for the lack of EcPNPase. However, the *PNPT1*_*Ec*_ strain does not simply recapitulate the phenotype of strains with loss of function mutations in the *pnp* gene. Phenotypic traits due to EcPNPase lack, like the fitness decrease, the aggregation in minimal medium, the sensitivity to agents causing—directly or indirectly—DNA damage like UV, zeocin, and H_2_O_2_, the low frequency of spontaneous mutations and the ROS accumulation are all exacerbated by hPNPase, and more than 200 genes are differentially expressed between the *PNPT1*_*Ec*_ and the Δ*pnp* strains*.* These findings are consistent with hPNPase retaining some activity in *E. coli* cells, and we speculate that it may retain RNA binding activity, which is not necessarily linked to catalysis^28^. Binding by hPNPase could interfere with RNA function and RNA degradation pathways active in the absence of EcPNPase worsening the effect of the Δ*pnp* mutation. A limitation of this work is that we have not assayed the effect of a *PNPT1*_*Ec*_ mutation eliminating the RNA binding domains of hPNPase. This experiment, which we plan to do, will allow to test our hypothesis that at least some *PNPT1*_*Ec*_ phenotypic traits may be due to ectopic RNA binding.

Even assuming that indirect effects linked to the *PNPT1*_*Ec*_ slow growth play a major role in RNA stabilization and only a fraction of the RNAs responsive to hPNPase are actually direct targets of the enzyme, the effect of hPNPase on overall RNA stability remains striking, with many RNAs (i.e. about the 42%) more stable in presence of hPNPase in *PNPT1*_*Ec*_ than in Δ*pnp* mutant. Paradoxical RNA stabilizing effect exerted by *E. coli* exonucleases, among which EcPNPase, was already reported^1,6,7,70^ and it has been confirmed by our data, as around 700 RNAs (i.e. about the 19%) were more stable in the presence of EcPNPase in the *pnp*^+^ than in its absence in the Δ*pnp* strain.

Intriguingly, overexpressed hPNPase promotes ROS in Hela cells through an unknown mechanism^55^. Our data suggest that hPNPase may exert the same effect in *E. coli*, making unlikely that hPNPase-dependent ROS induction may require the interaction with specific human proteins or RNAs.

In the *PNPT1*_*Ec*_ strain, the SOS response is constitutively active. Since the SOS response is elicited by RecA*^71^, i.e. a filament of RecA protein assembled on ssDNA, this indicates that hPNPase stimulates ssDNA accumulation. A relevant source of ssDNA in the *PNPT1*_*Ec*_ are most likely the R-loops that are abundant in such strain. Accordingly, some phenotypic traits of the *PNPT1*_*Ec*_, and in particular SOS induction and slower growth in aerobiosis, are also shown by *rnhA* mutants lacking RNase HI^56,57^. In principle, hPNPase may indirectly affect R-loop level by down-regulating the expression of the RNase HI *rnhA* gene or of *recG* and *topA* genes encoding RecG and topoisomerase I, respectively, which have also been implicated in R-loop homeostasis^72^. However, transcriptomic data show that *rnhA, recG* and *topA* genes are all equally expressed in presence or absence of hPNPase, ruling out their down-regulation by hPNPase, at least at the level of mRNA synthesis and/or degradation. Moreover, *rnhA* mutants do not grow in anaerobiosis^57^, whereas the *PNPT1*_*Ec*_ does, suggesting that the presence of hPNPase does not abolish RNase HI activity. hPNPase may indirectly promote R-loops because it causes ROS accumulation, which is known to induce R-loops in human cells^34,60,61,73–75^. However, R-loops are abundant also in anaerobiosis, suggesting that other mechanisms can contribute to their formation. Factors binding the nascent RNA co-transcriptionally, like Rho, have been shown to play a pivotal role in avoiding excessive R-loop formation^76–78^. hPNPase ectopic binding of RNAs emerging from transcriptional bubbles may interfere with the anti-R-loop activity of such factors. On the contrary, in human cells, hPNPase loading onto the nascent transcript would prevent the formation of R-loops or destabilize them because hPNPase would bring to the transcript the SUV3 helicase, which is able to denature RNA–DNA hybrids^33,34,79^.

Despite the comparable level of the *PNPT1*_*Ec*_ and *pnp* long mRNAs in the *PNPT1*_*Ec*_ and *pnp*^+^ strains, respectively, hPNPase was less abundant than EcPNPase. EcPNPase negatively regulates its own expression through complex post-transcriptional mechanisms modulating *pnp* mRNA translation and stability, which mainly occur at the level of the mRNA 5′-UTR^1^. Thus, while it is not surprising that replacing EcPNPase with hPNPase impacts autoregulation, given the dissimilar activity of the two proteins, we would have expected mRNA stabilization and increased translation in presence of hPNPase, as shown by strains expressing mutated EcPNPases^8,12,39,80–82^. It is possible that despite codon optimization for *E. coli*, translation elongation of the *PNPT1*_*Ec*_ mRNA is not efficient and impairs hPNPase expression even though autoregulation is not stringent. The abundance of the short *PNPT1*_*Ec*_ RNA in the *PNPT1*_*Ec*_ (Fig. 2A), which could derive from either premature transcription termination and/or degradation of untranslated transcripts^83,84^, is consistent with this hypothesis.

## Methods

### Bacterial strains and plasmids

*E. coli* strains and plasmids used in this work are listed in Table 1. Coordinates of *E. coli* genes refer to MG1655 reference genome (GenBank accession number U00096.3) unless differently stated. C-6001 was obtained by replacing in C-1a^42^ the *pnp* ORF (region 3309033–3311168) with a *PNPT1*_*Ec*_:kanR cassette by λRED recombination^41^. *PNPT1*_*Ec*_:kanR was constructed by assembling amplicons deriving from PCR amplification with ad hoc primers of *PNPT1*_*Ec*_ and kanR cassette on pET28b-H6-hPNPase and pKD13, respectively^14,41^. *PNPT1*_*Ec*_ encodes hPNPase ORF with the *E. coli* codon usage and without the first 45 amino acids corresponding to the mitochondrial localization signal. All other strains were similarly constructed by assembling in vitro, by PCR amplification, recombinant cassettes and inserting them into the *E. coli* chromosome by λRED recombination. FLP recombination was exploited to remove antibiotic resistance genes in all strains^41^. The PNPases expressed by C-6009 and C-6011 have 6 residues long His-tag at the C-terminus. For pETD-h*PNP* construction, *PNPT1*_*Ec*_ was amplified by PCR on pET28b-H6-hPNPase with proper primers and cloned in pETDuet-1 digested with *Nco*I*-Eco*RI*,* by NEBuilder Hi-Fi DNA Assembly (New England Biolabs).

Bacterial cultures were grown in LD broth, M9 supp, i.e. M9 minimal medium with 0.4% (w/v) glucose and 2.5% LD^85^ or M9/2.5 i.e. M9 with a ca. 40-fold reduction of the phosphate content (from 57 to 1.3 mM) and 0.4% glucose. When needed, media were supplemented with 100 µg/ml ampicillin, 50 µg/ml kanamycin, 0.1 mM IPTG. Growth at low oxygen in liquid cultures was performed by inoculating bacteria in 15 ml conical Falcon tubes filled to the brim with LD medium with screwed caps and further sealed with parafilm. Incubation was performed static at 37 °C. In this condition, given low solubility of oxygen in culture medium and poor diffusion, bacteria below about 1 mm grow anaerobically^86^. Growth in anaerobiosis in solid media was performed by incubating the plates at 37 °C in an airtight jar in presence of an Aerogen (Oxoid) sachet.

### Western blotting and hPNPase protein stability

Proteins were extracted by sonication from mid-exponential cultures grown in LD at 37 °C as described^87^. For hPNPase stability determination, overnight cultures were diluted to OD_600_ = 0.02 in LD broth and grown at 37 °C, aerated. At OD_600_ = 0.6, 25 µg/ml streptomycin was added, and 2 ml samples were collected every 20 min for 1 h. Samples were pelleted and resuspended in protein loading sample buffer (50 mM Tris–HCl pH 6.8, 2% SDS, 0.1% bromophenol blue, and 10% glycerol) to OD_600_ = 25. After 3 min boiling, protein samples (15 µl, corresponding to 0.375 OD_600_) were loaded onto 10 or 12% SDS–polyacrylamide gel, as stated in Figure legends. After the run, the gels were blotted onto PROTRAN nitrocellulose membranes (GE Healthcare) and the membranes stained with Ponceau S solution (Sigma). Immunodecoration was performed with one of the following antibodies diluted in Blotto^87^ as indicated in figure legends: (i) monoclonal anti-hPNPase (Anti-hPNP: PNPase (G-11) sc-365049; Santa Cruz Biotechnology); (ii) polyclonal anti-hPNPase (PA5-22397; Invitrogen); (iii) polyclonal anti-L4 or S3 ribosomal proteins (kindly provided by C. Gualerzi); (iv) polyclonal anti-EcPNPase^88^ (v) anti-His tag antibody (MA-1–21315; Invitrogen).

### RNA extraction and analysis by Northern blot and RT-qPCR

Procedures for RNA extraction, electrophoresis on denaturing polyacrylamide and agarose gels, Northern blotting, staining of the RNA on filters with methylene blue and 5′-end labelling of oligonucleotides with [γ^32P^]-ATP and T4 polynucleotide kinase were previously described^89,90^. Oligonucleotide probes used for Northern blotting were CPXQ (4416706–4416727); RECA (1059766–1059783); SOXS (4245855–4245873); 1842 (5S-specific primer)^91^. RT-PCR (Reverse Transcription- PCR) was performed on RNA extracted as for the RNA-Seq. 2 μg were reverse-transcribed with Superscript III Reverse Transcriptase (Invitrogen). 1:40 cDNA dilution was used for PCR with primers specific for either the *PNPT1*_*Ec*_ mRNA or 16S rRNA (used as reference gene^87^), respectively. RT-qPCR (Reverse Transcription- quantitative PCR) was performed on RNA extracted from three independent cultures for each strain. 1 μg of RNA was reverse-transcribed with Superscript III Reverse Transcriptase (Invitrogen) and 1:10 cDNA dilution was used for RT-qPCR with SYBR Premix Ex Taq (Takara) with primers specific for the *PNPT1*_*Ec*_ mRNA. Two technical duplicates were performed for each biological replicate and reactions were run in a CFX Connect Real-Time PCR Detection System (Bio-Rad). 16S rRNA was used as reference gene to normalize the results and calculate the relative fold change in gene expression using the CFX*maestro* Software (Bio-Rad).

### RNA-Seq analysis of global transcript levels and stability

Global transcript levels were determined by extracting total RNA as described^92^ from three replicate cultures of C-1a, C-5691 and C-6001 grown in LD at 37 °C up to OD_600_ = 0.8. Each RNA sample was qualitatively and quantitatively checked on Agilent 2100 Bioanalyzer RNA Pico Chip (Agilent, Santa Clara, CA, USA). Directional RNA-Seq libraries were prepared from 1 µg of total RNA using the TruSeq Stranded Total RNA with Illumina Ribo-Zero Plus Sample Prep Kit (Illumina, San Diego, CA) according to the manufacturer’s protocol. Sequencing was performed on an Illumina NextSeq 500 platform (Illumina, San Diego, CA) with 12 M 2 × 75 paired end reads for each sample. For RNA stability analysis, three replicate cultures of C-1a, C-5691 and C-6001 were grown in LD at 37 °C up to OD_600_ = 0.8 and rifampicin (0.4 mg/ml) was added to the cultures. Samples for RNA extraction were taken immediately before (time 0) and 4 min after the addition of the antibiotic. The RNA was processed for library preparation and sequencing on Illumina NextSeq 500 platform as described before. For all datasets, sequencing reads were mapped on the *E. coli* K12 MG1655 RefSeq genome assembly (NCBI accession number NC_000913.3) with bowtie2^93^ in “very sensitive” mode allowing a maximum insert size of 1000 bps. Expression quantification was performed with respect to the latest gene annotation retrieved from NCBI (GCF_000005845.2_ASM584v2). Transcript levels of each gene were defined as the number of reads overlapping its transcribed region, computed with bedtools^94^. Differential expression analysis was performed with edgeR^95^. Initial read counts were normalized by trimmed mean of M values (TMM), with default parameters. Differentially expressed genes were identified by the quasi-likelihood (QL) F-test of edgeR (glmQLFfit and glmQLFtest functions, with default parameters). We selected as differentially expressed all genes with an adjusted p-value (FDR) < 0.01 and a log fold change higher than 1 or lower than -1 (that is, showing at least a two-fold change of expression). For RNA stability analysis, raw reads counts were normalized by library size and counts per million employed in subsequent comparisons.

### ROS assay

200 µl of exponential cultures (OD_600_ = 0.8) grown in LD at 37 °C were harvested, resuspended in 200 µl of PBS containing 1.0 mM DCFH-DA (Sigma-Aldrich) and incubated 30 min at 37 °C. The samples were then washed threefold with PBS and transferred into a 96-well plate. The OD_600_ and fluorescence at 488/530 (excitation/emission) nm were measured with an EnSight (PerkinElmer) plate reader. Fluorescence was normalized by the OD_600_ of the tested culture sample.

### Detection of R-loops

Total nucleic acids extraction from 10 ml overnight cultures grown in LD at 37 °C and R-loop detection were performed essentially as described^78^. 10 μg samples were immobilized on Hybond-N^+^ nylon membrane (PerkinElmer) using a Dot-blot apparatus (Hybri-Dot Manifold). RNase treatment was performed before immobilization on the membrane by incubating the samples with 20 U of Ribonuclease H (NEB) 3 h at 37 °C or 1U of RNase III (Epicentre) for 1 h at 37 °C. In this condition, RNase III visibly degrades 8 µg of total RNA as determined in preliminary experiments (not shown). After UV crosslinking, the filters were immunodecorated with the RNA–DNA hybrid specific S9.6 antibody (EDM Millipore) diluted 1:5000 in blocking buffer (5% milk in TBST). Incubation was performed either 3 h at room temperature or overnight at 4 °C with similar results. After washing, incubation with the secondary anti-mouse IgG antibody (Thermo Fisher Scientific) was performed 1 h at room temperature. Signal detection was carried out via chemiluminescence (PDS standard ECL GeneSpin) using ChemiDoc Touch Imaging System (Bio-Rad) for membrane imaging. After the acquisition, the membrane was washed in water, soaked in 5% acetic acid for 15 min with shaking, and the nucleic acids stained with 0.05% methylene blue dissolved in 0.5 M sodium acetate buffer (pH 5.2).

### Fluctuation test

A fluctuation test was performed to evaluate streptomycin resistance mutation rate (µ, i.e. the probability of mutation per cell per division). Independent cultures of C-1a, C-5691 and C-6001 (N = 12) were incubated overnight at 37 °C and titers were measured by plating proper dilutions on LD-Agar plates. The undiluted cultures were also plated on LD-Agar plates containing 0.025 mg/ml streptomycin. Mutations per culture value (m) was calculated as described^96^ from the median of the titer obtained in LD plate (N) and the median of the number of mutated colonies obtained on streptomycin plates (r), by resolving the equation: $r - 1.24m - m*{\text{ln}}\left( m \right) = 0$. µ was calculated as the ratio between m and the median number of colonies obtained in permissive conditions (without streptomycin).

### Statistical analysis

The replicate number for each sample are indicated in the figure legends, when appropriate. Statistical significance was determined by two-tailed Student's t-test when comparing two groups or ANOVA with post-hoc Tuckey test when comparing three or more groups. Pearson’s Chi-square test was used to compare distributions. The differences in means were considered statistically significant at *P* < 0.05.

## Supplementary Information

Supplementary Figures.

Supplementary Table S1.

Supplementary Table S2.

## Data Availability

All relevant data are shown. Original images of gels and filters are shown in Supplementary Fig. S4. RNA-Seq raw data are available at GEO repository (accession n. GSE211960, token axujemsonzcpdov, and GSE221825, token ejgtwucwrxyzrkr).
