# Supplementary material for: Human PNPase causes RNA stabilization and accumulation of R-loops in the Escherichia coli model system
Source: Sci Rep. 2023 Jul 21;13:11771. doi: 10.1038/s41598-023-38924-x (PMC10362022; doi:10.1038/s41598-023-38924-x)
Supplement: Supplementary file 1 — Supplementary Figures. [file 41598_2023_38924_MOESM1_ESM.pdf]

```
EcPNPase        ------------------MLNPIVRKFQYGQHTVTLETGMMARQATAAVMVSMDDTAVFV
SoPNPase        FNVRAMAQTHVSQKHAHDSLQPYSVKIPFGDRHILVETGHIGRQASGAVTVTDGETIVYT
hPNPase         -----------------------AVAVDLGNRKLEISSGKLARFADGSAVVQSGDTAVMV
                                          .  *:: : :.:* :.* * .:. *  .:* * .

EcPNPase        TVVGQKKAKPGQDFFPLTVNYQERTYAAGRIPGSFFRREGRPSEGETLIARLIDRPIRPL
SoPNPase        TVCMSDIPSEPSDFFPLSVNYQERFSAAGRTSGGFFKREGRPKDNEVLVCRLIDRPLRTT
hPNPase         TAVSKTKPS-PSQFMPLVVDYRQKAAAAGRIPTNYLRREVGTSDKEILTSRIIDRSIRPL
                *.  .  ..  .:*:** *:*:::  **** . .:::**  ..: * * .*:***.:*.

EcPNPase        FPEGFVNEVQVIATVVSVNPQVNPDIVAMIGASAALSLSGIPFNGPIGAARVGYINDQYV
SoPNPase        MLKGFYHETQILSWVLSYDGLHPPDALAVTAAGIAVALSELPHTKPVAGVRVGLVGGKYI
hPNPase         FPAGYFYDTQVLCNLLAVDGVNEPDVLAINGASVALSLSDIPWNGPVGAVRIGIIDGEYV
                :  *:  :.*::. ::: :    ** :*: .*. *::** :* . *:...*:* :..:*:

EcPNPase        LNPTQDELKESKLDLVVAGT-EAAVLMVESEAQLLSEDQMLGAVVFGHEQQQVVIQNINE
SoPNPase        VNPTTNEMENSELDLVVAGT-DSAILMIEGYCNFLPEEKLLEAVEVGQDAVRAICKEVEA
hPNPase         VNPTRKEMSSSTLNLVVAGAPKSQIVMLEASAENILQQDFCHAIKVGVKYTQQIIQGIQQ
                :*** .*:..* *:*****: .: ::*:*. .: : ::.:  *: .* .  : : : ::

EcPNPase        LVKEAGKPRWDWQPEPVNEALNARVAALAEARLSDAYRITDKQERYAQVDVIKSETIATL
SoPNPase        LVEKCGKPKMIDAIKLPPPELYKHVEEIAGDELVHALQIKKKLPRRKALVSLEEKVVDIL
hPNPase         LVKETGVTKRTPQKLFTPSPEIVKYTHKLAMERLYAVFTDYEHDKVSRDEAVNKIRLDTE
                **:: * .:              :       .   *     :  :      ::.  :

EcPNPase        LAEDET-----------------------------------------------LDENE
SoPNPase        TQRGYVGKSVATVIPETLPDLYVDEEEDEEVVVDGEVDEGDVHIKPSPKNCTPLLFSEVD
hPNPase         EQLKEKFP----------------------------------------------EADPYE
                                                                        .  :

EcPNPase        LGEILHAIEKNVVRSRVLAGEPRIDGREKDMIRGLDVRTGVLPRTHGSALFTRGETQALV
SoPNPase        VKLVFKETTSKYLRKRIVEGGRRSDGRTSEGVRPINSRCGLLPRAHGSALFTRGETQALA
hPNPase         IIESFNVVAKEVFRSIVLNEYKRCDGRDLTSLRNVSCEVDMFKTLHGSALFQRGQTQVLC
                :   ::   .: .*. ::    * ***    :* :. . .::   ****** **:**.*

EcPNPase        TATLGTARDAQVLD---ELMG-ERTDTFLFHYNFPPYSVGETGMVGSPKRREIGHGRLAK
SoPNPase        VVTLGDKQMAQRID---TLEGDDESKRFYLQYSFPPSCVGEAGRVGAPSRREIGHGTLAE
hPNPase         TVTFDSLESGIKSDQVITAINGIKDKNFMLHYEFPPYATNEIGKVTGLNRRELGHGALAE
                ..*:.  . .   *      .  . . * ::*.*** ...* * * . .***:*** **:

EcPNPase        RGVLAVMPDMDKFPYTVRVVSEITESNGSSSMASVCGASLALMDAGVPIKAAVAGIAMGL
SoPNPase        RALEPILPSEDEFPYTVRVESNITESNGSSSMASVCGGSLALLDAGVPVKCPIAGIAMGM
hPNPase         KALYPVIPR--DFPFTIRVTSEVLESNGSSSMASACGGSLALMDSGVPISSAVAGVAIGL
                :.: .::*   .**:*:** *:: **********.**.****:*:***:...:**:*:*:

EcPNPase        VKEG-DNY------VVLSDILGDEDHLGDMDFKVAGSRDGISALQMDIKIEGITKEIMQV
SoPNPase        VLDT-KEFGGDGTPLILSDITGSEDASGDMDFKVAGNADCVTAFQMDIKVGGITLSVMKQ
hPNPase         VTKTDPEKGEIEDYRLLTDILGIEDYNGDMDFKIAGTNKGITALQADIKLPGIPIKIVME
                * .   :        :*:** * **  ******:**. . ::*:* ***: **. .::

EcPNPase        ALNQAKGARLHILGVMEQAINAPRGDISEFAPRIHTIKINPDKIKDVIGKGGSVIRALTE
SoPNPase        ALLQAKDGRKIILAEMSKCLPPPSKTLSIYAPLIHVMKVRPEKVNLIIGSGGKTVRSIIE
hPNPase         AIQQASVAKKEILQIMNKTISKPRASRKENGPVVETVQVPLSKRAKFVGPGGYNLKKLQA
                *: **. .:  **  *.: :  *    .  .* :..:::  .*   .:* **  :: :

EcPNPase        ET-GTTIEIEDDGTVKIAATDGEKAKHAIRRIEEIT-----AEIEVGRVYT-GKVTRIVD
SoPNPase        ATGVEAIDTQDDGTVKITARDLESLEKSKAMIANLT-----MVPTIGDIYRNCEIKSIAP
hPNPase         ET-GVTISQVDEETFSVFAPTPSAMHEARDFITEICKDDQEQQLEFGAVYT-ATITEIRD
                 *   :*.  *: *..: *   .  ..:   * ::          .* :*    :. *

EcPNPase        FGAFVAIGG-GKEGLVHISQIADKRVEKVTDY-LQMGQEVPVKVLEVD-RQGRIRLSIKE
SoPNPase        YGAFVEIAP-GREGLCHISELSPSYLAKAEDA-FKVGDRVDVKLIEVN-EKGQLRLSRRA
hPNPase         TGVMVKLYPNMTAVLLHNTQLDQRKIKHPTALGLEVGQEIQVKYFGRDPADGRMRLSRKV
                 *.:* :       * * :::    : :     :::*:.: ** :  :  .*::*** :

EcPNPase        ATEQSQPAAAPEAPAAEQGE----------------------------------------
SoPNPase        LIPDTDPLVPSTKARPTTATQESSASKKVELTKVKVNASKEDVYPPGKVVRDGPFINKDR
hPNPase         LQSPATTVVRTLNDRSSIVMGEPISQSSSNSQ----------------------------
                    : . . .    .

EcPNPase        ------------------------
SoPNPase        LKKGSKAMSNAPNQSDSSVVNKEG
hPNPase         ------------------------
```

Supplemental Fig. S1. PNPase alignment. The sequences of *E. coli* (EcPNPase), *Homo sapiens* (hPNPase) and *Spinacia olearia* chloroplast (SoPNPase) have been aligned with ClustalW. The first 60 and 44 residues of SoPNPase and hPNPase, respectively, containing organelle localization signals, are not shown. RPH1 (yellow), RPH2 (grey) and KH-S1 (cyano) domains of EcPNPase are 44/65 %, 47/62 % and 32/47 % identical/similar with hPNPase and 42/63 %, 56/69 % and 36/58 % identical/similar with SoPNPase.

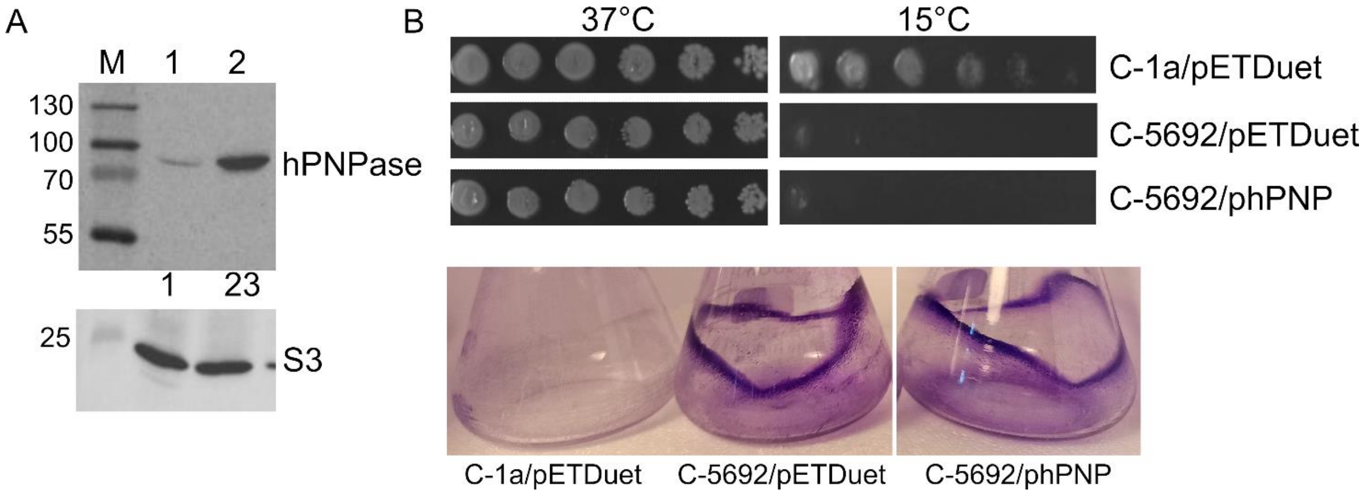

Supplemental Fig. S2. Growth at low temperature and adhesion of strains expressing hPNPase from a multicopy plasmid. A. Western blotting of proteins extracted from exponential cultures of C-6001 (1) and C-5692/phPNP (2). The proteins (20 µg) were run on 10% polyacrylamide-SDS gel and blotted onto a nitrocellulose membrane. The filter was cut, and the lower part was hybridized with S3- specific antibodies to check loading and the upper portion with hPNPase- specific monoclonal antibodies. The position of MW markers (M) is reported in kDa on the left. The relative intensity of hPNPase signals measured with Image Lab software (Bio-Rad) is indicated below the lanes. B, upper part, serial dilutions of overnight cultures were plated on LD-agar supplemented with 100 µg/ml ampicillin. Plates were incubated overnight at 37 °C or 7 days at 15 °C. Lower part, cultures were grown at 37 °C with shaking in M9 supplemented with 0.4% glucose, 2.5% LD and 100 µg/ml ampicillin. After 8 hours, cultures were discarded, and the flasks stained with crystal violet to colour adherent cells. In both cases, identical results were obtained also with growth media containing 0.1 mM IPTG.

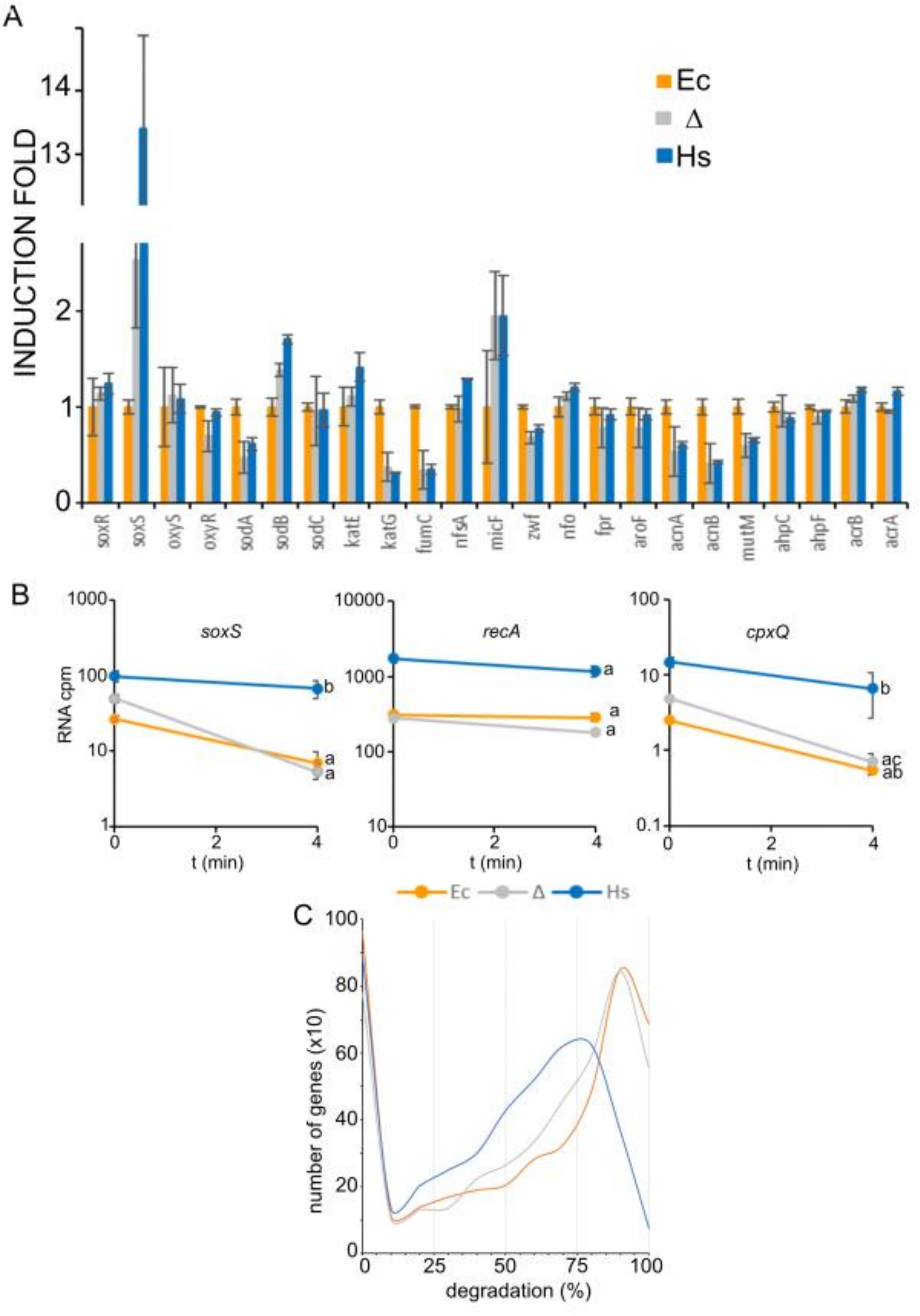

Supplemental Fig. S3. Expression of genes belonging to SoxRS regulon and overall RNA stability. RNA-Seq data were used to measure induction fold (A) and stability (B, C). Raw reads counts were normalized by library size and counts per million (cpm) employed to compare samples. A. Gene expression was measured by RNA-Seq on triplicate cultures of C-1a (Ec), C-5691 (Δ) and C-6001 (Hs) as explained in Methods. For each gene, the value obtained in one of the Ec duplicates was arbitrarily set to 1 and used as reference to calculate induction fold. Bars represent average (N = 3) with StD. B. RNA decay curves. Symbols represent average (N = 3) with StD of RNA cpm in samples

extracted immediately before (time 0) and 4 min after the addition of rifampicin to the cultures. Half-lives calculated with curves denoted by the same letter are not significantly different according to ANOVA with Tuckey post hoc test. The half-life of *soxS, recA and cpxQ* RNAs in Ec, Δ and Hs, respectively, are: 2.1±0.5, 1.2±0.1 and 7.4±2.3 min, *soxS*; 16.6±10.9, 6.4±0.5 and 7.3±0.9 min, *recA*; 1.8±0.2, 1.4±0.2 and 5.7±2.7 min, *cpxQ*. C. Distribution of the percentage of degradation of *E. coli* transcripts (i. e. % RNA lost between T0 and T4) in Ec (orange line), Δ (grey line) and Hs (blue line).

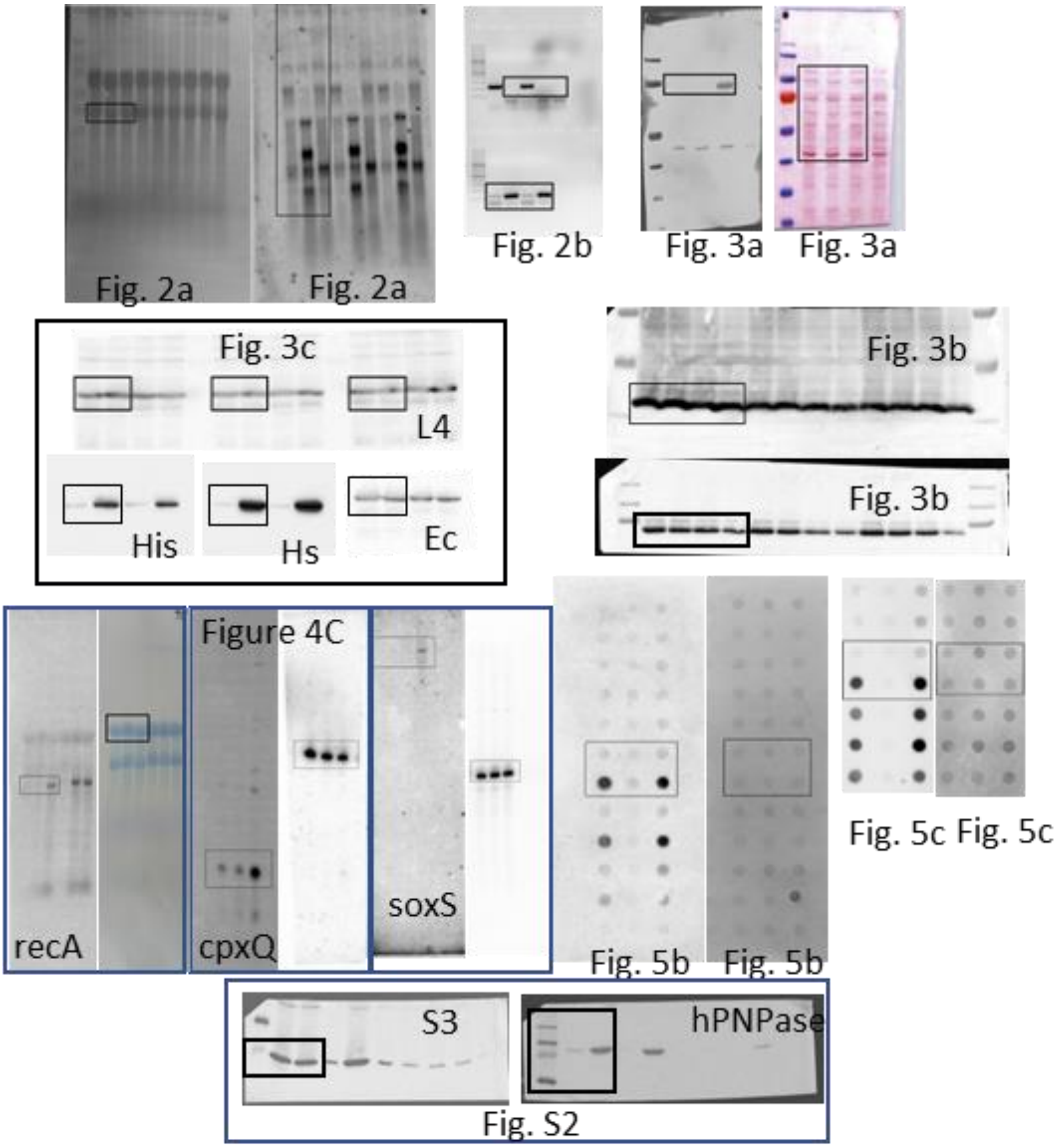

Supplemental Fig. S4. Original images of gels and filters shown in the figures.
