# Supplementary material for: Human PNPase causes RNA stabilization and accumulation of R-loops in the Escherichia coli model system
Source: Sci Rep. 2023 Jul 21;13:11771. doi: 10.1038/s41598-023-38924-x (PMC10362022; doi:10.1038/s41598-023-38924-x)
Supplement: Supplementary file 2 — Supplementary Table S1. [file 41598_2023_38924_MOESM2_ESM.pdf]

Table S1. Differentially expressed genes

Δpnp vs . pnp^{+}*					
**Gene**	**logFC**	**logCPM**	**F**	**PValue**	**FDR**
aceB	-1.5	5.5	33.6	0.0	0.0
acnA	-1.2	8.1	23.0	0.0	0.0
acnB	-1.6	11.3	34.2	0.0	0.0
acpS	-1.2	5.7	113.6	0.0	0.0
acs	-2.6	8.3	45.2	0.0	0.0
actP	-2.2	6.1	55.8	0.0	0.0
adhE	-1.0	9.1	35.9	0.0	0.0
agaV	-1.2	3.1	38.4	0.0	0.0
ais	1.0	4.2	36.9	0.0	0.0
aldA	-3.3	8.3	68.8	0.0	0.0
alsA	-2.0	4.9	57.3	0.0	0.0
alsB	-2.3	5.6	46.4	0.0	0.0
alsC	-2.6	3.9	53.3	0.0	0.0
alsE	-2.4	4.7	34.0	0.0	0.0
alsK	-1.7	4.7	52.4	0.0	0.0
alsR	-1.2	7.4	36.2	0.0	0.0
ansP	1.1	5.3	44.1	0.0	0.0
arcA	-1.1	9.3	36.3	0.0	0.0
argT	-2.3	6.3	72.4	0.0	0.0
ariR	-3.1	3.4	103.4	0.0	0.0
arnA	1.3	6.2	119.9	0.0	0.0
arnB	1.3	6.2	71.6	0.0	0.0
arnC	1.4	5.5	99.4	0.0	0.0
arnD	1.2	4.6	109.0	0.0	0.0
arnE	1.1	4.1	57.2	0.0	0.0
arnT	1.4	5.0	159.6	0.0	0.0
aroF	-1.1	6.8	151.3	0.0	0.0
asnU	1.1	1.5	37.6	0.0	0.0
aspT	1.3	5.3	73.2	0.0	0.0
aspU	1.4	2.0	28.7	0.0	0.0
astA	-3.5	4.6	57.2	0.0	0.0
astB	-3.0	4.9	47.2	0.0	0.0
astC	-4.0	4.8	58.4	0.0	0.0
astD	-3.3	5.1	60.0	0.0	0.0
astE	-3.1	4.1	91.3	0.0	0.0
atpC	-1.1	9.9	54.0	0.0	0.0
azoR	-1.1	5.2	34.2	0.0	0.0
bamD	-1.4	9.3	195.9	0.0	0.0
bamE	-1.3	8.3	117.5	0.0	0.0
betA	-2.2	6.5	45.3	0.0	0.0
betB	-2.2	7.1	46.0	0.0	0.0
betI	-1.9	6.4	34.3	0.0	0.0
betT	-1.6	6.3	67.2	0.0	0.0
bglJ	1.4	4.4	47.5	0.0	0.0
bsmA	1.5	9.4	142.8	0.0	0.0

hPNP vs . pnp^{+}*					
**Gene**	**logFC**	**logCPM**	**F**	**PValue**	**FDR**
abgA	-1.8	3.2	38.7	0.0	0.0
aceA	-2.0	5.3	27.7	0.0	0.0
aceB	-1.5	5.5	35.3	0.0	0.0
aceE	1.3	10.8	32.2	0.0	0.0
aceF	1.1	10.1	31.3	0.0	0.0
acnA	-1.0	8.1	17.6	0.0	0.0
acnB	-1.6	11.3	33.3	0.0	0.0
acpS	-1.4	5.7	144.9	0.0	0.0
acrE	1.0	2.6	22.5	0.0	0.0
acs	-3.4	8.3	74.8	0.0	0.0
actP	-2.4	6.1	68.8	0.0	0.0
adeQ	-1.4	4.0	99.5	0.0	0.0
adhE	-1.2	9.1	44.1	0.0	0.0
agaV	-2.2	3.1	112.1	0.0	0.0
agp	-2.1	7.7	90.0	0.0	0.0
ais	1.8	4.2	113.6	0.0	0.0
aldA	-3.5	8.3	78.0	0.0	0.0
aldB	-1.9	5.1	94.7	0.0	0.0
alsA	-2.6	4.9	86.3	0.0	0.0
alsB	-3.8	5.6	102.7	0.0	0.0
alsC	-3.7	3.9	93.0	0.0	0.0
alsE	-3.6	4.7	68.5	0.0	0.0
alsK	-2.1	4.7	74.6	0.0	0.0
alsR	-2.1	7.4	101.8	0.0	0.0
ansP	1.0	5.3	43.6	0.0	0.0
aphA	-2.8	8.7	93.8	0.0	0.0
araB	-1.0	3.9	24.7	0.0	0.0
araC	-1.1	6.9	26.4	0.0	0.0
araD	-1.2	2.0	25.5	0.0	0.0
araF	-1.6	4.2	73.8	0.0	0.0
argT	-1.6	6.3	38.6	0.0	0.0
arnA	1.4	6.2	155.5	0.0	0.0
arnB	2.0	6.2	169.3	0.0	0.0
arnC	1.8	5.5	159.3	0.0	0.0
arnD	1.5	4.6	155.0	0.0	0.0
arnT	1.4	5.0	159.0	0.0	0.0
aspA	-1.5	12.8	69.3	0.0	0.0
aspT	1.0	5.3	45.8	0.0	0.0
aspU	1.5	2.0	31.2	0.0	0.0
asr	1.4	1.6	36.5	0.0	0.0
astA	-3.3	4.6	52.7	0.0	0.0
astB	-3.0	4.9	47.2	0.0	0.0
astC	-3.6	4.8	49.6	0.0	0.0
astD	-3.3	5.1	60.1	0.0	0.0
astE	-3.2	4.1	99.3	0.0	0.0

hPNP vs . Δpnp*					
**Gene**	**logFC**	**logCPM**	**F**	**PValue**	**FDR**
aceE	1.3	10.8	36.1	0.0	0.0
aceF	1.6	10.1	67.9	0.0	0.0
agp	-1.3	7.7	34.6	0.0	0.0
alx	1.0	3.8	36.7	0.0	0.0
aphA	-1.8	8.7	43.2	0.0	0.0
araF	-1.0	4.2	31.1	0.0	0.0
ariR	2.1	3.4	49.8	0.0	0.0
asnA	1.3	7.4	61.1	0.0	0.0
aspA	-1.7	12.8	91.0	0.0	0.0
atoC	-1.1	5.3	40.9	0.0	0.0
bamE	1.0	8.3	70.4	0.0	0.0
bdm	2.7	1.1	75.9	0.0	0.0
betI	1.6	6.4	23.9	0.0	0.0
betT	1.0	6.3	27.7	0.0	0.0
bfd	-1.1	5.9	40.4	0.0	0.0
bglJ	-1.4	4.4	44.6	0.0	0.0
bhsA	2.0	4.1	105.4	0.0	0.0
bsmA	-1.5	9.4	148.2	0.0	0.0
chiP	-1.9	6.4	73.4	0.0	0.0
cho	1.5	5.4	132.7	0.0	0.0
cirA	-1.7	7.0	24.6	0.0	0.0
cpxP	1.9	6.7	262.1	0.0	0.0
cpxQ	2.2	3.8	120.7	0.0	0.0
degP	2.3	9.5	56.9	0.0	0.0
dhaM	-1.1	8.9	29.4	0.0	0.0
dicB	1.8	-0.1	28.1	0.0	0.0
dinB	1.7	5.6	199.1	0.0	0.0
dinD	1.2	4.9	76.0	0.0	0.0
dinF	1.1	6.0	120.7	0.0	0.0
dinI	3.7	6.4	279.0	0.0	0.0
dinQ	1.4	6.2	66.6	0.0	0.0
dmlR	-1.3	6.1	36.4	0.0	0.0
dppA	-1.8	12.4	31.6	0.0	0.0
dppB	-1.7	9.5	36.6	0.0	0.0
dppC	-1.6	8.9	34.3	0.0	0.0
dppD	-1.7	9.4	45.0	0.0	0.0
dppF	-1.8	9.3	39.9	0.0	0.0
entA	-1.0	4.5	39.2	0.0	0.0
entB	-1.2	4.9	26.4	0.0	0.0
entC	-1.3	5.3	36.3	0.0	0.0
entE	-1.4	5.2	64.6	0.0	0.0
entH	-1.1	3.4	26.2	0.0	0.0
fepA	-1.3	5.9	96.2	0.0	0.0
fepC	-1.1	5.3	30.9	0.0	0.0
fes	-1.4	5.4	117.1	0.0	0.0

btsT	-4.3	7.5	115.6	0.0	0.0
chiP	1.4	6.4	43.2	0.0	0.0
chiQ	1.4	3.6	40.8	0.0	0.0
cirA	2.9	7.0	65.6	0.0	0.0
citC	1.4	2.0	36.7	0.0	0.0
clpA	-1.2	9.4	34.0	0.0	0.0
clpB	-2.5	11.0	20.7	0.0	0.0
cnoX	-1.6	7.9	28.0	0.0	0.0
cpxQ	1.1	3.8	23.3	0.0	0.0
crfC	-1.1	4.1	61.4	0.0	0.0
csgD	1.6	1.2	27.8	0.0	0.0
csiD	-1.5	3.1	74.4	0.0	0.0
csiE	-1.5	7.9	39.4	0.0	0.0
csiR	-1.2	6.5	61.7	0.0	0.0
csrB	1.6	4.3	76.5	0.0	0.0
cvpA	1.1	6.8	48.4	0.0	0.0
cycA	-1.5	8.3	72.5	0.0	0.0
cydA	1.4	10.4	38.9	0.0	0.0
cydB	1.3	9.7	26.8	0.0	0.0
cydX	1.3	7.4	25.3	0.0	0.0
cyoA	-2.4	11.1	61.3	0.0	0.0
cyoB	-2.1	11.0	27.2	0.0	0.0
cyoC	-1.9	9.5	18.4	0.0	0.0
cyoD	-1.9	9.4	18.5	0.0	0.0
cyoE	-1.7	9.9	21.0	0.0	0.0
cysK	-1.1	7.7	40.9	0.0	0.0
dadA	-2.9	9.8	19.2	0.0	0.0
dadX	-2.6	9.1	17.7	0.0	0.0
dapD	-1.2	8.4	42.3	0.0	0.0
dauA	1.3	7.8	146.6	0.0	0.0
dctA	-1.6	10.9	40.9	0.0	0.0
dcuA	1.5	10.6	82.8	0.0	0.0
ddpA	-1.1	3.7	35.9	0.0	0.0
ddpX	-1.3	2.4	18.5	0.0	0.0
deaD	2.1	8.5	48.6	0.0	0.0
deoA	-1.2	8.7	67.2	0.0	0.0
deoB	-1.0	9.5	67.4	0.0	0.0
deoC	-1.2	8.5	30.0	0.0	0.0
dgcT	1.5	7.0	42.1	0.0	0.0
dgcZ	1.1	6.9	51.5	0.0	0.0
dgoR	-1.1	5.3	26.8	0.0	0.0
dhaK	-3.6	8.2	50.1	0.0	0.0
dhaL	-3.4	7.4	64.7	0.0	0.0
dhaM	-2.3	8.9	117.0	0.0	0.0
dmlA	3.8	7.4	276.0	0.0	0.0
dnaG	1.1	8.8	20.4	0.0	0.0
dppB	-1.4	9.5	24.3	0.0	0.0
dppC	-1.2	8.9	21.5	0.0	0.0
dsdA	-1.7	5.6	34.8	0.0	0.0
atoA	-1.2	7.3	57.1	0.0	0.0
atoB	-1.2	7.7	76.6	0.0	0.0
atoC	-1.6	5.3	86.8	0.0	0.0
atoD	-1.0	8.2	42.7	0.0	0.0
atoE	-1.2	7.6	62.6	0.0	0.0
atoS	-1.1	5.5	18.7	0.0	0.0
azoR	-1.2	5.2	40.2	0.0	0.0
azuC	2.5	-0.3	22.6	0.0	0.0
bamD	-1.1	9.3	112.2	0.0	0.0
bdm	2.7	1.1	68.8	0.0	0.0
betA	-1.4	6.5	18.9	0.0	0.0
bglH	1.3	2.9	19.2	0.0	0.0
bhsA	2.5	4.1	142.3	0.0	0.0
bipA	1.3	9.7	23.6	0.0	0.0
blr	1.3	5.0	30.1	0.0	0.0
bssR	1.3	2.3	37.5	0.0	0.0
bssS	1.4	7.1	105.9	0.0	0.0
btsT	-4.1	7.5	110.1	0.0	0.0
btuB	-1.0	8.4	87.6	0.0	0.0
cbl	1.0	3.4	34.5	0.0	0.0
cbrB	1.0	5.3	28.4	0.0	0.0
cbrC	1.1	5.0	68.5	0.0	0.0
cdd	-1.0	9.7	26.9	0.0	0.0
cdgI	1.9	2.0	35.9	0.0	0.0
cho	1.9	5.4	204.7	0.0	0.0
citC	1.5	2.0	44.3	0.0	0.0
clpB	-2.8	11.0	26.2	0.0	0.0
cnoX	-1.8	7.9	33.6	0.0	0.0
cnu	1.0	4.6	16.6	0.0	0.0
cpdB	-1.5	8.7	121.5	0.0	0.0
cpxP	2.4	6.7	377.7	0.0	0.0
cpxQ	3.3	3.8	221.5	0.0	0.0
csgC	1.5	1.5	20.4	0.0	0.0
csgD	2.0	1.2	47.8	0.0	0.0
csgE	2.3	0.1	25.9	0.0	0.0
csiD	-1.3	3.1	59.0	0.0	0.0
csiE	-2.2	7.9	78.4	0.0	0.0
cspC	-1.2	6.4	24.9	0.0	0.0
cspD	-1.1	9.4	37.2	0.0	0.0
cspI	3.1	2.3	54.6	0.0	0.0
csrB	1.0	4.3	30.6	0.0	0.0
cstA	-1.8	8.1	34.3	0.0	0.0
curA	-1.6	6.1	79.2	0.0	0.0
cvpA	1.4	6.8	74.9	0.0	0.0
cyaR	1.4	2.7	17.2	0.0	0.0
cycA	-1.3	8.3	52.4	0.0	0.0
cyoA	-1.3	11.1	19.5	0.0	0.0
cytR	-1.4	8.7	182.4	0.0	0.0
dacD	1.1	4.5	30.1	0.0	0.0
fhuA	-1.4	9.3	69.6	0.0	0.0
fhuB	-1.0	6.7	34.7	0.0	0.0
fhuC	-1.2	6.2	60.3	0.0	0.0
fhuD	-1.2	5.5	41.9	0.0	0.0
fhuF	-1.3	7.3	136.9	0.0	0.0
focA	-1.2	8.0	98.9	0.0	0.0
frdA	-1.2	9.2	71.8	0.0	0.0
frdB	-1.2	7.4	80.7	0.0	0.0
frdC	-1.3	6.7	94.4	0.0	0.0
frdD	-1.2	7.1	68.1	0.0	0.0
ftnA	1.3	6.6	68.6	0.0	0.0
fucA	-1.4	4.6	58.2	0.0	0.0
fucI	-1.5	6.3	119.0	0.0	0.0
fucO	-1.5	8.0	96.7	0.0	0.0
fucP	-1.6	4.8	85.7	0.0	0.0
galE	-1.7	8.3	61.0	0.0	0.0
galK	-1.4	8.1	100.8	0.0	0.0
galP	-1.4	7.5	31.3	0.0	0.0
galS	-1.8	7.8	137.6	0.0	0.0
galT	-1.6	7.7	72.2	0.0	0.0
glpA	-3.2	9.1	153.4	0.0	0.0
glpB	-3.2	8.9	284.0	0.0	0.0
glpC	-3.1	8.6	320.0	0.0	0.0
glpD	-1.2	11.8	34.3	0.0	0.0
glpF	-1.4	11.3	28.3	0.0	0.0
glpK	-1.7	12.5	28.8	0.0	0.0
glpQ	-2.6	12.6	332.6	0.0	0.0
glpT	-2.6	12.7	264.3	0.0	0.0
glpX	-1.3	7.9	41.6	0.0	0.0
gutM	-1.4	4.7	135.1	0.0	0.0
hscC	-1.2	5.6	95.0	0.0	0.0
ivy	1.5	6.5	73.2	0.0	0.0
lamB	-1.2	11.3	62.3	0.0	0.0
ldtC	1.1	4.2	29.8	0.0	0.0
lexA	1.6	8.6	143.6	0.0	0.0
malK	-1.0	10.3	54.1	0.0	0.0
malM	-1.5	9.2	118.0	0.0	0.0
melR	-1.4	7.0	34.9	0.0	0.0
mglA	-1.9	10.1	64.1	0.0	0.0
mglB	-1.4	11.4	41.1	0.0	0.0
mglC	-1.9	9.1	51.7	0.0	0.0
micL	1.7	3.9	134.6	0.0	0.0
mliC	1.3	4.2	48.9	0.0	0.0
mocA	-1.3	5.3	22.5	0.0	0.0
mqsA	1.5	7.0	117.1	0.0	0.0
mqsR	1.4	6.6	68.3	0.0	0.0
mscS	1.2	7.3	78.1	0.0	0.0
nagB	-1.1	8.1	27.3	0.0	0.0
nanA	-1.8	10.2	38.8	0.0	0.0

dsdX	-1.9	4.4	36.8	0.0	0.0
dusB	1.5	9.1	18.0	0.0	0.0
ebgA	-1.1	5.2	109.2	0.0	0.0
ebgC	-1.5	2.2	63.1	0.0	0.0
entA	1.8	4.5	107.7	0.0	0.0
entB	2.0	4.9	73.6	0.0	0.0
entC	2.3	5.3	96.6	0.0	0.0
entD	1.2	3.1	47.4	0.0	0.0
entE	2.3	5.2	161.9	0.0	0.0
entF	1.5	6.3	69.5	0.0	0.0
entH	1.7	3.4	54.7	0.0	0.0
entS	1.8	5.2	38.9	0.0	0.0
eutG	1.2	1.6	19.7	0.0	0.0
fadA	-2.1	6.3	85.2	0.0	0.0
fadB	-3.5	7.2	93.6	0.0	0.0
fadD	-1.6	8.4	174.1	0.0	0.0
fadE	-1.9	6.3	102.3	0.0	0.0
fadH	-1.9	5.7	65.0	0.0	0.0
fadI	-2.5	6.1	61.0	0.0	0.0
fadJ	-1.6	7.2	56.4	0.0	0.0
fadK	1.4	6.1	90.9	0.0	0.0
fbaA	-1.3	10.1	76.8	0.0	0.0
fdoG	-1.7	11.2	43.9	0.0	0.0
fdoH	-1.7	9.4	47.9	0.0	0.0
fdoI	-1.6	8.7	79.2	0.0	0.0
feoA	1.3	6.0	77.8	0.0	0.0
feoB	1.1	8.2	60.9	0.0	0.0
fepA	1.9	5.9	187.0	0.0	0.0
fepB	1.2	5.3	30.3	0.0	0.0
fepC	1.1	5.3	31.5	0.0	0.0
fepD	1.7	5.8	217.3	0.0	0.0
fepE	1.4	3.4	23.4	0.0	0.0
fepG	1.3	5.2	70.8	0.0	0.0
fes	2.2	5.4	257.6	0.0	0.0
ffh	-1.0	9.1	81.2	0.0	0.0
ffs	1.6	2.8	30.8	0.0	0.0
fhuA	1.4	9.3	74.7	0.0	0.0
fhuB	1.6	6.7	77.7	0.0	0.0
fhuC	1.5	6.2	90.9	0.0	0.0
fhuD	1.2	5.5	41.1	0.0	0.0
fhuE	1.4	5.3	108.7	0.0	0.0
fhuF	1.1	7.3	89.9	0.0	0.0
flgB	-2.2	0.6	27.2	0.0	0.0
flgD	-1.8	2.2	92.6	0.0	0.0
flgE	-1.8	3.4	211.0	0.0	0.0
flgF	-1.1	2.5	38.1	0.0	0.0
flgG	-1.7	2.4	78.7	0.0	0.0
fliH	-1.2	1.4	24.9	0.0	0.0
fliI	-1.1	1.9	23.3	0.0	0.0
dadA	-2.5	9.8	14.7	0.0	0.0
dadX	-2.7	9.1	18.8	0.0	0.0
dapD	-1.3	8.4	49.3	0.0	0.0
dauA	1.4	7.8	165.4	0.0	0.0
dctA	-2.1	10.9	65.0	0.0	0.0
ddpA	-1.8	3.7	83.8	0.0	0.0
ddpB	-1.0	2.5	19.7	0.0	0.0
ddpX	-1.5	2.4	24.1	0.0	0.0
deaD	2.4	8.5	61.2	0.0	0.0
degP	1.2	9.5	16.6	0.0	0.0
deoA	-1.6	8.7	107.6	0.0	0.0
deoB	-1.1	9.5	83.2	0.0	0.0
deoC	-1.6	8.5	51.8	0.0	0.0
deoD	-1.1	9.7	49.0	0.0	0.0
dgcT	1.2	7.0	29.7	0.0	0.0
dgcZ	1.9	6.9	140.7	0.0	0.0
dgoD	-1.3	3.7	78.6	0.0	0.0
dgoK	-1.1	4.1	38.1	0.0	0.0
dgoR	-1.4	5.3	47.3	0.0	0.0
dhaK	-4.1	8.2	62.8	0.0	0.0
dhaL	-4.1	7.4	89.8	0.0	0.0
dhaM	-3.4	8.9	234.4	0.0	0.0
dinB	2.1	5.6	280.1	0.0	0.0
dinD	1.6	4.9	129.3	0.0	0.0
dinF	1.5	6.0	198.5	0.0	0.0
dinG	1.1	6.5	131.6	0.0	0.0
dinI	3.7	6.4	279.6	0.0	0.0
dinQ	1.4	6.2	59.9	0.0	0.0
dmlA	3.2	7.4	199.6	0.0	0.0
dmlR	-1.9	6.1	78.2	0.0	0.0
dnaG	1.2	8.8	23.4	0.0	0.0
dppA	-2.9	12.4	74.0	0.0	0.0
dppB	-3.1	9.5	107.9	0.0	0.0
dppC	-2.8	8.9	100.3	0.0	0.0
dppD	-2.6	9.4	94.6	0.0	0.0
dppF	-2.5	9.3	76.5	0.0	0.0
dsdA	-1.6	5.6	31.3	0.0	0.0
dsdX	-1.5	4.4	23.5	0.0	0.0
dtpB	-1.9	8.8	76.1	0.0	0.0
dtpD	1.0	5.9	35.2	0.0	0.0
dusB	2.0	9.1	33.7	0.0	0.0
ebgA	-1.5	5.2	182.7	0.0	0.0
ebgC	-2.1	2.2	112.2	0.0	0.0
efeO	-1.1	8.1	51.4	0.0	0.0
emrY	1.1	2.6	49.0	0.0	0.0
eno	-1.0	10.9	53.2	0.0	0.0
eptA	1.1	5.9	53.6	0.0	0.0
eutG	1.2	1.6	22.4	0.0	0.0
fadA	-2.1	6.3	87.4	0.0	0.0
nanE	-1.8	7.5	38.6	0.0	0.0
nanK	-1.6	7.7	45.4	0.0	0.0
nanT	-1.9	8.7	43.1	0.0	0.0
narW	-1.1	2.5	26.8	0.0	0.0
narY	-1.0	3.7	49.7	0.0	0.0
narZ	-1.0	5.5	62.4	0.0	0.0
nupG	-1.6	8.6	92.3	0.0	0.0
ompF	-2.0	12.2	115.6	0.0	0.0
oppA	-1.2	10.0	28.3	0.0	0.0
oppC	-1.2	7.3	36.7	0.0	0.0
oppD	-1.3	7.4	27.4	0.0	0.0
osmB	2.2	5.4	144.6	0.0	0.0
osmY	1.4	8.4	41.1	0.0	0.0
patZ	-1.1	8.5	66.6	0.0	0.0
pepE	-1.4	7.4	65.4	0.0	0.0
pnp	-3.9	9.6	640.7	0.0	0.0
psiE	1.8	2.6	107.2	0.0	0.0
pspA	1.2	6.4	59.9	0.0	0.0
pspB	1.1	4.4	68.2	0.0	0.0
pspD	1.1	3.1	26.9	0.0	0.0
pspG	1.8	2.3	56.0	0.0	0.0
psuG	-1.2	3.9	37.7	0.0	0.0
ptsG	1.1	8.5	57.9	0.0	0.0
recA	3.2	10.3	395.7	0.0	0.0
recN	3.6	8.2	773.7	0.0	0.0
recX	2.1	4.0	133.3	0.0	0.0
rhaR	-1.1	5.3	53.6	0.0	0.0
ribB	-2.1	9.2	143.5	0.0	0.0
rihA	-1.5	9.0	88.4	0.0	0.0
rihC	-1.2	7.3	61.1	0.0	0.0
rpoE	1.8	9.5	137.1	0.0	0.0
rseA	1.7	9.3	139.7	0.0	0.0
rseB	1.1	8.5	106.4	0.0	0.0
rseD	1.5	7.1	112.4	0.0	0.0
ruvA	1.1	6.6	106.0	0.0	0.0
sbmA	1.1	6.2	139.1	0.0	0.0
sbmC	2.0	6.4	167.6	0.0	0.0
sfmC	2.0	1.6	22.7	0.0	0.0
sgbU	-1.8	2.1	93.4	0.0	0.0
soxS	2.2	5.8	103.9	0.0	0.0
spy	2.4	5.5	353.2	0.0	0.0
sraG	-1.8	8.0	150.6	0.0	0.0
srlA	-1.5	7.7	43.4	0.0	0.0
srlB	-1.4	5.9	52.8	0.0	0.0
srlD	-1.3	7.9	53.0	0.0	0.0
srlE	-1.6	7.5	110.0	0.0	0.0
srlR	-1.0	6.7	132.1	0.0	0.0
sulA	3.9	8.4	761.5	0.0	0.0
tdcA	-4.9	8.4	834.6	0.0	0.0

ftnA	-1.7	6.6	109.9	0.0	0.0
ftp	1.4	5.6	35.8	0.0	0.0
fumA	-1.8	9.9	43.9	0.0	0.0
fumC	-1.8	6.2	36.1	0.0	0.0
gadW	-1.1	4.4	88.0	0.0	0.0
gadY	-1.2	3.0	34.5	0.0	0.0
galS	-1.4	7.8	87.6	0.0	0.0
gapA	-1.2	11.8	40.8	0.0	0.0
gcd	-1.0	7.0	20.3	0.0	0.0
ghrB	-1.5	7.2	132.6	0.0	0.0
ghxP	1.3	5.9	23.5	0.0	0.0
glcC	-1.9	6.4	71.5	0.0	0.0
glmY	1.6	4.2	23.2	0.0	0.0
glmZ	1.2	7.1	22.2	0.0	0.0
glnU	1.5	10.0	20.6	0.0	0.0
glnW	1.6	10.1	24.8	0.0	0.0
glpD	-1.2	11.8	33.8	0.0	0.0
glpF	-1.5	11.3	33.2	0.0	0.0
glpK	-1.6	12.5	25.5	0.0	0.0
gltA	-2.9	11.0	36.9	0.0	0.0
gltI	-2.8	9.3	36.2	0.0	0.0
gltJ	-2.0	6.8	26.3	0.0	0.0
gltK	-1.9	6.5	31.1	0.0	0.0
gltL	-1.7	7.4	19.3	0.0	0.0
glyV	2.2	8.8	22.5	0.0	0.0
glyX	2.1	8.5	24.6	0.0	0.0
glyY	2.4	8.7	28.0	0.0	0.0
gpmM	-1.1	6.7	69.2	0.0	0.0
groL	-1.8	12.3	16.2	0.0	0.0
groS	-2.0	10.6	19.3	0.0	0.0
grpE	-2.3	10.6	56.3	0.0	0.0
grxA	1.2	2.8	23.8	0.0	0.0
grxB	-1.0	6.1	16.6	0.0	0.0
gshA	-1.3	8.9	146.0	0.0	0.0
gspJ	1.0	2.9	31.4	0.0	0.0
gstB	-1.2	6.8	102.6	0.0	0.0
hcaR	-2.3	6.8	60.5	0.0	0.0
hdeA	-1.6	5.5	55.4	0.0	0.0
hdeB	-2.2	3.7	66.3	0.0	0.0
hdeD	-1.5	3.6	52.1	0.0	0.0
hisC	-1.2	4.7	23.2	0.0	0.0
hisG	-1.2	5.1	22.8	0.0	0.0
hisJ	-1.2	6.5	55.1	0.0	0.0
hisP	-1.1	4.8	46.0	0.0	0.0
hofB	1.2	3.4	21.6	0.0	0.0
hslO	-1.2	7.9	23.5	0.0	0.0
hslR	-1.3	6.9	24.3	0.0	0.0
hslU	-1.6	9.5	27.6	0.0	0.0
hslV	-1.7	8.1	42.5	0.0	0.0

fadB	-2.8	7.2	65.9	0.0	0.0
fadE	-1.1	6.3	34.4	0.0	0.0
fadH	-2.1	5.7	77.8	0.0	0.0
fadI	-2.4	6.1	53.4	0.0	0.0
fadJ	-2.2	7.2	99.5	0.0	0.0
fadK	1.3	6.1	85.2	0.0	0.0
fbaA	-1.6	10.1	119.0	0.0	0.0
fcl	1.3	1.6	16.5	0.0	0.0
fdhE	-1.3	8.3	87.8	0.0	0.0
fdoG	-1.8	11.2	49.1	0.0	0.0
fdoH	-2.2	9.4	72.7	0.0	0.0
fdoI	-2.1	8.7	129.5	0.0	0.0
ffh	-1.0	9.1	81.6	0.0	0.0
fis	1.9	8.1	27.2	0.0	0.0
flgB	-2.0	0.6	23.6	0.0	0.0
flgD	-1.9	2.2	104.2	0.0	0.0
flgE	-1.2	3.4	113.1	0.0	0.0
flgF	-1.1	2.5	37.7	0.0	0.0
flgG	-1.4	2.4	60.3	0.0	0.0
fliE	1.2	1.0	16.3	0.0	0.0
fliF	-1.0	2.3	31.7	0.0	0.0
fliR	1.2	2.3	37.2	0.0	0.0
frwB	-1.2	2.3	66.8	0.0	0.0
frwC	-1.3	3.0	60.4	0.0	0.0
ftnB	1.2	7.8	69.9	0.0	0.0
ftp	1.2	5.6	26.2	0.0	0.0
fucA	-1.3	4.6	51.6	0.0	0.0
fucI	-1.7	6.3	151.6	0.0	0.0
fucK	-1.3	5.8	126.9	0.0	0.0
fucO	-1.3	8.0	71.4	0.0	0.0
fucP	-1.3	4.8	51.7	0.0	0.0
fucR	-1.4	6.6	85.8	0.0	0.0
fumA	-2.5	9.9	81.4	0.0	0.0
fumC	-1.8	6.2	36.6	0.0	0.0
gadE	-1.4	2.3	16.4	0.0	0.0
galE	-2.4	8.3	118.7	0.0	0.0
galK	-1.6	8.1	147.2	0.0	0.0
galP	-2.1	7.5	71.4	0.0	0.0
galS	-3.2	7.8	395.2	0.0	0.0
galT	-2.1	7.7	123.2	0.0	0.0
gapA	-1.4	11.8	60.9	0.0	0.0
ghrB	-1.4	7.2	110.5	0.0	0.0
ghxP	1.7	5.9	41.8	0.0	0.0
glmY	2.2	4.2	42.2	0.0	0.0
glmZ	1.2	7.1	21.7	0.0	0.0
glnU	1.6	10.0	21.8	0.0	0.0
glnW	1.8	10.1	30.0	0.0	0.0
glpA	-3.5	9.1	179.2	0.0	0.0
glpB	-3.7	8.9	357.8	0.0	0.0

tdcB	-5.0	6.4	913.1	0.0	0.0
tdcC	-3.0	6.4	158.5	0.0	0.0
tdcD	-3.9	5.8	131.8	0.0	0.0
tdcE	-3.4	6.5	87.8	0.0	0.0
tdcF	-3.1	3.7	90.9	0.0	0.0
tdcG	-2.5	4.6	45.2	0.0	0.0
tisB	5.4	8.1	738.0	0.0	0.0
tnaA	-5.2	12.6	266.1	0.0	0.0
tnaB	-5.0	9.4	190.7	0.0	0.0
tnaC	-5.4	9.5	175.0	0.0	0.0
torY	1.3	2.7	32.9	0.0	0.0
trxC	1.0	6.2	44.3	0.0	0.0
tsgA	2.2	5.7	101.5	0.0	0.0
tsx	-1.3	11.1	94.4	0.0	0.0
ucpA	-1.0	9.8	48.6	0.0	0.0
uhpT	2.8	6.2	306.4	0.0	0.0
umuC	2.8	6.1	288.3	0.0	0.0
umuD	4.3	5.2	742.0	0.0	0.0
uvrA	1.2	8.4	129.7	0.0	0.0
uxaB	1.3	4.9	118.9	0.0	0.0
uxuB	-1.2	7.1	57.3	0.0	0.0
wcaE	2.5	0.0	46.2	0.0	0.0
xdhC	-1.2	4.1	24.7	0.0	0.0
yafP	1.4	3.6	109.4	0.0	0.0
yaiY	1.2	3.8	106.8	0.0	0.0
yajI	1.0	4.1	88.9	0.0	0.0
ybdZ	-1.3	2.6	24.4	0.0	0.0
ybgS	1.3	4.2	108.8	0.0	0.0
ybiV	1.1	5.2	61.0	0.0	0.0
ycaM	-1.3	7.4	44.9	0.0	0.0
ycfJ	1.8	4.9	249.6	0.0	0.0
ycgZ	2.0	3.5	51.1	0.0	0.0
ycjM	-1.0	4.2	43.8	0.0	0.0
ycjN	-1.2	3.4	37.4	0.0	0.0
ydcH	-1.4	6.3	26.0	0.0	0.0
ydcS	-1.4	5.9	34.6	0.0	0.0
ydcT	-1.4	4.7	46.6	0.0	0.0
ydcV	-1.5	4.2	43.6	0.0	0.0
yddA	-1.6	4.7	37.7	0.0	0.0
yddB	-1.3	5.2	40.1	0.0	0.0
ydeN	-1.2	7.2	46.8	0.0	0.0
ydeP	1.0	3.9	52.7	0.0	0.0
ydeQ	1.6	0.9	26.1	0.0	0.0
ydeT	2.6	2.3	136.2	0.0	0.0
ydfD	2.1	0.0	36.5	0.0	0.0
ydiN	2.1	0.9	51.8	0.0	0.0
ydjM	1.4	4.4	144.6	0.0	0.0
yebE	1.0	5.1	91.1	0.0	0.0
yebF	1.7	7.7	114.7	0.0	0.0

hxpA	-1.3	6.9	37.2	0.0	0.0
ibsD	1.4	5.5	53.0	0.0	0.0
icd	-1.2	11.3	27.0	0.0	0.0
iclR	-1.1	6.6	126.4	0.0	0.0
ilvE	-1.3	8.1	40.2	0.0	0.0
ilvM	-1.2	4.8	46.5	0.0	0.0
ilvX	-1.9	1.8	57.5	0.0	0.0
iroK	-2.2	3.1	50.7	0.0	0.0
iscA	-1.0	8.2	67.7	0.0	0.0
iscR	-1.6	8.0	48.8	0.0	0.0
iscS	-1.2	9.3	59.1	0.0	0.0
iscU	-1.1	8.2	60.8	0.0	0.0
ispE	1.2	9.3	39.7	0.0	0.0
katG	-1.7	8.7	58.7	0.0	0.0
kbaZ	-1.3	4.1	34.3	0.0	0.0
kbl	-1.3	9.5	99.6	0.0	0.0
kbp	-1.4	7.5	69.4	0.0	0.0
kdgK	-1.0	6.2	115.2	0.0	0.0
kefB	-1.5	5.9	40.6	0.0	0.0
kefG	-1.4	4.6	27.8	0.0	0.0
kgtP	-2.9	6.7	306.5	0.0	0.0
ldhA	-1.3	6.7	24.9	0.0	0.0
leuW	1.8	10.1	28.9	0.0	0.0
livJ	-1.4	5.7	66.8	0.0	0.0
lldP	-3.1	10.5	37.0	0.0	0.0
lon	-1.4	10.3	19.8	0.0	0.0
lsrA	-1.5	4.0	62.5	0.0	0.0
lsrB	-1.5	3.1	66.4	0.0	0.0
lsrC	-1.3	3.5	41.5	0.0	0.0
lsrD	-1.1	2.7	35.7	0.0	0.0
lsrF	-1.5	2.9	42.4	0.0	0.0
lsrG	-1.1	2.2	23.1	0.0	0.0
lsrR	-1.1	5.0	20.5	0.0	0.0
luxS	-1.8	8.3	153.2	0.0	0.0
malE	-1.0	11.0	40.3	0.0	0.0
malM	-1.0	9.2	55.3	0.0	0.0
malP	-1.1	9.5	71.9	0.0	0.0
manX	-1.1	9.3	23.1	0.0	0.0
manY	-1.6	8.7	47.6	0.0	0.0
manZ	-1.7	9.3	69.6	0.0	0.0
mdtJ	1.5	4.2	21.9	0.0	0.0
metH	-1.2	8.0	41.4	0.0	0.0
metT	1.8	10.3	27.8	0.0	0.0
metV	4.7	7.0	273.5	0.0	0.0
metW	4.9	8.8	166.8	0.0	0.0
metZ	4.7	7.9	252.3	0.0	0.0
mglA	-2.0	10.1	68.7	0.0	0.0
mglB	-2.0	11.4	77.8	0.0	0.0
mglC	-1.8	9.1	46.3	0.0	0.0
glpC	-3.7	8.6	438.6	0.0	0.0
glpD	-2.4	11.8	126.6	0.0	0.0
glpF	-3.0	11.3	110.3	0.0	0.0
glpK	-3.3	12.5	95.7	0.0	0.0
glpQ	-2.7	12.6	355.5	0.0	0.0
glpT	-2.7	12.7	287.5	0.0	0.0
glpX	-1.9	7.9	87.4	0.0	0.0
gltA	-2.9	11.0	38.6	0.0	0.0
gltI	-2.9	9.3	39.4	0.0	0.0
gltJ	-2.4	6.8	36.9	0.0	0.0
gltK	-2.5	6.5	50.7	0.0	0.0
gltL	-2.3	7.4	35.0	0.0	0.0
glyA	-1.2	9.7	89.4	0.0	0.0
glyV	2.5	8.8	28.3	0.0	0.0
glyX	2.4	8.5	30.6	0.0	0.0
glyY	2.7	8.7	35.0	0.0	0.0
gmd	1.1	1.7	16.0	0.0	0.0
gpmA	-1.1	9.9	41.5	0.0	0.0
gpmM	-1.1	6.7	79.9	0.0	0.0
gpr	-1.7	7.3	90.8	0.0	0.0
gpsA	-1.4	9.2	70.6	0.0	0.0
groL	-2.1	12.3	21.4	0.0	0.0
groS	-2.1	10.6	21.4	0.0	0.0
grpE	-2.3	10.6	58.4	0.0	0.0
grxA	1.2	2.8	26.1	0.0	0.0
gshA	-1.4	8.9	159.5	0.0	0.0
gspJ	1.1	2.9	34.9	0.0	0.0
guaB	1.6	8.4	31.0	0.0	0.0
hcaR	-2.0	6.8	47.6	0.0	0.0
hdeA	-2.0	5.5	80.8	0.0	0.0
hdeB	-2.7	3.7	97.3	0.0	0.0
hdeD	-1.6	3.6	53.9	0.0	0.0
hisP	-1.1	4.8	46.8	0.0	0.0
hofB	1.0	3.4	16.2	0.0	0.0
hslO	-1.2	7.9	20.9	0.0	0.0
hslR	-1.1	6.9	17.4	0.0	0.0
hslU	-1.8	9.5	33.5	0.0	0.0
hslV	-1.7	8.1	42.3	0.0	0.0
htpG	-2.1	10.3	16.2	0.0	0.0
htrE	1.0	4.3	18.6	0.0	0.0
hxpA	-1.3	6.9	38.5	0.0	0.0
icd	-1.0	11.3	20.6	0.0	0.0
ilvD	-1.0	7.2	23.7	0.0	0.0
ilvE	-1.6	8.1	59.9	0.0	0.0
ilvM	-1.4	4.8	61.5	0.0	0.0
ilvX	-1.1	1.8	24.4	0.0	0.0
iroK	-2.0	3.1	42.8	0.0	0.0
iscA	-1.2	8.2	99.3	0.0	0.0
iscR	-1.8	8.0	66.6	0.0	0.0
yebG	2.8	7.7	514.3	0.0	0.0
yedR	1.1	2.1	30.7	0.0	0.0
yeeA	1.4	5.7	169.7	0.0	0.0
yejG	1.6	5.7	66.1	0.0	0.0
yfbM	-1.1	2.6	43.5	0.0	0.0
yfdV	1.6	1.1	33.4	0.0	0.0
yfeC	-1.2	7.2	74.9	0.0	0.0
yfeD	-1.1	7.1	73.7	0.0	0.0
yfeK	2.2	4.8	116.7	0.0	0.0
yfeS	1.4	6.4	87.4	0.0	0.0
yfeY	1.2	7.0	145.5	0.0	0.0
ygaC	1.1	5.3	85.4	0.0	0.0
ygcN	-1.1	6.7	52.8	0.0	0.0
ygcO	-1.4	3.7	62.7	0.0	0.0
ygcP	-1.4	4.6	116.4	0.0	0.0
ygcW	-1.5	1.3	24.4	0.0	0.0
ygeV	-1.7	8.2	47.8	0.0	0.0
ygiM	2.0	7.0	242.6	0.0	0.0
yhcG	-1.1	5.4	42.3	0.0	0.0
yhcH	-1.9	7.8	78.9	0.0	0.0
yhfA	-1.1	7.1	24.4	0.0	0.0
yhjV	-1.3	7.0	67.8	0.0	0.0
yiaO	-1.0	2.6	29.6	0.0	0.0
yigI	1.7	5.5	80.4	0.0	0.0
yjbE	1.4	1.6	32.6	0.0	0.0
ymdF	1.2	3.3	33.0	0.0	0.0
ymdG	1.7	1.1	31.6	0.0	0.0
ymgA	1.9	3.8	53.2	0.0	0.0
ymgC	1.7	2.2	33.0	0.0	0.0
ymgD	2.9	6.2	127.9	0.0	0.0
ymgG	3.1	5.6	158.1	0.0	0.0
ymgI	2.9	3.6	226.4	0.0	0.0
ynaJ	1.0	6.0	45.0	0.0	0.0
ynfQ	2.0	1.6	24.0	0.0	0.0
yohJ	1.1	3.0	29.7	0.0	0.0
ypeC	1.7	5.0	97.3	0.0	0.0
ypfG	1.4	5.0	64.4	0.0	0.0
yqeB	-1.3	6.3	23.6	0.0	0.0
yqeC	-1.8	5.8	23.3	0.0	0.0
ysaB	1.3	2.8	37.3	0.0	0.0
ytfQ	-1.5	7.2	32.8	0.0	0.0
ytfR	-1.3	5.9	31.0	0.0	0.0
ytfT	-1.4	4.6	31.2	0.0	0.0
ytjA	1.5	6.1	41.7	0.0	0.0
yzgL	-1.6	7.5	44.3	0.0	0.0

*pnp^+, C-1a; Δ*pnp*, C-5691; *hPNP*, C-6001

mgrR	1.1	3.2	23.4	0.0	0.0
mgsA	-1.1	7.4	37.6	0.0	0.0
mgtA	-1.1	7.3	101.6	0.0	0.0
mhpR	-3.0	6.0	40.6	0.0	0.0
micA	-1.1	5.2	90.4	0.0	0.0
mngA	1.6	4.0	26.7	0.0	0.0
mngB	1.3	6.1	83.5	0.0	0.0
mnmG	1.3	9.0	70.1	0.0	0.0
moaA	-2.4	7.9	508.8	0.0	0.0
moaB	-3.5	7.7	731.4	0.0	0.0
moaC	-3.3	7.0	662.8	0.0	0.0
moaD	-3.3	6.3	615.5	0.0	0.0
moaE	-3.1	6.8	684.5	0.0	0.0
mqo	-2.8	7.7	92.2	0.0	0.0
msrA	-1.2	5.8	30.0	0.0	0.0
msrB	-1.3	7.7	64.1	0.0	0.0
mtlA	-1.6	6.6	137.9	0.0	0.0
murP	-1.2	6.1	37.0	0.0	0.0
murQ	-1.4	6.4	29.4	0.0	0.0
mutM	-1.0	6.8	58.6	0.0	0.0
nadE	-1.0	6.6	33.1	0.0	0.0
nadK	-1.1	6.4	140.3	0.0	0.0
napF	1.1	3.1	22.5	0.0	0.0
narG	-1.2	5.3	35.4	0.0	0.0
ndh	1.7	6.6	31.2	0.0	0.0
ndk	-1.6	10.1	110.8	0.0	0.0
nfuA	-1.1	8.7	35.6	0.0	0.0
nlpI	1.4	10.6	111.0	0.0	0.0
nrdD	1.5	7.8	53.9	0.0	0.0
nrdE	-1.4	5.9	101.0	0.0	0.0
nrdF	-1.2	3.9	57.4	0.0	0.0
nrdG	1.6	4.7	55.3	0.0	0.0
nrdH	-1.9	3.8	111.8	0.0	0.0
nrdI	-1.7	4.4	146.4	0.0	0.0
nth	1.0	5.8	26.9	0.0	0.0
nuoE	-1.0	9.2	43.3	0.0	0.0
nuoF	-1.0	10.0	38.5	0.0	0.0
nupG	-1.1	8.6	44.1	0.0	0.0
obgE	1.3	7.9	29.5	0.0	0.0
oppB	-1.4	6.9	29.5	0.0	0.0
oxc	1.1	2.4	26.3	0.0	0.0
paaB	1.8	0.6	26.2	0.0	0.0
paaK	-1.6	5.5	61.9	0.0	0.0
patZ	-1.3	8.5	100.1	0.0	0.0
pdxJ	-1.2	7.3	92.9	0.0	0.0
pepD	-1.2	9.0	41.0	0.0	0.0
pepN	-1.3	9.3	34.4	0.0	0.0
pgaA	3.9	9.6	741.3	0.0	0.0
pgaB	4.4	8.7	1128.1	0.0	0.0

iscS	-1.5	9.3	91.4	0.0	0.0
iscU	-1.4	8.2	100.6	0.0	0.0
ispE	1.3	9.3	50.8	0.0	0.0
katG	-2.0	8.7	78.0	0.0	0.0
kbaZ	-1.8	4.1	67.6	0.0	0.0
kbl	-1.4	9.5	115.6	0.0	0.0
kbp	-1.1	7.5	45.5	0.0	0.0
kdgK	-1.1	6.2	126.6	0.0	0.0
kduD	-1.0	4.9	25.5	0.0	0.0
kduI	-1.3	4.8	53.3	0.0	0.0
kefB	-1.1	5.9	25.6	0.0	0.0
kgtP	-2.1	6.7	175.2	0.0	0.0
lacZ	-1.3	4.7	63.3	0.0	0.0
lamB	-1.8	11.3	131.0	0.0	0.0
ldtC	1.6	4.2	59.4	0.0	0.0
leuW	1.8	10.1	27.6	0.0	0.0
lexA	1.5	8.6	127.8	0.0	0.0
lgoR	-2.4	6.1	35.5	0.0	0.0
livJ	-1.3	5.7	56.8	0.0	0.0
lldD	-2.9	9.8	19.7	0.0	0.0
lldP	-3.7	10.5	50.5	0.0	0.0
lldR	-3.3	9.0	27.4	0.0	0.0
lon	-1.5	10.3	20.8	0.0	0.0
lsrA	-2.1	4.0	118.6	0.0	0.0
lsrB	-1.9	3.1	97.3	0.0	0.0
lsrC	-1.6	3.5	57.1	0.0	0.0
lsrD	-1.3	2.7	47.5	0.0	0.0
lsrF	-1.8	2.9	62.4	0.0	0.0
lsrR	-1.7	5.0	47.6	0.0	0.0
luxS	-1.8	8.3	142.4	0.0	0.0
lysT	1.6	8.9	16.4	0.0	0.0
lysU	-1.0	9.3	28.7	0.0	0.0
lysW	1.6	7.9	18.2	0.0	0.0
lyxK	-1.5	3.0	45.3	0.0	0.0
maeB	-1.5	10.2	53.9	0.0	0.0
malE	-2.0	11.0	143.7	0.0	0.0
malF	-1.7	9.2	171.7	0.0	0.0
malG	-1.6	8.4	181.6	0.0	0.0
malK	-1.8	10.3	163.7	0.0	0.0
malM	-2.5	9.2	309.9	0.0	0.0
malP	-1.0	9.5	60.5	0.0	0.0
malT	-1.5	10.0	71.3	0.0	0.0
malX	-1.6	6.5	234.9	0.0	0.0
manY	-1.3	8.7	32.0	0.0	0.0
manZ	-1.3	9.3	42.9	0.0	0.0
marR	1.1	5.1	29.5	0.0	0.0
mdh	-1.5	10.9	58.0	0.0	0.0
mdtI	1.2	4.3	26.4	0.0	0.0
mdtJ	2.2	4.2	46.4	0.0	0.0

pgaC	4.1	7.9	1040.9	0.0	0.0	*mdtL*	-1.1	4.9	25.0	0.0	0.0
pgaD	3.3	6.6	276.9	0.0	0.0	*melR*	-1.5	7.0	39.5	0.0	0.0
pgk	-1.0	10.3	47.5	0.0	0.0	*metF*	1.0	4.0	19.4	0.0	0.0
pgl	-1.2	7.8	41.6	0.0	0.0	*metH*	-1.7	8.0	80.4	0.0	0.0
pheA	-1.3	7.2	207.7	0.0	0.0	*metN*	1.4	6.0	35.2	0.0	0.0
phoE	-1.3	5.1	26.9	0.0	0.0	*metT*	1.8	10.3	29.3	0.0	0.0
pnp	-2.2	9.6	234.8	0.0	0.0	*metV*	5.3	7.0	335.1	0.0	0.0
potB	1.1	6.5	18.7	0.0	0.0	*metW*	5.6	8.8	202.4	0.0	0.0
potC	1.1	6.3	24.9	0.0	0.0	*metY*	2.0	6.0	24.2	0.0	0.0
ppc	-1.2	8.2	19.4	0.0	0.0	*metZ*	5.3	7.9	309.6	0.0	0.0
ppsA	1.0	11.8	31.8	0.0	0.0	*mfd*	-1.0	7.9	28.9	0.0	0.0
pqqL	2.1	5.3	151.4	0.0	0.0	*mglA*	-3.9	10.1	225.6	0.0	0.0
preA	-1.4	6.8	29.7	0.0	0.0	*mglB*	-3.5	11.4	203.4	0.0	0.0
preT	-1.7	6.3	30.5	0.0	0.0	*mglC*	-3.7	9.1	168.5	0.0	0.0
priB	1.0	9.8	16.7	0.0	0.0	*mgrR*	1.8	3.2	72.6	0.0	0.0
prlC	-1.4	9.0	39.4	0.0	0.0	*mgsA*	-1.8	7.4	105.6	0.0	0.0
proV	-1.5	4.2	150.9	0.0	0.0	*mgtS*	1.5	1.8	44.3	0.0	0.0
prpR	-1.3	4.4	46.5	0.0	0.0	*mhpR*	-2.4	6.0	27.3	0.0	0.0
pssA	-1.1	8.9	75.5	0.0	0.0	*micA*	-1.3	5.2	119.4	0.0	0.0
pstC	1.1	5.6	52.7	0.0	0.0	*micL*	1.1	3.9	60.2	0.0	0.0
ptsG	-1.4	8.5	84.8	0.0	0.0	*mliC*	1.2	4.2	43.8	0.0	0.0
putP	-2.1	7.9	25.2	0.0	0.0	*mltD*	1.3	7.9	37.2	0.0	0.0
rarD	-1.2	5.5	78.0	0.0	0.0	*mmuM*	-1.1	5.4	18.6	0.0	0.0
recN	-1.0	8.2	73.6	0.0	0.0	*mnaT*	-1.3	4.6	30.4	0.0	0.0
rhlE	1.0	5.9	19.0	0.0	0.0	*mnmG*	1.4	9.0	77.1	0.0	0.0
rhsC	1.2	5.6	133.3	0.0	0.0	*mntP*	1.4	6.0	16.8	0.0	0.0
rluB	1.5	7.7	30.6	0.0	0.0	*moaA*	-2.6	7.9	566.1	0.0	0.0
rmf	1.4	5.2	16.6	0.0	0.0	*moaB*	-3.5	7.7	751.7	0.0	0.0
rpiB	-1.6	4.3	28.5	0.0	0.0	*moaC*	-3.4	7.0	685.8	0.0	0.0
rplB	1.1	12.4	18.1	0.0	0.0	*moaD*	-3.4	6.3	648.4	0.0	0.0
rplC	1.2	12.1	20.3	0.0	0.0	*moaE*	-3.2	6.8	715.0	0.0	0.0
rplD	1.1	11.4	17.4	0.0	0.0	*mocA*	-1.8	5.3	42.9	0.0	0.0
rplI	1.0	10.9	17.7	0.0	0.0	*mqo*	-1.7	7.7	37.3	0.0	0.0
rplW	1.1	10.8	18.5	0.0	0.0	*mqsA*	1.6	7.0	126.7	0.0	0.0
rpoE	-2.0	9.5	170.4	0.0	0.0	*mqsR*	1.6	6.6	90.3	0.0	0.0
rpsF	1.1	10.4	17.4	0.0	0.0	*mtlA*	-1.8	6.6	165.3	0.0	0.0
rpsJ	1.3	11.2	20.1	0.0	0.0	*murP*	-2.0	6.1	95.9	0.0	0.0
rpsR	1.1	9.7	18.2	0.0	0.0	*murQ*	-2.6	6.4	91.6	0.0	0.0
rsd	-1.4	6.5	35.1	0.0	0.0	*murR*	-1.4	4.3	47.5	0.0	0.0
rseA	-2.1	9.3	199.2	0.0	0.0	*nadA*	1.3	4.6	59.4	0.0	0.0
rseB	-1.7	8.5	236.1	0.0	0.0	*nanA*	-2.6	10.2	74.3	0.0	0.0
rseC	-1.5	7.1	149.9	0.0	0.0	*nanE*	-2.0	7.5	47.8	0.0	0.0
rseD	-1.7	7.1	143.2	0.0	0.0	*nanK*	-1.6	7.7	43.1	0.0	0.0
rsmJ	-1.1	6.7	41.5	0.0	0.0	*nanT*	-2.8	8.7	86.8	0.0	0.0
rsxD	1.2	6.5	43.4	0.0	0.0	*narG*	-1.5	5.3	55.3	0.0	0.0
rsxE	1.1	5.5	34.2	0.0	0.0	*narW*	-1.1	2.5	22.7	0.0	0.0
rsxG	1.2	5.7	37.3	0.0	0.0	*narY*	-1.0	3.7	48.0	0.0	0.0
ryfA	1.1	6.0	71.4	0.0	0.0	*narZ*	-1.1	5.5	63.3	0.0	0.0
ryfD	-1.9	7.1	43.7	0.0	0.0	*ndh*	2.2	6.6	46.6	0.0	0.0

sdaB	1.4	8.8	40.8	0.0	0.0
sdhA	-1.6	11.4	30.1	0.0	0.0
sdhB	-1.5	10.0	20.5	0.0	0.0
sdhC	-1.6	9.4	84.6	0.0	0.0
sdhD	-1.8	9.3	73.8	0.0	0.0
serC	-1.1	7.7	76.8	0.0	0.0
sfmD	1.0	3.3	48.5	0.0	0.0
sibD	1.3	5.7	43.1	0.0	0.0
sodA	-1.4	9.3	43.6	0.0	0.0
soxS	1.1	5.8	23.9	0.0	0.0
srlA	1.3	7.7	33.2	0.0	0.0
srlB	1.4	5.9	48.0	0.0	0.0
srlD	1.5	7.9	65.4	0.0	0.0
srlE	1.2	7.5	66.0	0.0	0.0
sthA	-2.3	9.0	59.6	0.0	0.0
sucA	-1.4	12.3	19.8	0.0	0.0
suhB	2.0	8.2	24.7	0.0	0.0
talB	-1.0	10.3	47.7	0.0	0.0
tas	-1.0	7.0	26.0	0.0	0.0
tdcA	2.3	8.4	230.2	0.0	0.0
tdcB	1.4	6.4	103.1	0.0	0.0
tdcC	1.3	6.4	33.6	0.0	0.0
tdcD	1.9	5.8	38.8	0.0	0.0
tdcE	1.9	6.5	29.4	0.0	0.0
tdcF	1.5	3.7	26.4	0.0	0.0
tdcG	1.6	4.6	20.3	0.0	0.0
tdh	-1.4	9.0	74.6	0.0	0.0
thrV	2.1	3.1	18.0	0.0	0.0
thrW	1.1	5.4	17.7	0.0	0.0
tpiA	-1.2	8.9	57.3	0.0	0.0
trpA	-1.3	4.7	24.6	0.0	0.0
trpB	-1.4	5.2	34.8	0.0	0.0
trpC	-1.1	4.8	35.3	0.0	0.0
trpT	1.2	5.2	48.7	0.0	0.0
trxC	-1.9	6.2	143.0	0.0	0.0
tsx	-1.2	11.1	73.1	0.0	0.0
tyrA	-1.2	7.1	135.6	0.0	0.0
tyrB	-1.5	6.9	70.7	0.0	0.0
ubiA	-1.1	6.9	43.2	0.0	0.0
ubiC	-1.1	6.6	85.5	0.0	0.0
udp	-1.0	11.8	31.5	0.0	0.0
ugpA	-2.8	3.5	39.9	0.0	0.0
ugpB	-3.4	6.1	73.1	0.0	0.0
ugpC	-1.8	5.5	28.8	0.0	0.0
ugpE	-2.7	3.8	41.5	0.0	0.0
ugpQ	-1.1	5.9	54.1	0.0	0.0
uidA	-1.3	4.1	57.3	0.0	0.0
ung	-1.3	6.4	224.6	0.0	0.0
uspD	-1.1	7.0	20.4	0.0	0.0
ndk	-1.6	10.1	105.3	0.0	0.0
nfrB	1.1	4.6	31.0	0.0	0.0
nfuA	-1.3	8.7	44.4	0.0	0.0
nhaA	1.1	7.0	54.5	0.0	0.0
nhaR	1.0	5.8	60.1	0.0	0.0
nlpI	1.0	10.6	60.6	0.0	0.0
nrdE	-1.7	5.9	157.0	0.0	0.0
nrdF	-1.6	3.9	97.1	0.0	0.0
nrdH	-2.1	3.8	130.0	0.0	0.0
nrdI	-2.0	4.4	203.2	0.0	0.0
nth	1.1	5.8	31.2	0.0	0.0
nuoB	-1.1	9.7	78.8	0.0	0.0
nuoC	-1.2	10.2	62.0	0.0	0.0
nuoE	-1.3	9.2	66.5	0.0	0.0
nuoF	-1.3	10.0	60.9	0.0	0.0
nuoG	-1.3	10.9	44.2	0.0	0.0
nuoH	-1.2	9.4	56.9	0.0	0.0
nuoI	-1.3	8.9	68.5	0.0	0.0
nuoJ	-1.2	9.3	53.7	0.0	0.0
nuoK	-1.3	8.0	75.6	0.0	0.0
nupG	-2.7	8.6	242.2	0.0	0.0
obgE	1.3	7.9	29.4	0.0	0.0
ompF	-2.2	12.2	140.4	0.0	0.0
ompG	1.1	2.1	15.1	0.0	0.0
oppA	-2.0	10.0	74.7	0.0	0.0
oppB	-2.5	6.9	93.9	0.0	0.0
oppC	-2.2	7.3	112.1	0.0	0.0
oppD	-2.1	7.4	72.6	0.0	0.0
oppF	-1.7	7.7	52.8	0.0	0.0
ortT	1.3	3.8	20.1	0.0	0.0
osmB	2.5	5.4	185.2	0.0	0.0
oxc	1.6	2.4	60.4	0.0	0.0
paaB	1.5	0.6	18.5	0.0	0.0
paaJ	-1.1	4.9	41.7	0.0	0.0
paaK	-2.0	5.5	96.7	0.0	0.0
patZ	-2.4	8.5	306.3	0.0	0.0
pck	-1.3	11.0	43.4	0.0	0.0
pdhR	2.1	8.7	20.3	0.0	0.0
pdxI	-1.8	5.4	56.7	0.0	0.0
pdxJ	-1.3	7.3	109.6	0.0	0.0
pepD	-1.2	9.0	47.7	0.0	0.0
pepE	-2.2	7.4	154.2	0.0	0.0
pepN	-1.5	9.3	45.8	0.0	0.0
pgaA	3.6	9.6	650.2	0.0	0.0
pgaB	3.9	8.7	944.5	0.0	0.0
pgaC	3.6	7.9	843.0	0.0	0.0
pgaD	2.9	6.6	221.6	0.0	0.0
pgk	-1.3	10.3	78.0	0.0	0.0
pgl	-1.1	7.8	36.0	0.0	0.0

uspG	1.1	9.1	28.3	0.0	0.0	*pheA*	-1.3	7.2	216.0	0.0	0.0
uxaC	-1.0	6.5	23.0	0.0	0.0	*phnH*	1.3	0.9	16.7	0.0	0.0
uxuA	-1.1	7.2	34.9	0.0	0.0	*pinQ*	1.9	2.8	26.4	0.0	0.0
wcaA	1.6	2.8	37.0	0.0	0.0	*pnp*	-6.1	9.6	1270.5	0.0	0.0
wzxC	1.5	1.1	27.0	0.0	0.0	*polA*	-1.0	8.5	63.4	0.0	0.0
xapB	1.1	4.5	76.5	0.0	0.0	*potA*	1.3	7.5	16.3	0.0	0.0
xdhA	-1.2	6.3	47.9	0.0	0.0	*potB*	1.3	6.5	24.8	0.0	0.0
yadN	1.8	1.3	24.7	0.0	0.0	*potC*	1.1	6.3	27.8	0.0	0.0
yadV	1.4	2.5	38.2	0.0	0.0	*ppc*	-1.3	8.2	24.9	0.0	0.0
yafW	1.4	3.3	57.8	0.0	0.0	*pphC*	1.4	2.5	46.2	0.0	0.0
yaiO	1.1	3.3	33.9	0.0	0.0	*ppnN*	-1.5	8.5	32.1	0.0	0.0
ybaQ	-1.7	4.3	88.5	0.0	0.0	*ppsA*	1.3	11.8	53.6	0.0	0.0
ybaY	-1.1	6.8	21.6	0.0	0.0	*pqqL*	1.2	5.3	54.5	0.0	0.0
ybdZ	1.2	2.6	21.0	0.0	0.0	*preA*	-2.6	6.8	94.9	0.0	0.0
ybeD	-1.3	6.7	93.7	0.0	0.0	*preT*	-2.3	6.3	49.5	0.0	0.0
ybeR	2.0	1.4	29.9	0.0	0.0	*priB*	1.1	9.8	17.3	0.0	0.0
ybfA	1.9	4.8	34.9	0.0	0.0	*prlC*	-1.5	9.0	46.2	0.0	0.0
ybfB	1.7	1.7	20.2	0.0	0.0	*prpR*	-1.6	4.4	66.5	0.0	0.0
ybgE	1.4	7.4	24.8	0.0	0.0	*psiE*	2.5	2.6	168.8	0.0	0.0
ybgI	-1.0	6.8	23.0	0.0	0.0	*pspA*	1.6	6.4	98.0	0.0	0.0
ybgU	-3.2	6.4	88.4	0.0	0.0	*pspB*	1.9	4.4	194.2	0.0	0.0
ybjM	1.2	4.5	106.0	0.0	0.0	*pspC*	1.6	4.6	140.2	0.0	0.0
yceH	-1.0	6.2	28.3	0.0	0.0	*pspD*	1.7	3.1	61.5	0.0	0.0
ycfZ	1.0	5.1	27.2	0.0	0.0	*pspE*	-1.2	7.6	57.7	0.0	0.0
ycgZ	-2.7	3.5	89.5	0.0	0.0	*pspG*	2.4	2.3	88.2	0.0	0.0
ydcI	-2.8	6.9	89.3	0.0	0.0	*pssA*	-1.4	8.9	114.5	0.0	0.0
yddA	3.6	4.7	152.2	0.0	0.0	*pstA*	1.4	5.3	63.7	0.0	0.0
yddB	2.7	5.2	146.9	0.0	0.0	*pstC*	1.7	5.6	133.9	0.0	0.0
yddK	2.4	0.5	16.7	0.0	0.0	*pstS*	2.0	6.6	69.4	0.0	0.0
ydeR	1.9	0.7	16.9	0.0	0.0	*psuG*	-1.7	3.9	69.9	0.0	0.0
ydfD	-1.7	0.0	20.0	0.0	0.0	*psuK*	-1.5	3.5	119.0	0.0	0.0
ydfJ	1.2	2.9	21.0	0.0	0.0	*purL*	1.1	7.2	32.9	0.0	0.0
ydfZ	1.3	2.2	24.3	0.0	0.0	*purM*	1.1	5.6	16.8	0.0	0.0
ydhP	1.7	4.8	112.1	0.0	0.0	*purR*	1.2	7.2	25.0	0.0	0.0
ydiE	1.1	4.6	57.5	0.0	0.0	*putP*	-1.7	7.9	16.5	0.0	0.0
yeaC	-1.2	6.7	50.4	0.0	0.0	*pykA*	-1.3	9.1	109.6	0.0	0.0
yeaD	-1.1	7.6	38.4	0.0	0.0	*qorA*	-1.0	5.8	24.0	0.0	0.0
yebB	1.4	4.4	42.5	0.0	0.0	*rbsB*	-1.1	12.4	65.4	0.0	0.0
yebQ	1.3	3.8	37.0	0.0	0.0	*rcnB*	1.0	7.0	44.9	0.0	0.0
yebV	1.2	4.4	61.7	0.0	0.0	*recA*	2.8	10.3	297.1	0.0	0.0
yecN	1.0	7.6	59.9	0.0	0.0	*recN*	2.6	8.2	436.2	0.0	0.0
yedE	2.3	7.4	166.5	0.0	0.0	*recX*	2.5	4.0	178.6	0.0	0.0
yedF	2.3	5.8	214.8	0.0	0.0	*rem*	1.3	2.4	31.2	0.0	0.0
yeeW	1.0	2.2	21.4	0.0	0.0	*rhaR*	-1.2	5.3	62.4	0.0	0.0
yegJ	1.3	1.2	17.1	0.0	0.0	*rhlE*	1.8	5.9	57.7	0.0	0.0
yehF	1.4	3.4	45.0	0.0	0.0	*rhsC*	1.4	5.6	173.9	0.0	0.0
yehL	2.0	1.6	39.6	0.0	0.0	*ribB*	-2.6	9.2	211.6	0.0	0.0
yfaA	1.4	4.6	57.1	0.0	0.0	*rihA*	-1.8	9.0	126.8	0.0	0.0
yfaQ	1.4	4.1	43.9	0.0	0.0	*rihC*	-1.9	7.3	162.2	0.0	0.0

yfaT	1.5	2.5	21.0	0.0	0.0
yfbK	1.2	3.6	70.5	0.0	0.0
yfbL	2.0	1.2	17.7	0.0	0.0
yfbM	1.8	2.6	95.6	0.0	0.0
yfbN	1.5	1.9	36.5	0.0	0.0
yfbU	-1.3	7.2	32.5	0.0	0.0
yfgI	1.1	3.3	41.2	0.0	0.0
yfiM	-1.1	5.4	144.5	0.0	0.0
ygaM	-1.6	5.7	123.5	0.0	0.0
ygbJ	-1.4	4.1	61.3	0.0	0.0
ygbL	-1.0	3.5	19.1	0.0	0.0
yghG	-1.3	3.9	30.0	0.0	0.0
yghJ	-1.6	7.8	133.6	0.0	0.0
yghO	-1.5	3.9	34.5	0.0	0.0
yghR	1.1	1.8	19.5	0.0	0.0
ygiM	-1.6	7.0	151.6	0.0	0.0
ygiQ	1.3	7.1	29.5	0.0	0.0
ygjH	-1.2	2.5	29.4	0.0	0.0
ygjI	-1.6	2.7	109.7	0.0	0.0
yhbE	1.4	7.7	19.3	0.0	0.0
yhbW	-1.2	5.2	35.4	0.0	0.0
yhcA	1.7	2.0	23.4	0.0	0.0
yhdJ	1.6	4.5	50.7	0.0	0.0
yheV	-1.7	4.6	33.9	0.0	0.0
yhfL	1.3	3.1	47.6	0.0	0.0
yhiD	-1.0	1.7	16.5	0.0	0.0
yhjC	-1.1	4.7	17.6	0.0	0.0
yiaK	-1.3	3.8	49.0	0.0	0.0
yiaL	-1.5	2.5	37.0	0.0	0.0
yiaM	-2.4	1.1	42.2	0.0	0.0
yiaN	-1.2	2.4	59.3	0.0	0.0
yiaO	-1.1	2.6	43.9	0.0	0.0
yibF	-1.1	5.6	50.8	0.0	0.0
yigB	-1.0	5.4	86.9	0.0	0.0
yigI	-2.5	5.5	165.4	0.0	0.0
yihM	-1.1	5.2	85.7	0.0	0.0
yihN	-1.6	4.8	139.6	0.0	0.0
yiiS	-1.0	6.3	27.1	0.0	0.0
yjcH	-2.4	4.0	34.0	0.0	0.0
yjfI	1.1	3.1	25.1	0.0	0.0
yjfK	1.4	3.3	28.1	0.0	0.0
yjfL	1.4	1.8	17.4	0.0	0.0
yjfM	1.1	3.0	26.6	0.0	0.0
yjiH	1.4	2.7	47.5	0.0	0.0
yjjU	-1.1	5.1	98.0	0.0	0.0
ykfG	1.5	3.0	42.3	0.0	0.0
ykfH	1.1	1.9	19.9	0.0	0.0
ykfI	1.6	4.2	69.0	0.0	0.0
ykfM	2.0	2.1	85.9	0.0	0.0
rluB	1.6	7.7	34.3	0.0	0.0
rmf	1.5	5.2	20.0	0.0	0.0
rpiB	-2.2	4.3	48.6	0.0	0.0
rplI	1.1	10.9	20.1	0.0	0.0
rplT	1.0	11.6	45.6	0.0	0.0
rpmI	1.0	10.7	50.4	0.0	0.0
rpsF	1.1	10.4	17.0	0.0	0.0
rpsJ	1.1	11.2	16.0	0.0	0.0
rpsR	1.1	9.7	18.1	0.0	0.0
rsmJ	-1.3	6.7	60.7	0.0	0.0
rsxC	1.1	7.5	24.9	0.0	0.0
rsxD	1.4	6.5	55.6	0.0	0.0
rsxE	1.1	5.5	38.5	0.0	0.0
rsxG	1.4	5.7	44.8	0.0	0.0
rutE	1.2	1.4	29.7	0.0	0.0
ruvA	1.3	6.6	135.5	0.0	0.0
ruvB	1.0	7.2	122.9	0.0	0.0
rydB	2.5	0.8	42.5	0.0	0.0
ryfA	1.4	6.0	123.7	0.0	0.0
ryfD	-2.2	7.1	57.2	0.0	0.0
sbmC	2.0	6.4	165.2	0.0	0.0
sdhA	-1.4	11.4	22.8	0.0	0.0
sdhB	-1.4	10.0	18.3	0.0	0.0
sdhD	-1.3	9.3	38.5	0.0	0.0
secB	-1.4	9.7	63.7	0.0	0.0
setC	1.0	1.9	17.3	0.0	0.0
sfmC	2.7	1.6	32.4	0.0	0.0
sfmD	1.6	3.3	122.0	0.0	0.0
sgbE	-1.1	2.5	15.6	0.0	0.0
sgbH	-2.1	1.4	33.0	0.0	0.0
sgbU	-2.0	2.1	119.7	0.0	0.0
sodA	-1.0	9.3	25.2	0.0	0.0
soxS	3.2	5.8	204.2	0.0	0.0
spf	-1.0	4.0	54.3	0.0	0.0
spy	1.8	5.5	212.6	0.0	0.0
sraG	-1.1	8.0	58.4	0.0	0.0
srlR	-1.0	6.7	130.9	0.0	0.0
sseA	-1.2	8.1	61.7	0.0	0.0
sstT	-1.1	6.9	43.6	0.0	0.0
ssuC	1.3	1.3	19.6	0.0	0.0
sthA	-1.7	9.0	33.9	0.0	0.0
sucA	-1.6	12.3	25.5	0.0	0.0
sucB	-1.2	12.1	25.8	0.0	0.0
sucC	-1.1	11.8	19.7	0.0	0.0
suhB	2.6	8.2	39.4	0.0	0.0
sulA	4.1	8.4	813.4	0.0	0.0
talB	-1.0	10.3	47.2	0.0	0.0
tdcA	-2.6	8.4	289.3	0.0	0.0
tdcB	-3.6	6.4	518.5	0.0	0.0

ykgH	-1.1	3.4	19.0	0.0	0.0
ymfA	2.1	4.6	62.6	0.0	0.0
ymgA	-2.7	3.8	112.5	0.0	0.0
ymgC	-2.9	2.2	99.7	0.0	0.0
ymgG	-1.1	5.6	23.0	0.0	0.0
yncJ	1.1	3.4	22.8	0.0	0.0
ynhF	1.8	3.2	82.4	0.0	0.0
ynjE	1.2	5.4	98.3	0.0	0.0
yoaL	1.0	4.7	65.3	0.0	0.0
ypjD	-1.3	6.6	67.8	0.0	0.0
yqaB	-1.1	7.1	100.0	0.0	0.0
yqeB	-1.3	6.3	24.6	0.0	0.0
yqhD	-1.1	5.8	26.2	0.0	0.0
yqhH	1.2	2.8	22.9	0.0	0.0
yqiJ	1.2	3.5	35.8	0.0	0.0
yqiK	1.2	4.9	31.8	0.0	0.0
yraQ	1.1	6.1	54.2	0.0	0.0
yrbN	2.2	5.6	37.0	0.0	0.0
ytfE	-1.1	4.9	26.6	0.0	0.0
ytfQ	-1.7	7.2	43.8	0.0	0.0
ytfR	-1.3	5.9	29.8	0.0	0.0
ytfT	-1.1	4.6	23.7	0.0	0.0
ytjA	-1.0	6.1	21.2	0.0	0.0

*pnp^{+}, C-1a; Δ*pnp*, C-5691; *hPNP*, C-6001

tdcC	-1.7	6.4	55.1	0.0	0.0
tdcD	-2.0	5.8	37.4	0.0	0.0
tdcE	-1.6	6.5	20.6	0.0	0.0
tdcF	-1.6	3.7	22.0	0.0	0.0
tdh	-1.5	9.0	89.0	0.0	0.0
thiM	1.2	5.1	39.5	0.0	0.0
tisB	4.5	8.1	575.8	0.0	0.0
tnaA	-5.6	12.6	295.8	0.0	0.0
tnaB	-6.0	9.4	250.3	0.0	0.0
tnaC	-6.1	9.5	207.3	0.0	0.0
tomB	1.2	6.6	114.8	0.0	0.0
torS	1.1	4.8	70.3	0.0	0.0
torY	1.2	2.7	24.8	0.0	0.0
tpiA	-1.3	8.9	71.0	0.0	0.0
treA	-1.5	5.9	112.2	0.0	0.0
treB	-1.1	12.0	42.6	0.0	0.0
trmL	-1.2	6.6	31.2	0.0	0.0
trpT	1.0	5.2	34.5	0.0	0.0
tsgA	2.3	5.7	108.8	0.0	0.0
tsx	-2.5	11.1	307.9	0.0	0.0
ttdB	1.1	1.3	20.8	0.0	0.0
tyrB	-1.6	6.9	80.3	0.0	0.0
udp	-1.3	11.8	47.2	0.0	0.0
ugpA	-2.9	3.5	41.5	0.0	0.0
ugpB	-2.9	6.1	54.8	0.0	0.0
ugpC	-1.8	5.5	28.8	0.0	0.0
ugpE	-2.7	3.8	43.6	0.0	0.0
ugpQ	-1.1	5.9	49.3	0.0	0.0
uhpC	1.0	4.6	51.1	0.0	0.0
uhpT	3.4	6.2	412.1	0.0	0.0
uidA	-1.9	4.1	120.4	0.0	0.0
uidC	1.3	2.4	28.6	0.0	0.0
ulaB	-1.3	2.9	26.3	0.0	0.0
ulaC	-1.4	2.5	19.5	0.0	0.0
ulaD	-1.2	3.3	18.9	0.0	0.0
ulaF	-1.0	3.5	59.8	0.0	0.0
umuC	3.0	6.1	323.9	0.0	0.0
umuD	4.2	5.2	710.8	0.0	0.0
ung	-1.3	6.4	218.6	0.0	0.0
uspG	1.4	9.1	46.7	0.0	0.0
uvrA	1.2	8.4	118.8	0.0	0.0
uxaA	-1.5	6.3	55.4	0.0	0.0
uxaB	1.3	4.9	112.6	0.0	0.0
uxaC	-1.9	6.5	77.5	0.0	0.0
uxuA	-2.0	7.2	112.1	0.0	0.0
uxuB	-1.8	7.1	138.0	0.0	0.0
valT	1.5	8.6	15.5	0.0	0.0
ves	1.7	3.5	60.9	0.0	0.0
wcaA	1.7	2.8	40.4	0.0	0.0

wcaD	2.1	1.0	22.9	0.0	0.0
wcaE	2.1	0.0	33.7	0.0	0.0
wcaI	1.6	1.4	25.6	0.0	0.0
wza	1.4	1.5	39.7	0.0	0.0
wzxC	1.8	1.1	38.8	0.0	0.0
xapB	1.2	4.5	101.5	0.0	0.0
xapR	1.1	4.4	40.5	0.0	0.0
xdhA	-2.0	6.3	126.0	0.0	0.0
xdhB	-2.1	4.0	51.1	0.0	0.0
xdhC	-2.2	4.1	78.7	0.0	0.0
xerC	-1.1	6.6	158.1	0.0	0.0
xylF	-1.6	3.7	74.4	0.0	0.0
yadK	1.6	2.1	43.6	0.0	0.0
yadL	1.2	2.7	38.9	0.0	0.0
yadM	1.2	2.7	74.5	0.0	0.0
yadN	1.7	1.3	23.5	0.0	0.0
yadV	1.4	2.5	42.6	0.0	0.0
yafP	1.9	3.6	183.7	0.0	0.0
yafT	1.1	3.8	24.1	0.0	0.0
yafW	1.5	3.3	61.2	0.0	0.0
yagE	-2.2	5.4	49.5	0.0	0.0
yagF	-1.4	5.5	16.0	0.0	0.0
yagU	-1.6	6.4	59.6	0.0	0.0
yaiO	1.8	3.3	93.9	0.0	0.0
yaiP	1.3	2.0	23.2	0.0	0.0
yaiY	1.2	3.8	97.7	0.0	0.0
yaiZ	-1.2	5.8	56.8	0.0	0.0
yajI	1.1	4.1	92.9	0.0	0.0
ybaQ	-1.4	4.3	59.3	0.0	0.0
ybdD	-1.3	4.6	22.1	0.0	0.0
ybeR	2.6	1.4	52.5	0.0	0.0
ybeU	1.0	2.2	23.8	0.0	0.0
ybfA	2.5	4.8	57.8	0.0	0.0
ybgS	1.5	4.2	142.3	0.0	0.0
ybgU	-2.0	6.4	37.3	0.0	0.0
ybhH	3.5	0.4	29.1	0.0	0.0
ybhI	1.6	2.1	29.0	0.0	0.0
ybhM	1.1	3.6	23.0	0.0	0.0
ybjM	1.2	4.5	103.6	0.0	0.0
ycaD	1.4	6.0	19.8	0.0	0.0
ycdU	2.1	2.1	32.6	0.0	0.0
ycfJ	2.6	4.9	450.3	0.0	0.0
yciF	1.9	0.3	35.2	0.0	0.0
ydcH	-1.5	6.3	32.9	0.0	0.0
ydcI	-2.1	6.9	53.0	0.0	0.0
ydcJ	-1.3	6.1	108.7	0.0	0.0
ydcL	-1.1	6.2	74.6	0.0	0.0
ydcS	-2.2	5.9	85.8	0.0	0.0
ydcT	-2.3	4.7	118.8	0.0	0.0

ydcU	-1.6	4.5	85.2	0.0	0.0
ydcV	-1.9	4.2	72.3	0.0	0.0
yddA	2.0	4.7	49.8	0.0	0.0
yddB	1.4	5.2	40.4	0.0	0.0
yddH	-1.1	3.9	21.3	0.0	0.0
yddK	3.7	0.5	53.9	0.0	0.0
ydeA	1.3	3.9	63.5	0.0	0.0
ydeO	2.3	0.3	22.3	0.0	0.0
ydeP	1.2	3.9	67.0	0.0	0.0
ydeQ	2.8	0.9	53.4	0.0	0.0
ydeR	3.1	0.7	54.7	0.0	0.0
ydeS	2.6	0.9	31.8	0.0	0.0
ydeT	3.2	2.3	167.3	0.0	0.0
ydfC	1.3	3.0	80.3	0.0	0.0
ydfI	1.2	3.1	32.1	0.0	0.0
ydfJ	1.8	2.9	47.7	0.0	0.0
ydfN	1.6	1.7	20.5	0.0	0.0
ydfO	1.6	1.8	16.4	0.0	0.0
ydfU	1.0	2.2	35.3	0.0	0.0
ydfZ	1.0	2.2	16.2	0.0	0.0
ydgI	2.0	5.6	37.6	0.0	0.0
ydgK	1.3	6.3	18.5	0.0	0.0
ydhF	-1.3	6.6	55.6	0.0	0.0
ydhP	2.0	4.8	163.3	0.0	0.0
ydiH	1.4	3.0	33.7	0.0	0.0
ydiM	1.3	1.5	21.4	0.0	0.0
ydiN	2.2	0.9	52.9	0.0	0.0
ydjH	1.0	1.8	16.6	0.0	0.0
ydjM	2.0	4.4	268.1	0.0	0.0
yeaD	-1.6	7.6	72.8	0.0	0.0
yeaY	1.0	7.0	21.4	0.0	0.0
yebB	1.4	4.4	41.8	0.0	0.0
yebE	1.1	5.1	107.3	0.0	0.0
yebF	2.3	7.7	203.2	0.0	0.0
yebG	3.7	7.7	822.8	0.0	0.0
yebO	1.4	7.8	24.6	0.0	0.0
yebQ	1.6	3.8	59.0	0.0	0.0
yecN	1.2	7.6	83.6	0.0	0.0
yedE	1.7	7.4	89.4	0.0	0.0
yedF	1.5	5.8	99.1	0.0	0.0
yedR	1.4	2.1	43.7	0.0	0.0
yeeA	1.5	5.7	179.8	0.0	0.0
yeeS	1.3	3.2	41.3	0.0	0.0
yeeW	1.2	2.2	32.0	0.0	0.0
yegJ	1.7	1.2	32.3	0.0	0.0
yegL	1.1	2.3	28.6	0.0	0.0
yegQ	1.7	6.4	31.1	0.0	0.0
yegR	1.4	2.5	41.5	0.0	0.0
yegT	-1.3	3.7	34.5	0.0	0.0

yehA	1.5	3.3	42.0	0.0	0.0
yehB	1.1	2.2	20.2	0.0	0.0
yehF	2.2	3.4	106.3	0.0	0.0
yehL	2.1	1.6	42.5	0.0	0.0
yfaA	1.2	4.6	46.7	0.0	0.0
yfaQ	1.5	4.1	48.5	0.0	0.0
yfaT	1.4	2.5	17.0	0.0	0.0
yfbK	1.1	3.6	57.8	0.0	0.0
yfbL	2.1	1.2	19.9	0.0	0.0
yfbN	1.1	1.9	21.2	0.0	0.0
yfbU	-1.1	7.2	25.3	0.0	0.0
yfcQ	1.2	2.3	35.2	0.0	0.0
yfcV	1.8	0.6	37.9	0.0	0.0
yfdV	2.0	1.1	45.9	0.0	0.0
yfeK	1.8	4.8	78.6	0.0	0.0
yfeS	1.1	6.4	57.6	0.0	0.0
yfeW	-1.5	5.7	138.2	0.0	0.0
yfgI	1.3	3.3	65.5	0.0	0.0
yfhH	1.2	5.6	68.5	0.0	0.0
yfhR	1.1	3.6	19.0	0.0	0.0
yfiM	-1.3	5.4	172.6	0.0	0.0
yfjD	-1.2	7.5	82.9	0.0	0.0
yfjQ	1.2	2.1	21.6	0.0	0.0
ygaM	-1.3	5.7	80.9	0.0	0.0
ygbJ	-1.9	4.1	110.5	0.0	0.0
ygbL	-1.4	3.5	37.2	0.0	0.0
ygcP	-1.0	4.6	61.8	0.0	0.0
ygeV	-2.7	8.2	110.2	0.0	0.0
yghG	-1.1	3.9	23.5	0.0	0.0
yghJ	-1.9	7.8	182.2	0.0	0.0
yghO	-1.1	3.9	19.7	0.0	0.0
yghR	1.1	1.8	20.5	0.0	0.0
ygiQ	1.3	7.1	30.3	0.0	0.0
ygjH	-1.9	2.5	71.7	0.0	0.0
ygjI	-1.8	2.7	139.1	0.0	0.0
ygjR	-1.9	6.7	45.7	0.0	0.0
yhbE	1.4	7.7	21.7	0.0	0.0
yhcA	2.0	2.0	33.1	0.0	0.0
yhcH	-1.8	7.8	72.8	0.0	0.0
yhdH	-1.1	6.6	65.5	0.0	0.0
yhdJ	1.7	4.5	59.5	0.0	0.0
yhdV	1.2	1.9	24.2	0.0	0.0
yheV	-1.7	4.6	33.3	0.0	0.0
yhfA	-1.3	7.1	31.7	0.0	0.0
yhfL	1.6	3.1	67.8	0.0	0.0
yhhQ	1.1	3.9	27.5	0.0	0.0
yhhX	-1.1	6.9	29.8	0.0	0.0
yhjV	-1.3	7.0	68.4	0.0	0.0
yiaK	-2.0	3.8	101.3	0.0	0.0

yiaL	-2.6	2.5	90.5	0.0	0.0
yiaM	-1.7	1.1	25.5	0.0	0.0
yiaN	-1.1	2.4	45.5	0.0	0.0
yiaO	-2.2	2.6	138.9	0.0	0.0
yicS	1.0	2.8	16.2	0.0	0.0
yidE	-1.2	7.4	37.1	0.0	0.0
yidP	-1.1	3.6	19.7	0.0	0.0
yigB	-1.2	5.4	117.3	0.0	0.0
yihM	-1.9	5.2	246.5	0.0	0.0
yihN	-2.3	4.8	261.8	0.0	0.0
yihT	-1.1	3.3	25.7	0.0	0.0
yihU	-1.6	2.5	43.1	0.0	0.0
yihW	-1.0	6.5	127.2	0.0	0.0
yihY	-1.0	6.3	85.9	0.0	0.0
yiiX	1.1	5.3	26.7	0.0	0.0
yjbE	2.0	1.6	51.5	0.0	0.0
yjcB	1.7	4.4	70.4	0.0	0.0
yjcH	-2.9	4.0	47.5	0.0	0.0
yjfI	1.1	3.1	24.3	0.0	0.0
yjfJ	1.2	3.7	80.4	0.0	0.0
yjfK	1.5	3.3	28.8	0.0	0.0
yjfL	1.4	1.8	18.5	0.0	0.0
yjfM	1.0	3.0	22.6	0.0	0.0
yjgR	-1.6	6.9	107.3	0.0	0.0
yjiA	-1.0	5.2	36.9	0.0	0.0
yjiH	1.6	2.7	61.5	0.0	0.0
yjjQ	2.0	0.9	24.6	0.0	0.0
ykfG	1.4	3.0	38.9	0.0	0.0
ykfH	1.2	1.9	24.0	0.0	0.0
ykfI	1.5	4.2	62.9	0.0	0.0
ykfM	2.0	2.1	82.7	0.0	0.0
ykgE	-1.7	7.6	71.1	0.0	0.0
ykgF	-1.7	8.4	97.5	0.0	0.0
ykgG	-1.6	7.3	113.9	0.0	0.0
ykgH	-1.5	3.4	32.9	0.0	0.0
yliM	1.7	6.5	28.9	0.0	0.0
ymdG	1.4	1.1	21.5	0.0	0.0
ymfA	1.9	4.6	51.7	0.0	0.0
ymgC	-1.1	2.2	22.1	0.0	0.0
ymgD	1.9	6.2	62.5	0.0	0.0
ymgG	1.9	5.6	69.5	0.0	0.0
ymgI	2.0	3.6	119.6	0.0	0.0
yncJ	1.2	3.4	27.2	0.0	0.0
ynfQ	3.1	1.6	43.1	0.0	0.0
ynhF	2.0	3.2	102.7	0.0	0.0
yoaL	1.0	4.7	63.5	0.0	0.0
yobB	1.2	6.7	122.2	0.0	0.0
yobF	-1.1	7.4	36.4	0.0	0.0
yohJ	1.5	3.0	46.7	0.0	0.0

ypdK	2.1	2.3	25.4	0.0	0.0
ypeC	1.6	5.0	89.8	0.0	0.0
ypfG	1.3	5.0	58.5	0.0	0.0
ypjD	-1.2	6.6	61.6	0.0	0.0
yqaA	-1.1	7.3	147.4	0.0	0.0
yqaB	-1.0	7.1	94.4	0.0	0.0
yqeB	-2.7	6.3	88.5	0.0	0.0
yqeC	-2.9	5.8	59.1	0.0	0.0
yqeF	-1.3	9.2	57.4	0.0	0.0
yqgD	1.4	4.2	28.1	0.0	0.0
yqhH	1.1	2.8	21.3	0.0	0.0
yqiJ	1.0	3.5	25.1	0.0	0.0
yqiK	1.1	4.9	22.9	0.0	0.0
yraQ	1.6	6.1	114.3	0.0	0.0
yrbN	2.1	5.6	34.2	0.0	0.0
ysaB	1.0	2.8	23.9	0.0	0.0
ytfE	-1.2	4.9	30.9	0.0	0.0
ytfQ	-3.2	7.2	135.6	0.0	0.0
ytfR	-2.6	5.9	112.3	0.0	0.0
ytfT	-2.5	4.6	101.9	0.0	0.0
yzgL	-1.6	7.5	41.2	0.0	0.0

*pnp^{+}, C-1a; Δ*pnp*, C-5691; *hPNP*, C-6001
