# Supplementary material for: Human PNPase causes RNA stabilization and accumulation of R-loops in the Escherichia coli model system
Source: Sci Rep. 2023 Jul 21;13:11771. doi: 10.1038/s41598-023-38924-x (PMC10362022; doi:10.1038/s41598-023-38924-x)
Supplement: Supplementary file 3 — Supplementary Table S2. [file 41598_2023_38924_MOESM3_ESM.pdf]

Table S2. Global RNA stability

Gene[a]	*pnp* [+b]	Δ*pnp* [b]	*PNPT1* $_{Ec}$ [b]	Δ*pnp* *vs*. *pnp* [+c]	*PNPT1* $_{Ec}$ *vs. pnp* [+d]	Δ*pnp* *vs*. *PNPT1* $_{Ec}$ [e]
aaeA	4	3	3	-1	-1	0
aaeB	4	3	2	-1	-2	1
aaeR	1	1	1	0	0	0
aaeX	4	4	3	0	-1	1
aas	4	4	4	0	0	0
aat	4	4	4	0	0	0
abgA	2	2	2	0	0	0
abgB	2	3	2	1	0	1
abgR	4	4	4	0	0	0
abgT	4	4	4	0	0	0
abrB	3	3	2	0	-1	1
accA	1	1	1	0	0	0
accB	1	1	1	0	0	0
accC	1	1	1	0	0	0
accD	3	3	3	0	0	0
aceA	1	1	1	0	0	0
aceB	1	3	3	2	2	0
aceE	1	1	2	0	1	-1
aceF	1	1	1	0	0	0
aceK	2	3	2	1	0	1
ackA	2	3	3	1	1	0
acnA	1	1	1	0	0	0
acnB	1	1	1	0	0	0
acpH	4	4	3	0	-1	1
acpP	1	1	1	0	0	0
acpS	1	1	1	0	0	0
acpT	4	4	4	0	0	0
acrA	2	3	2	1	0	1
acrB	1	1	1	0	0	0
acrD	4	4	3	0	-1	1
acrE	4	3	2	-1	-2	1
acrF	4	3	3	-1	-1	0
acrR	4	4	4	0	0	0
acrZ	4	4	3	0	-1	1
acs	1	1	1	0	0	0
actP	1	1	1	0	0	0
ada	4	4	3	0	-1	1
add	2	3	2	1	0	1
adeD	4	4	3	0	-1	1
adeP	4	4	3	0	-1	1
adeQ	4	3	2	-1	-2	1
adhE	3	3	2	0	-1	1
adhP	1	1	1	0	0	0
adiA	4	4	4	0	0	0
adiC	4	3	3	-1	-1	0
adiY	1	2	2	1	1	0
adk	1	1	1	0	0	0
aegA	3	4	3	1	0	1

aer	3	3	3	0	0	0
aes	3	3	3	0	0	0
afuC	4	4	3	0	-1	1
agaB	2	1	1	-1	-1	0
agaC	2	2	2	0	0	0
agaD	3	2	2	-1	-1	0
agaI	3	3	2	0	-1	1
agaR	4	4	4	0	0	0
agaS	3	3	3	0	0	0
agaV	1	1	1	0	0	0
agp	1	1	1	0	0	0
agrA	2	2	2	0	0	0
agrB	4	4	4	0	0	0
ahpC	1	1	1	0	0	0
ahpF	1	2	2	1	1	0
ahr	1	1	1	0	0	0
aidB	2	2	1	0	-1	1
ais	4	4	3	0	-1	1
alaA	1	2	1	1	0	1
alaC	4	4	3	0	-1	1
alaE	4	4	3	0	-1	1
alaS	3	3	3	0	0	0
alaT	4	4	4	0	0	0
alaU	4	4	4	0	0	0
alaV	4	4	4	0	0	0
alaW	2	3	2	1	0	1
alaX	2	3	2	1	0	1
aldA	1	3	2	2	1	1
aldB	1	2	1	1	0	1
alkA	3	3	2	0	-1	1
alkB	4	4	3	0	-1	1
allA	4	4	4	0	0	0
allB	1	1	1	0	0	0
allC	2	2	2	0	0	0
allD	2	4	3	2	1	1
allE	1	1	1	0	0	0
allR	1	1	1	0	0	0
allS	4	4	4	0	0	0
alr	4	4	3	0	-1	1
alsA	3	3	3	0	0	0
alsB	3	3	3	0	0	0
alsC	3	3	2	0	-1	1
alsE	2	2	1	0	-1	1
alsK	3	3	2	0	-1	1
alsR	3	3	3	0	0	0
alx	4	4	3	0	-1	1
amiA	4	4	4	0	0	0
amiB	3	4	3	1	0	1
amiC	4	4	4	0	0	0
amiD	4	4	3	0	-1	1
amn	2	3	2	1	0	1

ampC	3	4	3	1	0	1
ampD	2	2	1	0	-1	1
ampE	1	2	1	1	0	1
ampG	4	4	4	0	0	0
ampH	4	4	4	0	0	0
amtB	3	2	2	-1	-1	0
amyA	1	1	1	0	0	0
anmK	3	3	2	0	-1	1
ansA	3	4	3	1	0	1
ansB	1	1	1	0	0	0
ansP	2	1	1	-1	-1	0
apaG	4	4	3	0	-1	1
apaH	3	3	3	0	0	0
aphA	3	2	2	-1	-1	0
appA	1	1	1	0	0	0
appB	1	1	1	0	0	0
appC	1	1	1	0	0	0
appX	1	1	1	0	0	0
apt	1	2	2	1	1	0
aqpZ	3	3	2	0	-1	1
araA	1	3	2	2	1	1
araB	3	3	2	0	-1	1
araC	4	4	3	0	-1	1
araD	1	2	1	1	0	1
araE	3	3	2	0	-1	1
araF	1	1	1	0	0	0
araG	1	2	2	1	1	0
araH	1	2	1	1	0	1
araJ	3	3	2	0	-1	1
arcA	2	2	1	0	-1	1
arcB	4	4	4	0	0	0
arcZ	2	3	3	1	1	0
arfA	2	2	2	0	0	0
arfB	4	4	3	0	-1	1
argA	1	1	1	0	0	0
argB	2	3	2	1	0	1
argC	4	4	3	0	-1	1
argD	4	4	4	0	0	0
argE	1	3	2	2	1	1
argF	3	4	3	1	0	1
argG	2	3	2	1	0	1
argH	1	2	1	1	0	1
argI	3	3	2	0	-1	1
argK	4	3	3	-1	-1	0
argO	4	3	3	-1	-1	0
argP	4	4	3	0	-1	1
argQ	4	4	4	0	0	0
argR	3	2	2	-1	-1	0
argS	3	3	3	0	0	0
argT	3	4	3	1	0	1
argU	4	4	4	0	0	0

argV	4	4	4	0	0	0
argW	4	4	4	0	0	0
argX	4	4	4	0	0	0
argY	4	4	4	0	0	0
argZ	4	4	4	0	0	0
ariR	1	2	1	1	0	1
arnA	2	4	3	2	1	1
arnB	3	4	4	1	1	0
arnC	2	4	4	2	2	0
arnD	3	4	3	1	0	1
arnE	3	2	2	-1	-1	0
arnF	3	3	3	0	0	0
arnT	3	3	3	0	0	0
aroA	1	2	2	1	1	0
aroB	4	4	3	0	-1	1
aroC	3	4	3	1	0	1
aroD	1	1	1	0	0	0
aroE	3	3	2	0	-1	1
aroF	3	4	3	1	0	1
aroG	2	3	2	1	0	1
aroH	2	3	3	1	1	0
aroK	4	4	4	0	0	0
aroL	4	4	3	0	-1	1
aroM	4	4	3	0	-1	1
aroP	3	3	3	0	0	0
arpA	4	3	3	-1	-1	0
arrS	4	4	4	0	0	0
arsB	4	4	3	0	-1	1
arsC	3	3	3	0	0	0
arsR	4	4	4	0	0	0
artI	2	3	2	1	0	1
artJ	2	3	3	1	1	0
artM	4	4	3	0	-1	1
artP	4	4	4	0	0	0
artQ	4	3	3	-1	-1	0
ascB	3	3	2	0	-1	1
ascF	4	4	3	0	-1	1
ascG	4	4	3	0	-1	1
asd	1	1	1	0	0	0
aslA	3	3	2	0	-1	1
aslB	4	4	3	0	-1	1
asmA	4	4	3	0	-1	1
asnA	3	4	2	1	-1	2
asnB	3	3	2	0	-1	1
asnC	4	3	3	-1	-1	0
asnS	1	2	2	1	1	0
asnT	4	4	4	0	0	0
asnU	3	4	4	1	1	0
asnV	3	4	4	1	1	0
asnW	4	4	4	0	0	0
aspA	1	1	1	0	0	0

aspC	1	1	1	0	0	0
aspS	2	2	2	0	0	0
aspT	4	4	3	0	-1	1
aspU	2	4	3	2	1	1
aspV	3	3	3	0	0	0
asr	2	2	1	0	-1	1
astA	1	2	1	1	0	1
astB	1	2	1	1	0	1
astC	1	2	1	1	0	1
astD	1	2	1	1	0	1
astE	1	2	1	1	0	1
atl	4	4	3	0	-1	1
atoA	1	1	1	0	0	0
atoB	1	1	1	0	0	0
atoC	3	4	3	1	0	1
atoD	1	1	1	0	0	0
atoE	1	1	1	0	0	0
atoS	4	4	4	0	0	0
atpA	1	1	1	0	0	0
atpB	4	4	4	0	0	0
atpC	1	1	1	0	0	0
atpD	1	1	1	0	0	0
atpE	1	1	1	0	0	0
atpF	1	1	1	0	0	0
atpG	1	1	1	0	0	0
atpH	1	1	1	0	0	0
atpI	4	4	4	0	0	0
avtA	4	4	4	0	0	0
azoR	1	2	2	1	1	0
azuC	4	4	3	0	-1	1
bacA	3	3	2	0	-1	1
baeR	1	1	1	0	0	0
baeS	1	1	1	0	0	0
bamA	2	2	2	0	0	0
bamB	1	1	1	0	0	0
bamC	2	2	2	0	0	0
bamD	1	2	1	1	0	1
bamE	4	4	3	0	-1	1
barA	4	4	4	0	0	0
basR	4	3	3	-1	-1	0
basS	4	3	3	-1	-1	0
bax	4	4	4	0	0	0
bcp	1	1	1	0	0	0
bcr	4	4	3	0	-1	1
bcsA	4	3	3	-1	-1	0
bcsB	1	2	2	1	1	0
bcsC	1	1	1	0	0	0
bcsE	4	4	4	0	0	0
bcsF	4	4	3	0	-1	1
bcsG	3	3	3	0	0	0
bcsZ	1	1	1	0	0	0

bdcA	3	3	2	0	-1	1
bdcR	4	3	2	-1	-2	1
bdm	2	3	3	1	1	0
bepA	3	4	3	1	0	1
betA	3	3	2	0	-1	1
betB	3	3	2	0	-1	1
betI	4	4	2	0	-2	2
betT	4	4	3	0	-1	1
bfd	4	4	4	0	0	0
bfr	1	1	1	0	0	0
bglA	2	2	1	0	-1	1
bglB	1	1	1	0	0	0
bglF	3	2	2	-1	-1	0
bglG	4	3	3	-1	-1	0
bglH	3	2	2	-1	-1	0
bglJ	4	4	4	0	0	0
bglX	2	3	2	1	0	1
bhsA	3	4	2	1	-1	2
bioA	4	4	3	0	-1	1
bioB	3	3	3	0	0	0
bioC	3	2	1	-1	-2	1
bioD	2	2	2	0	0	0
bioF	3	3	2	0	-1	1
bioH	4	3	3	-1	-1	0
bioP	4	4	3	0	-1	1
bipA	2	2	2	0	0	0
birA	4	4	4	0	0	0
bisC	4	4	3	0	-1	1
blc	4	4	3	0	-1	1
blr	4	4	3	0	-1	1
bluF	4	4	4	0	0	0
bluR	4	4	4	0	0	0
bolA	1	2	1	1	0	1
brnQ	4	4	3	0	-1	1
bsmA	4	1	1	-3	-3	0
bssR	2	1	1	-1	-1	0
bssS	3	2	1	-1	-2	1
btsR	4	4	4	0	0	0
btsS	4	4	4	0	0	0
btsT	1	1	1	0	0	0
btuB	1	2	1	1	0	1
btuC	4	4	4	0	0	0
btuD	3	3	3	0	0	0
btuE	3	3	2	0	-1	1
btuF	3	3	3	0	0	0
btuR	4	4	4	0	0	0
caiA	4	4	3	0	-1	1
caiB	3	4	3	1	0	1
caiC	1	1	1	0	0	0
caiD	1	1	1	0	0	0
caiE	1	1	1	0	0	0

caiF	2	1	1	-1	-1	0
caiT	3	3	3	0	0	0
can	3	4	3	1	0	1
carA	1	2	2	1	1	0
carB	1	1	1	0	0	0
cbeA	4	3	2	-1	-2	1
cbl	4	4	3	0	-1	1
cbpA	2	3	2	1	0	1
cbpM	2	2	1	0	-1	1
cbrA	4	4	4	0	0	0
cbrB	4	4	3	0	-1	1
cbrC	3	3	2	0	-1	1
cbtA	3	3	2	0	-1	1
cca	4	4	3	0	-1	1
ccmA	2	1	1	-1	-1	0
ccmB	3	3	3	0	0	0
ccmC	2	2	1	0	-1	1
ccmD	1	1	1	0	0	0
ccmE	1	1	1	0	0	0
ccmF	1	1	1	0	0	0
ccmG	1	1	1	0	0	0
ccmH	1	1	1	0	0	0
ccp	4	3	3	-1	-1	0
cdaR	4	4	3	0	-1	1
cdd	3	3	3	0	0	0
cdgI	3	2	1	-1	-2	1
cdh	4	4	4	0	0	0
cdsA	4	4	3	0	-1	1
cecR	4	4	4	0	0	0
cedA	4	4	4	0	0	0
cfa	2	3	2	1	0	1
chaA	4	4	3	0	-1	1
chaB	3	2	1	-1	-2	1
chaC	3	3	2	0	-1	1
chbA	3	2	1	-1	-2	1
chbB	3	3	2	0	-1	1
chbC	2	3	2	1	0	1
chbF	2	2	2	0	0	0
chbG	3	3	2	0	-1	1
chbR	3	2	1	-1	-2	1
cheA	1	1	1	0	0	0
cheB	1	1	1	0	0	0
cheR	3	3	2	0	-1	1
cheW	1	1	1	0	0	0
cheY	1	1	1	0	0	0
cheZ	1	1	1	0	0	0
chiA	3	2	1	-1	-2	1
chiP	4	4	4	0	0	0
chiQ	4	4	4	0	0	0
chiX	3	3	3	0	0	0
cho	4	4	4	0	0	0

chpB	4	3	3	-1	-1	0
chpS	4	3	2	-1	-2	1
cirA	4	4	3	0	-1	1
citC	4	4	3	0	-1	1
citD	3	2	1	-1	-2	1
citE	4	3	2	-1	-2	1
citF	3	3	2	0	-1	1
citG	4	4	3	0	-1	1
citT	4	4	3	0	-1	1
citX	3	3	3	0	0	0
clcA	4	4	3	0	-1	1
clcB	4	4	3	0	-1	1
clpA	1	2	1	1	0	1
clpB	1	2	1	1	0	1
clpP	2	2	2	0	0	0
clpS	3	4	3	1	0	1
clpX	2	2	1	0	-1	1
clsA	4	4	3	0	-1	1
clsB	4	4	3	0	-1	1
clsC	4	4	4	0	0	0
cmk	3	3	3	0	0	0
cmoA	4	4	4	0	0	0
cmoB	4	4	4	0	0	0
cmoM	4	4	4	0	0	0
cmtA	3	2	2	-1	-1	0
cmtB	4	4	3	0	-1	1
cnoX	1	2	1	1	0	1
cnu	4	4	3	0	-1	1
coaA	4	4	4	0	0	0
coaD	3	4	3	1	0	1
coaE	4	4	4	0	0	0
cobB	2	2	1	0	-1	1
cobC	3	3	2	0	-1	1
cobS	4	4	2	0	-2	2
cobT	4	4	2	0	-2	2
cobU	4	4	2	0	-2	2
codA	1	1	1	0	0	0
codB	1	1	1	0	0	0
cof	3	3	2	0	-1	1
comR	4	4	4	0	0	0
copA	2	2	1	0	-1	1
corA	4	4	4	0	0	0
cpdA	4	4	4	0	0	0
cpdB	1	1	1	0	0	0
cpoB	1	1	1	0	0	0
cpsB	2	2	1	0	-1	1
cpsG	3	3	1	0	-2	2
cpxA	4	3	3	-1	-1	0
cpxP	4	4	3	0	-1	1
cpxQ	4	4	2	0	-2	2
cpxR	4	3	2	-1	-2	1

cra	4	4	3	0	-1	1
crcB	4	4	3	0	-1	1
creA	3	3	2	0	-1	1
creB	3	3	2	0	-1	1
creC	4	4	4	0	0	0
creD	4	4	3	0	-1	1
crfC	4	4	3	0	-1	1
crp	3	3	3	0	0	0
crr	1	1	1	0	0	0
csdA	4	4	3	0	-1	1
csdE	4	4	3	0	-1	1
csgA	4	4	4	0	0	0
csgC	4	3	2	-1	-2	1
csgD	3	4	3	1	0	1
csgE	3	4	3	1	0	1
csgF	3	4	3	1	0	1
csgG	4	4	4	0	0	0
csiD	1	3	2	2	1	1
csiE	4	4	4	0	0	0
csiR	1	1	1	0	0	0
cspA	4	4	4	0	0	0
cspB	4	4	4	0	0	0
cspC	4	4	4	0	0	0
cspD	1	1	1	0	0	0
cspE	1	1	1	0	0	0
cspF	4	4	4	0	0	0
cspG	4	4	4	0	0	0
cspH	4	4	4	0	0	0
cspI	4	4	3	0	-1	1
csrA	3	4	3	1	0	1
csrB	4	2	1	-2	-3	1
csrC	3	3	3	0	0	0
csrD	4	4	4	0	0	0
cstA	3	3	2	0	-1	1
cueO	1	1	1	0	0	0
cueR	4	4	3	0	-1	1
curA	1	1	1	0	0	0
cusA	4	3	3	-1	-1	0
cusB	4	4	3	0	-1	1
cusC	4	4	3	0	-1	1
cusF	3	3	3	0	0	0
cusR	4	4	4	0	0	0
cusS	4	4	4	0	0	0
cutA	4	4	4	0	0	0
cutC	2	3	2	1	0	1
cvpA	2	2	1	0	-1	1
cvrA	4	4	3	0	-1	1
cyaA	4	4	4	0	0	0
cyaR	3	4	3	1	0	1
cyaY	3	3	3	0	0	0
cybB	3	3	3	0	0	0

cycA	3	4	3	1	0	1
cydA	1	1	1	0	0	0
cydB	1	1	1	0	0	0
cydC	4	4	3	0	-1	1
cydD	4	4	4	0	0	0
cydX	1	1	1	0	0	0
cynR	4	4	4	0	0	0
cynS	4	4	4	0	0	0
cynT	4	4	4	0	0	0
cynX	4	4	4	0	0	0
cyoA	3	3	3	0	0	0
cyoB	2	2	2	0	0	0
cyoC	1	1	1	0	0	0
cyoD	1	1	1	0	0	0
cyoE	1	1	1	0	0	0
cysA	3	3	2	0	-1	1
cysB	3	3	2	0	-1	1
cysC	4	4	3	0	-1	1
cysD	3	4	3	1	0	1
cysE	4	3	2	-1	-2	1
cysG	4	4	3	0	-1	1
cysH	1	3	2	2	1	1
cysI	1	2	2	1	1	0
cysJ	3	4	3	1	0	1
cysK	1	1	1	0	0	0
cysM	4	3	3	-1	-1	0
cysN	3	3	2	0	-1	1
cysP	4	4	3	0	-1	1
cysQ	2	2	1	0	-1	1
cysS	1	2	2	1	1	0
cysT	4	4	4	0	0	0
cysU	4	4	3	0	-1	1
cysW	4	3	3	-1	-1	0
cysZ	3	4	3	1	0	1
cytR	4	4	4	0	0	0
cyuA	2	3	2	1	0	1
cyuP	3	4	3	1	0	1
dacA	1	1	1	0	0	0
dacB	4	4	3	0	-1	1
dacC	3	4	3	1	0	1
dacD	3	4	3	1	0	1
dadA	2	3	1	1	-1	2
dadX	2	3	1	1	-1	2
dam	2	3	3	1	1	0
damX	4	4	3	0	-1	1
dapA	2	3	2	1	0	1
dapB	1	1	1	0	0	0
dapD	1	1	1	0	0	0
dapE	3	3	2	0	-1	1
dapF	4	4	3	0	-1	1
dauA	1	1	1	0	0	0

dbpA	4	4	4	0	0	0
dcd	2	2	2	0	0	0
dcm	4	4	4	0	0	0
dcp	1	1	1	0	0	0
dcrB	1	1	1	0	0	0
dctA	3	3	3	0	0	0
dctR	4	4	3	0	-1	1
dcuA	1	1	1	0	0	0
dcuB	4	3	3	-1	-1	0
dcuC	4	3	3	-1	-1	0
dcuD	4	4	4	0	0	0
dcuR	4	4	4	0	0	0
dcuS	4	4	4	0	0	0
dcyD	2	3	2	1	0	1
ddlA	1	1	1	0	0	0
ddlB	1	1	1	0	0	0
ddpA	1	3	2	2	1	1
ddpB	2	3	3	1	1	0
ddpC	3	4	3	1	0	1
ddpD	2	3	3	1	1	0
ddpF	1	2	2	1	1	0
ddpX	3	4	3	1	0	1
deaD	1	2	1	1	0	1
decR	4	4	4	0	0	0
dedA	1	2	2	1	1	0
dedD	2	3	2	1	0	1
def	4	4	3	0	-1	1
degP	1	1	1	0	0	0
degQ	2	3	2	1	0	1
degS	4	4	3	0	-1	1
deoA	1	2	1	1	0	1
deoB	1	1	1	0	0	0
deoC	2	3	2	1	0	1
deoD	1	1	1	0	0	0
deoR	4	4	3	0	-1	1
der	1	1	1	0	0	0
dfp	2	4	3	2	1	1
dgcC	4	4	4	0	0	0
dgcE	4	4	4	0	0	0
dgcF	4	4	4	0	0	0
dgcI	3	4	3	1	0	1
dgcJ	4	4	4	0	0	0
dgcM	4	3	3	-1	-1	0
dgcN	4	4	3	0	-1	1
dgcP	4	4	4	0	0	0
dgcQ	4	4	3	0	-1	1
dgcT	4	4	4	0	0	0
dgcZ	4	3	3	-1	-1	0
dgkA	4	4	4	0	0	0
dgoA	3	3	2	0	-1	1
dgoD	2	3	2	1	0	1

dgoK	3	2	2	-1	-1	0
dgoR	3	3	2	0	-1	1
dgoT	1	3	3	2	2	0
dgt	3	4	3	1	0	1
dhaK	2	2	1	0	-1	1
dhaL	2	2	1	0	-1	1
dhaM	2	1	1	-1	-1	0
dhaR	4	4	4	0	0	0
diaA	3	3	2	0	-1	1
dicA	4	4	4	0	0	0
dicB	4	4	4	0	0	0
dinB	4	4	4	0	0	0
dinD	4	4	3	0	-1	1
dinF	4	4	3	0	-1	1
dinG	4	4	4	0	0	0
dinI	2	3	2	1	0	1
dinJ	4	4	3	0	-1	1
dinQ	1	1	1	0	0	0
djlA	4	4	4	0	0	0
djlB	4	4	4	0	0	0
djlC	4	4	3	0	-1	1
dkgA	1	1	1	0	0	0
dkgB	4	4	3	0	-1	1
dksA	1	1	1	0	0	0
dld	1	1	1	0	0	0
dmlA	1	2	2	1	1	0
dmlR	4	4	4	0	0	0
dmsA	1	1	1	0	0	0
dmsB	1	1	1	0	0	0
dmsC	1	1	1	0	0	0
dmsD	3	3	3	0	0	0
dnaA	4	4	4	0	0	0
dnaB	4	4	3	0	-1	1
dnaC	4	4	3	0	-1	1
dnaE	1	1	1	0	0	0
dnaG	4	4	4	0	0	0
dnaJ	1	2	2	1	1	0
dnaK	2	3	2	1	0	1
dnaN	4	4	3	0	-1	1
dnaQ	4	4	4	0	0	0
dnaT	4	4	4	0	0	0
dnaX	4	4	3	0	-1	1
dosC	4	4	3	0	-1	1
dosP	4	4	3	0	-1	1
dpiA	3	4	3	1	0	1
dpiB	4	4	3	0	-1	1
dppA	1	1	1	0	0	0
dppB	3	3	2	0	-1	1
dppC	2	2	1	0	-1	1
dppD	1	2	1	1	0	1
dppF	1	1	1	0	0	0

dps	1	1	1	0	0	0
dsbA	1	2	1	1	0	1
dsbB	3	4	3	1	0	1
dsbC	3	4	3	1	0	1
dsbD	4	4	3	0	-1	1
dsbG	1	1	1	0	0	0
dsdA	1	2	1	1	0	1
dsdC	4	4	4	0	0	0
dsdX	2	3	2	1	0	1
dsrA	4	3	2	-1	-2	1
dsrB	3	3	3	0	0	0
dtd	3	3	2	0	-1	1
dtpA	1	2	2	1	1	0
dtpB	3	3	3	0	0	0
dtpC	4	3	2	-1	-2	1
dtpD	4	3	3	-1	-1	0
dusA	3	4	3	1	0	1
dusB	3	3	3	0	0	0
dusC	4	4	3	0	-1	1
dut	2	3	3	1	1	0
dxr	3	3	3	0	0	0
dxs	4	4	3	0	-1	1
eamA	4	4	4	0	0	0
eamB	2	1	1	-1	-1	0
ebgA	4	4	3	0	-1	1
ebgC	3	3	2	0	-1	1
ebgR	2	3	2	1	0	1
ecnA	4	4	4	0	0	0
ecnB	2	3	3	1	1	0
eco	1	1	1	0	0	0
ecpA	1	2	2	1	1	0
ecpB	4	3	2	-1	-2	1
ecpC	4	4	3	0	-1	1
ecpD	4	2	2	-2	-2	0
ecpE	4	4	3	0	-1	1
ecpR	3	3	2	0	-1	1
eda	1	1	1	0	0	0
edd	1	1	1	0	0	0
efeB	4	3	3	-1	-1	0
efeO	2	3	2	1	0	1
efp	3	3	2	0	-1	1
elaA	1	1	1	0	0	0
elaB	1	1	1	0	0	0
elaD	1	1	1	0	0	0
elbB	1	1	1	0	0	0
elfA	1	1	1	0	0	0
elfC	4	4	4	0	0	0
elfD	4	4	3	0	-1	1
elfG	4	3	2	-1	-2	1
elyC	3	3	2	0	-1	1
emrA	1	1	1	0	0	0

emrB	1	1	1	0	0	0
emrD	4	4	3	0	-1	1
emrK	4	4	3	0	-1	1
emrY	4	4	3	0	-1	1
emtA	3	3	3	0	0	0
endA	3	3	3	0	0	0
eno	1	1	1	0	0	0
entA	2	2	1	0	-1	1
entB	1	2	1	1	0	1
entC	3	3	2	0	-1	1
entD	4	4	3	0	-1	1
entE	2	2	1	0	-1	1
entF	2	3	2	1	0	1
entH	2	3	1	1	-1	2
entS	4	4	3	0	-1	1
envC	4	4	4	0	0	0
envR	4	4	4	0	0	0
envY	4	4	4	0	0	0
envZ	4	4	3	0	-1	1
epd	4	4	4	0	0	0
epmA	3	3	3	0	0	0
epmB	4	4	4	0	0	0
epmC	4	3	2	-1	-2	1
eptA	4	4	4	0	0	0
eptB	4	4	4	0	0	0
eptC	2	2	2	0	0	0
era	4	4	4	0	0	0
erpA	3	3	2	0	-1	1
esrE	4	4	3	0	-1	1
etk	1	1	1	0	0	0
etp	2	3	2	1	0	1
ettA	1	1	1	0	0	0
eutA	1	1	1	0	0	0
eutB	1	1	1	0	0	0
eutC	1	1	1	0	0	0
eutD	1	1	1	0	0	0
eutE	1	1	1	0	0	0
eutG	1	1	1	0	0	0
eutH	1	1	1	0	0	0
eutJ	1	1	1	0	0	0
eutK	1	1	1	0	0	0
eutL	1	1	1	0	0	0
eutM	1	1	1	0	0	0
eutN	1	1	1	0	0	0
eutP	1	2	1	1	0	1
eutQ	1	1	1	0	0	0
eutR	3	3	4	0	1	-1
eutS	3	3	3	0	0	0
eutT	1	1	1	0	0	0
evgA	4	4	4	0	0	0
evgS	4	4	4	0	0	0

exbB	4	4	4	0	0	0
exbD	4	4	4	0	0	0
exoX	4	4	4	0	0	0
exuR	4	1	1	-3	-3	0
exuT	1	2	2	1	1	0
eyeA	3	3	2	0	-1	1
fabA	1	1	1	0	0	0
fabB	1	2	2	1	1	0
fabD	1	1	1	0	0	0
fabF	1	1	1	0	0	0
fabG	1	1	1	0	0	0
fabH	2	3	2	1	0	1
fabI	1	1	1	0	0	0
fabR	2	1	1	-1	-1	0
fabZ	1	1	1	0	0	0
fadA	1	3	1	2	0	2
fadB	1	3	1	2	0	2
fadD	3	4	2	1	-1	2
fadE	2	3	1	1	-1	2
fadH	4	4	3	0	-1	1
fadI	4	4	3	0	-1	1
fadJ	3	4	3	1	0	1
fadK	2	3	3	1	1	0
fadL	1	2	1	1	0	1
fadM	2	3	3	1	1	0
fadR	4	4	4	0	0	0
fau	4	4	4	0	0	0
fbaA	1	1	1	0	0	0
fbaB	1	1	1	0	0	0
fbp	1	1	1	0	0	0
fcl	2	1	1	-1	-1	0
fdhD	4	4	4	0	0	0
fdhE	1	1	1	0	0	0
fdhF	2	3	2	1	0	1
fdnG	2	3	2	1	0	1
fdnH	1	2	2	1	1	0
fdnI	2	2	2	0	0	0
fdoG	3	3	3	0	0	0
fdoH	2	2	1	0	-1	1
fdoI	1	2	1	1	0	1
fdrA	2	2	1	0	-1	1
fdx	1	1	1	0	0	0
feaR	4	4	4	0	0	0
feoA	4	4	4	0	0	0
feoB	4	3	3	-1	-1	0
feoC	3	2	2	-1	-1	0
fepA	3	3	2	0	-1	1
fepB	4	4	3	0	-1	1
fepC	4	4	4	0	0	0
fepD	4	3	3	-1	-1	0
fepE	4	4	4	0	0	0

fepG	4	1	2	-3	-2	-1
fes	4	3	3	-1	-1	0
fetA	4	4	4	0	0	0
fetB	4	4	3	0	-1	1
ffh	4	4	3	0	-1	1
ffs	1	2	2	1	1	0
fhlA	4	3	3	-1	-1	0
fhuA	4	4	4	0	0	0
fhuB	4	2	3	-2	-1	-1
fhuC	4	4	4	0	0	0
fhuD	4	4	4	0	0	0
fhuE	3	3	2	0	-1	1
fhuF	4	4	4	0	0	0
fic	3	3	2	0	-1	1
fieF	3	3	3	0	0	0
fimG	3	3	2	0	-1	1
fimH	4	4	3	0	-1	1
fimZ	4	3	3	-1	-1	0
fis	2	2	2	0	0	0
fiu	1	3	1	2	0	2
fixA	1	1	1	0	0	0
fixB	1	1	1	0	0	0
fixC	1	1	1	0	0	0
fixX	2	1	1	-1	-1	0
fklB	1	1	1	0	0	0
fkpA	1	1	1	0	0	0
fkpB	1	1	1	0	0	0
fldA	4	4	3	0	-1	1
fldB	4	4	3	0	-1	1
flgA	4	4	3	0	-1	1
flgC	4	4	3	0	-1	1
flgD	3	2	2	-1	-1	0
flgE	2	3	2	1	0	1
flgF	2	3	3	1	1	0
flgG	1	1	1	0	0	0
flgH	3	3	2	0	-1	1
flgI	3	3	2	0	-1	1
flgJ	2	3	3	1	1	0
flgK	1	3	2	2	1	1
flgL	1	3	2	2	1	1
flgM	4	4	4	0	0	0
flgN	4	4	4	0	0	0
flhA	4	3	3	-1	-1	0
flhB	4	2	2	-2	-2	0
flhC	4	4	3	0	-1	1
flhD	4	4	3	0	-1	1
flhE	4	4	4	0	0	0
fliA	4	4	4	0	0	0
fliC	3	4	3	1	0	1
fliD	4	4	3	0	-1	1
fliE	3	2	2	-1	-1	0

fliF	4	3	2	-1	-2	1
fliG	4	3	2	-1	-2	1
fliH	4	3	3	-1	-1	0
fliI	3	3	2	0	-1	1
fliJ	4	3	3	-1	-1	0
fliK	4	3	3	-1	-1	0
fliL	4	3	3	-1	-1	0
fliM	3	3	3	0	0	0
fliN	2	2	2	0	0	0
fliO	3	2	2	-1	-1	0
fliP	3	3	3	0	0	0
fliQ	3	3	3	0	0	0
fliR	4	4	3	0	-1	1
fliS	4	3	3	-1	-1	0
fliT	4	3	2	-1	-2	1
fliZ	4	3	2	-1	-2	1
flk	4	3	3	-1	-1	0
flxA	2	3	2	1	0	1
fmt	4	3	3	-1	-1	0
fnr	4	4	4	0	0	0
fnrS	3	3	3	0	0	0
focA	3	3	3	0	0	0
focB	4	4	4	0	0	0
folA	4	4	4	0	0	0
folB	4	4	4	0	0	0
folC	2	3	2	1	0	1
folD	3	4	3	1	0	1
folE	2	3	3	1	1	0
folK	4	4	4	0	0	0
folM	4	4	4	0	0	0
folP	1	2	2	1	1	0
folX	1	1	1	0	0	0
fpr	2	3	2	1	0	1
frc	3	3	2	0	-1	1
frdA	1	1	1	0	0	0
frdB	1	1	1	0	0	0
frdC	1	1	1	0	0	0
frdD	1	1	1	0	0	0
fre	4	4	3	0	-1	1
frlA	1	2	1	1	0	1
frlB	1	1	1	0	0	0
frlC	1	1	1	0	0	0
frlD	2	1	1	-1	-1	0
frlR	3	2	2	-1	-1	0
frmA	1	1	1	0	0	0
frmB	1	1	1	0	0	0
frmR	1	1	1	0	0	0
frr	1	1	1	0	0	0
frsA	4	4	4	0	0	0
fruA	2	2	1	0	-1	1
fruB	3	3	1	0	-2	2

fruK	3	3	1	0	-2	2
frvA	4	4	4	0	0	0
frvB	4	3	3	-1	-1	0
frvR	4	4	3	0	-1	1
frvX	4	4	3	0	-1	1
frwB	2	1	1	-1	-1	0
frwC	3	2	3	-1	0	-1
frwD	4	4	4	0	0	0
fryA	3	3	2	0	-1	1
fryC	3	3	2	0	-1	1
fsaA	4	4	4	0	0	0
fsaB	1	1	1	0	0	0
fsr	3	3	3	0	0	0
ftnA	1	1	1	0	0	0
ftnB	3	2	2	-1	-1	0
ftp	4	4	3	0	-1	1
ftsA	1	1	1	0	0	0
ftsB	4	4	4	0	0	0
ftsE	4	2	2	-2	-2	0
ftsH	3	3	2	0	-1	1
ftsI	4	4	3	0	-1	1
ftsK	4	4	3	0	-1	1
ftsL	4	4	3	0	-1	1
ftsN	4	4	4	0	0	0
ftsP	3	3	3	0	0	0
ftsQ	1	1	1	0	0	0
ftsW	1	1	1	0	0	0
ftsX	4	4	3	0	-1	1
ftsY	4	4	4	0	0	0
ftsZ	1	1	1	0	0	0
fucA	1	1	1	0	0	0
fucI	1	1	1	0	0	0
fucK	1	1	1	0	0	0
fucO	1	1	1	0	0	0
fucP	4	4	4	0	0	0
fucR	3	3	2	0	-1	1
fucU	1	1	1	0	0	0
fumA	1	1	1	0	0	0
fumB	2	2	2	0	0	0
fumC	1	1	1	0	0	0
fumD	4	3	2	-1	-2	1
fumE	4	3	3	-1	-1	0
fur	4	4	3	0	-1	1
fusA	1	1	1	0	0	0
fxsA	4	4	3	0	-1	1
gabD	1	1	1	0	0	0
gabP	1	1	1	0	0	0
gabT	1	1	1	0	0	0
gadA	1	2	2	1	1	0
gadB	1	1	1	0	0	0
gadC	1	2	1	1	0	1

gadE	4	3	2	-1	-2	1
gadF	2	2	3	0	1	-1
gadW	4	4	3	0	-1	1
gadX	4	4	4	0	0	0
gadY	4	4	4	0	0	0
galE	3	3	2	0	-1	1
galF	3	4	3	1	0	1
galK	2	1	1	-1	-1	0
galM	1	1	1	0	0	0
galP	4	3	3	-1	-1	0
galR	4	4	3	0	-1	1
galS	4	4	4	0	0	0
galT	3	2	1	-1	-2	1
galU	1	1	1	0	0	0
gapA	1	1	1	0	0	0
garD	2	3	3	1	1	0
garK	1	1	1	0	0	0
garL	3	3	3	0	0	0
garP	4	4	4	0	0	0
garR	1	1	1	0	0	0
gcd	1	1	1	0	0	0
gcl	3	3	2	0	-1	1
gcvA	4	4	4	0	0	0
gcvB	3	3	2	0	-1	1
gcvH	1	2	1	1	0	1
gcvP	1	1	1	0	0	0
gcvR	3	3	2	0	-1	1
gcvT	1	2	1	1	0	1
gdhA	3	2	2	-1	-1	0
gdx	4	4	4	0	0	0
gfcA	1	1	1	0	0	0
gfcB	4	4	4	0	0	0
gfcC	4	4	4	0	0	0
gfcD	4	4	4	0	0	0
gfcE	3	4	4	1	1	0
ggt	1	1	1	0	0	0
ghoS	4	4	4	0	0	0
ghoT	4	4	4	0	0	0
ghrA	2	2	1	0	-1	1
ghrB	1	1	1	0	0	0
ghxP	2	3	3	1	1	0
ghxQ	2	2	2	0	0	0
glcA	2	3	2	1	0	1
glcB	1	1	1	0	0	0
glcC	4	4	3	0	-1	1
glcD	3	4	3	1	0	1
glcE	2	3	2	1	0	1
glcF	3	3	1	0	-2	2
glcG	1	1	1	0	0	0
gldA	1	1	1	0	0	0
glgA	1	1	1	0	0	0

glgB	1	1	1	0	0	0
glgC	1	1	1	0	0	0
glgP	1	1	1	0	0	0
glgS	3	3	2	0	-1	1
glgX	1	1	1	0	0	0
glk	4	3	3	-1	-1	0
glmM	2	2	1	0	-1	1
glmS	3	3	1	0	-2	2
glmU	4	4	3	0	-1	1
glmY	2	1	1	-1	-1	0
glmZ	4	2	1	-2	-3	1
glnA	1	2	2	1	1	0
glnB	1	1	1	0	0	0
glnD	4	4	3	0	-1	1
glnE	3	4	3	1	0	1
glnG	1	2	2	1	1	0
glnH	1	1	1	0	0	0
glnK	1	2	1	1	0	1
glnL	2	3	3	1	1	0
glnP	1	1	1	0	0	0
glnQ	1	1	1	0	0	0
glnS	3	3	3	0	0	0
glnU	4	4	4	0	0	0
glnV	4	3	3	-1	-1	0
glnW	4	4	4	0	0	0
glnX	4	3	2	-1	-2	1
gloA	2	2	2	0	0	0
gloB	2	1	1	-1	-1	0
gloC	2	3	2	1	0	1
glpA	4	4	3	0	-1	1
glpB	3	3	2	0	-1	1
glpC	2	2	1	0	-1	1
glpD	4	4	4	0	0	0
glpE	4	4	4	0	0	0
glpF	1	2	2	1	1	0
glpG	4	4	4	0	0	0
glpK	1	1	1	0	0	0
glpQ	1	1	1	0	0	0
glpT	2	1	1	-1	-1	0
glpX	2	2	2	0	0	0
glrK	4	4	3	0	-1	1
glrR	4	4	3	0	-1	1
glsA	4	3	2	-1	-2	1
glsB	4	4	4	0	0	0
gltA	1	1	1	0	0	0
gltB	2	3	2	1	0	1
gltD	1	2	1	1	0	1
gltF	1	1	1	0	0	0
gltI	1	2	1	1	0	1
gltJ	4	4	3	0	-1	1
gltK	3	4	3	1	0	1

gltL	2	3	2	1	0	1
gltP	4	4	3	0	-1	1
gltS	3	4	3	1	0	1
gltT	4	4	4	0	0	0
gltU	4	4	4	0	0	0
gltV	4	4	4	0	0	0
gltW	4	4	4	0	0	0
gltX	1	2	1	1	0	1
gluQ	4	4	4	0	0	0
glvB	4	4	4	0	0	0
glvC	3	4	3	1	0	1
glvG	3	4	3	1	0	1
glxK	1	2	1	1	0	1
glxR	1	1	1	0	0	0
glyA	1	1	1	0	0	0
glyQ	1	2	2	1	1	0
glyS	1	1	1	0	0	0
glyT	4	4	4	0	0	0
glyU	4	4	4	0	0	0
glyV	4	4	4	0	0	0
glyW	4	4	4	0	0	0
glyX	3	4	4	1	1	0
glyY	4	4	4	0	0	0
gmd	2	1	1	-1	-1	0
gmhB	4	3	3	-1	-1	0
gmk	4	4	3	0	-1	1
gmm	2	1	1	-1	-1	0
gnd	1	1	1	0	0	0
gnsA	2	2	2	0	0	0
gnsB	1	1	1	0	0	0
gntK	3	3	1	0	-2	2
gntP	4	3	2	-1	-2	1
gntR	4	4	4	0	0	0
gntT	3	3	2	0	-1	1
gntU	4	3	3	-1	-1	0
gor	1	2	2	1	1	0
gph	1	1	1	0	0	0
gpmA	2	2	1	0	-1	1
gpmM	2	3	2	1	0	1
gpp	4	4	4	0	0	0
gpr	1	2	1	1	0	1
gpsA	2	2	1	0	-1	1
gpt	4	4	4	0	0	0
grcA	1	3	1	2	0	2
greA	3	4	3	1	0	1
greB	3	2	2	-1	-1	0
groL	1	1	1	0	0	0
groS	1	1	1	0	0	0
grpE	1	2	1	1	0	1
grxA	3	4	3	1	0	1
grxB	1	1	1	0	0	0

grxC	1	1	1	0	0	0
grxD	1	1	1	0	0	0
gshA	3	3	3	0	0	0
gshB	3	3	2	0	-1	1
gsiA	3	4	3	1	0	1
gsiB	1	3	2	2	1	1
gsiC	1	2	2	1	1	0
gsiD	1	2	2	1	1	0
gsk	4	4	4	0	0	0
gspA	4	4	3	0	-1	1
gspB	4	4	3	0	-1	1
gspC	4	4	4	0	0	0
gspD	4	4	3	0	-1	1
gspE	4	4	3	0	-1	1
gspF	4	3	3	-1	-1	0
gspG	4	3	3	-1	-1	0
gspH	3	2	2	-1	-1	0
gspI	4	3	3	-1	-1	0
gspJ	4	3	3	-1	-1	0
gspK	4	3	3	-1	-1	0
gspL	4	4	3	0	-1	1
gspM	4	3	3	-1	-1	0
gspO	4	4	4	0	0	0
gss	1	2	1	1	0	1
gstA	1	1	1	0	0	0
gstB	1	1	1	0	0	0
guaA	1	1	1	0	0	0
guaB	3	4	3	1	0	1
guaC	1	1	1	0	0	0
guaD	2	3	3	1	1	0
gudD	1	1	1	0	0	0
gudP	3	3	3	0	0	0
gudX	3	3	2	0	-1	1
gutM	2	3	3	1	1	0
gutQ	1	1	1	0	0	0
gyrA	1	1	1	0	0	0
gyrB	1	1	1	0	0	0
hcaB	1	3	2	2	1	1
hcaC	2	3	2	1	0	1
hcaD	1	2	1	1	0	1
hcaE	3	3	3	0	0	0
hcaF	3	3	3	0	0	0
hcaR	4	4	3	0	-1	1
hcaT	4	4	3	0	-1	1
hchA	4	4	3	0	-1	1
hcp	4	3	2	-1	-2	1
hcr	3	3	2	0	-1	1
hcxA	4	4	3	0	-1	1
hcxB	1	1	1	0	0	0
hda	4	4	4	0	0	0
hdeA	1	1	1	0	0	0

hdeB	2	1	1	-1	-1	0
hdeD	4	4	3	0	-1	1
hdfR	3	3	3	0	0	0
hdhA	1	1	1	0	0	0
helD	4	4	4	0	0	0
hemA	4	4	4	0	0	0
hemB	2	3	2	1	0	1
hemC	4	4	3	0	-1	1
hemD	4	4	4	0	0	0
hemE	2	2	1	0	-1	1
hemF	4	4	4	0	0	0
hemG	3	3	2	0	-1	1
hemH	4	4	3	0	-1	1
hemL	1	1	1	0	0	0
hemN	3	3	3	0	0	0
hemX	3	4	3	1	0	1
hemY	2	3	2	1	0	1
hflC	1	1	1	0	0	0
hflD	4	4	4	0	0	0
hflK	1	1	1	0	0	0
hflX	3	3	2	0	-1	1
hfq	1	1	1	0	0	0
hha	4	4	3	0	-1	1
hicA	4	4	4	0	0	0
hicB	4	4	4	0	0	0
higA	3	3	3	0	0	0
higB	4	4	3	0	-1	1
hinT	1	1	1	0	0	0
hipA	4	4	4	0	0	0
hipB	4	4	4	0	0	0
hisA	1	1	1	0	0	0
hisB	1	2	1	1	0	1
hisC	1	2	1	1	0	1
hisD	2	3	2	1	0	1
hisF	1	1	1	0	0	0
hisG	2	3	3	1	1	0
hisH	1	2	1	1	0	1
hisI	1	1	1	0	0	0
hisJ	2	2	2	0	0	0
hisL	4	4	4	0	0	0
hisM	3	4	3	1	0	1
hisP	1	3	2	2	1	1
hisQ	4	4	3	0	-1	1
hisR	4	4	4	0	0	0
hisS	3	3	3	0	0	0
hlyE	3	4	4	1	1	0
hmp	1	1	1	0	0	0
hns	1	1	1	0	0	0
hofB	4	2	2	-2	-2	0
hofC	4	3	3	-1	-1	0
hofM	4	4	3	0	-1	1

hofN	4	3	3	-1	-1	0
hofO	4	4	3	0	-1	1
hofP	4	4	3	0	-1	1
hofQ	4	4	3	0	-1	1
hokA	1	1	1	0	0	0
hokB	1	1	1	0	0	0
hokC	4	4	3	0	-1	1
hokD	1	1	1	0	0	0
hokE	4	4	3	0	-1	1
holA	4	3	3	-1	-1	0
holB	3	3	2	0	-1	1
holC	3	4	3	1	0	1
holD	4	4	4	0	0	0
holE	4	4	4	0	0	0
hpf	1	1	1	0	0	0
hprR	4	4	4	0	0	0
hprS	4	4	4	0	0	0
hpt	2	2	2	0	0	0
hrpA	4	4	4	0	0	0
hrpB	4	4	3	0	-1	1
hscA	2	3	2	1	0	1
hscB	3	3	2	0	-1	1
hscC	4	3	2	-1	-2	1
hslJ	2	2	2	0	0	0
hslO	2	3	2	1	0	1
hslR	3	3	2	0	-1	1
hslU	1	1	1	0	0	0
hslV	1	2	1	1	0	1
hspQ	3	3	2	0	-1	1
hsrA	4	4	3	0	-1	1
htpG	1	1	1	0	0	0
htpX	3	3	2	0	-1	1
htrE	4	3	3	-1	-1	0
hupA	1	1	1	0	0	0
hupB	1	1	1	0	0	0
hxpA	1	1	1	0	0	0
hxpB	1	1	1	0	0	0
hyaA	2	2	2	0	0	0
hyaB	2	1	2	-1	0	-1
hyaC	2	1	2	-1	0	-1
hyaD	2	2	2	0	0	0
hyaE	2	2	2	0	0	0
hyaF	1	1	1	0	0	0
hybA	2	2	1	0	-1	1
hybB	1	1	1	0	0	0
hybC	1	1	1	0	0	0
hybD	1	1	1	0	0	0
hybE	1	1	1	0	0	0
hybF	2	1	1	-1	-1	0
hybG	2	1	1	-1	-1	0
hybO	2	2	2	0	0	0

hycA	3	3	3	0	0	0
hycB	3	2	2	-1	-1	0
hycC	3	2	2	-1	-1	0
hycD	2	2	2	0	0	0
hycE	3	3	2	0	-1	1
hycF	2	3	2	1	0	1
hycG	3	3	2	0	-1	1
hycH	2	3	2	1	0	1
hycI	1	2	2	1	1	0
hydN	4	4	4	0	0	0
hyfA	4	4	3	0	-1	1
hyfB	4	3	3	-1	-1	0
hyfC	4	3	3	-1	-1	0
hyfD	4	4	3	0	-1	1
hyfE	4	4	4	0	0	0
hyfF	4	4	3	0	-1	1
hyfG	4	3	3	-1	-1	0
hyfH	3	3	3	0	0	0
hyfI	3	3	2	0	-1	1
hyfJ	3	3	2	0	-1	1
hyfR	4	3	3	-1	-1	0
hyi	1	3	1	2	0	2
hypA	2	2	2	0	0	0
hypB	2	2	1	0	-1	1
hypC	1	2	1	1	0	1
hypD	2	2	1	0	-1	1
hypE	1	1	1	0	0	0
hypF	3	4	3	1	0	1
hypT	4	4	4	0	0	0
hyuA	4	3	3	-1	-1	0
iaaA	4	4	3	0	-1	1
iadA	1	1	1	0	0	0
iap	4	4	4	0	0	0
ibaG	2	3	2	1	0	1
ibpA	4	4	3	0	-1	1
ibpB	3	4	1	1	-2	3
ibsA	4	2	3	-2	-1	-1
ibsB	4	4	4	0	0	0
ibsC	2	2	2	0	0	0
ibsD	4	1	1	-3	-3	0
ibsE	2	3	2	1	0	1
icd	1	1	1	0	0	0
iclR	4	4	4	0	0	0
idi	4	4	3	0	-1	1
idlP	4	4	3	0	-1	1
igaA	4	4	4	0	0	0
ihfA	1	1	1	0	0	0
ihfB	1	1	1	0	0	0
ileS	1	2	1	1	0	1
ileT	4	4	4	0	0	0
ileU	4	4	4	0	0	0

ileV	4	4	4	0	0	0
ileX	3	4	3	1	0	1
ileY	4	4	4	0	0	0
ilvA	1	2	2	1	1	0
ilvB	2	3	2	1	0	1
ilvC	4	4	3	0	-1	1
ilvD	2	3	2	1	0	1
ilvE	1	1	1	0	0	0
ilvH	3	3	2	0	-1	1
ilvI	3	3	2	0	-1	1
ilvL	4	4	4	0	0	0
ilvM	2	3	2	1	0	1
ilvN	2	2	1	0	-1	1
ilvX	4	4	3	0	-1	1
ilvY	4	4	3	0	-1	1
inaA	4	4	3	0	-1	1
infA	2	2	2	0	0	0
infB	2	2	1	0	-1	1
infC	2	1	1	-1	-1	0
insA-1	4	4	4	0	0	0
insA-2	4	4	4	0	0	0
insA-3	4	4	4	0	0	0
insA-4	4	4	4	0	0	0
insA-5	4	4	4	0	0	0
insA-6	4	4	4	0	0	0
insA9	4	4	4	0	0	0
insB-1	4	4	4	0	0	0
insB-2	4	4	4	0	0	0
insB-3	4	4	4	0	0	0
insB-4	4	4	4	0	0	0
insB-5	4	4	4	0	0	0
insB-6	4	4	4	0	0	0
insB9	4	4	4	0	0	0
insE-1	3	3	3	0	0	0
insE-2	3	3	3	0	0	0
insE-3	3	3	3	0	0	0
insE-4	3	3	3	0	0	0
insE-5	3	3	3	0	0	0
insF-1	3	3	3	0	0	0
insF-2	3	3	3	0	0	0
insF-3	3	3	3	0	0	0
insF-4	3	3	3	0	0	0
insF-5	3	3	3	0	0	0
insI-1	3	4	3	1	0	1
insI-2	3	4	3	1	0	1
insI-3	3	4	3	1	0	1
insL-1	3	3	2	0	-1	1
insL-2	3	3	2	0	-1	1
insL-3	3	3	2	0	-1	1
insQ	3	4	3	1	0	1
iprA	4	4	4	0	0	0

iraD	4	4	3	0	-1	1
iraM	4	3	3	-1	-1	0
iraP	3	3	2	0	-1	1
iroK	4	4	4	0	0	0
iscA	2	2	1	0	-1	1
iscR	2	3	1	1	-1	2
iscS	2	3	1	1	-1	2
iscU	2	3	1	1	-1	2
iscX	1	1	1	0	0	0
ispA	4	4	4	0	0	0
ispB	4	4	4	0	0	0
ispD	4	4	3	0	-1	1
ispE	1	1	1	0	0	0
ispF	2	1	1	-1	-1	0
ispG	4	4	3	0	-1	1
ispH	1	1	1	0	0	0
ispU	4	4	3	0	-1	1
istR	4	4	3	0	-1	1
ivbL	4	4	4	0	0	0
ivy	1	1	1	0	0	0
katE	1	1	1	0	0	0
katG	1	1	1	0	0	0
kbaY	3	3	3	0	0	0
kbaZ	1	3	2	2	1	1
kbl	1	1	1	0	0	0
kbp	1	1	1	0	0	0
kch	3	4	3	1	0	1
kdgK	4	4	3	0	-1	1
kdgR	3	4	3	1	0	1
kdgT	3	4	3	1	0	1
kdpA	3	3	3	0	0	0
kdpB	3	3	2	0	-1	1
kdpC	3	3	2	0	-1	1
kdpD	4	4	3	0	-1	1
kdpE	4	4	3	0	-1	1
kdsA	1	2	2	1	1	0
kdsB	2	1	1	-1	-1	0
kdsC	4	4	3	0	-1	1
kdsD	4	4	4	0	0	0
kduD	1	1	1	0	0	0
kduI	1	2	1	1	0	1
kefB	4	4	3	0	-1	1
kefC	3	3	3	0	0	0
kefF	3	4	3	1	0	1
kefG	4	4	3	0	-1	1
kgtP	2	2	1	0	-1	1
kptA	4	4	3	0	-1	1
kup	4	4	4	0	0	0
lacA	1	3	3	2	2	0
lacI	4	4	4	0	0	0
lacY	1	2	2	1	1	0

lacZ	1	3	2	2	1	1
lamB	1	1	1	0	0	0
lapA	4	4	4	0	0	0
lapB	4	4	4	0	0	0
ldcA	4	4	3	0	-1	1
ldcC	1	1	1	0	0	0
ldhA	1	2	2	1	1	0
ldrA	1	1	1	0	0	0
ldrB	1	1	1	0	0	0
ldrC	1	1	1	0	0	0
ldrD	1	1	1	0	0	0
ldtA	4	4	3	0	-1	1
ldtB	3	4	3	1	0	1
ldtC	3	3	3	0	0	0
ldtD	3	3	2	0	-1	1
ldtE	1	1	1	0	0	0
lepA	3	4	3	1	0	1
lepB	2	3	2	1	0	1
leuA	3	3	2	0	-1	1
leuB	1	2	1	1	0	1
leuC	1	2	1	1	0	1
leuD	2	3	2	1	0	1
leuE	4	4	3	0	-1	1
leuO	4	4	2	0	-2	2
leuP	3	4	4	1	1	0
leuQ	3	4	4	1	1	0
leuS	3	3	3	0	0	0
leuT	4	4	3	0	-1	1
leuU	4	4	3	0	-1	1
leuV	4	4	4	0	0	0
leuW	4	3	3	-1	-1	0
leuX	4	4	4	0	0	0
leuZ	4	4	4	0	0	0
lexA	4	4	3	0	-1	1
lgoD	4	4	4	0	0	0
lgoR	4	3	3	-1	-1	0
lgoT	3	3	3	0	0	0
lgt	3	4	3	1	0	1
lhgO	1	2	2	1	1	0
lhr	3	4	3	1	0	1
ligA	3	3	3	0	0	0
ligB	4	4	4	0	0	0
lipA	3	4	3	1	0	1
lipB	1	2	2	1	1	0
livF	3	3	3	0	0	0
livG	3	3	3	0	0	0
livH	4	3	2	-1	-2	1
livJ	3	4	3	1	0	1
livK	4	4	3	0	-1	1
livM	3	1	1	-2	-2	0
lldD	1	1	1	0	0	0

lldP	3	4	1	1	-2	3
lldR	3	4	1	1	-2	3
lnt	3	3	2	0	-1	1
loiP	1	1	1	0	0	0
lolA	1	2	2	1	1	0
lolB	3	4	3	1	0	1
lolC	4	4	4	0	0	0
lolD	4	4	4	0	0	0
lolE	4	4	4	0	0	0
lon	1	2	1	1	0	1
lpcA	2	2	2	0	0	0
lpd	1	1	1	0	0	0
lplA	2	2	1	0	-1	1
lplT	4	4	3	0	-1	1
lpoA	4	4	4	0	0	0
lpoB	1	1	1	0	0	0
lpp	1	1	1	0	0	0
lptA	3	3	3	0	0	0
lptB	3	4	3	1	0	1
lptC	4	4	3	0	-1	1
lptD	4	4	4	0	0	0
lptE	1	2	2	1	1	0
lptF	4	4	4	0	0	0
lptG	4	4	3	0	-1	1
lpxA	1	1	1	0	0	0
lpxB	1	1	1	0	0	0
lpxC	1	2	2	1	1	0
lpxD	1	1	1	0	0	0
lpxH	3	3	3	0	0	0
lpxK	4	4	3	0	-1	1
lpxL	4	4	4	0	0	0
lpxM	4	4	4	0	0	0
lpxP	4	4	4	0	0	0
lpxT	3	4	3	1	0	1
lrhA	4	4	3	0	-1	1
lrp	1	1	1	0	0	0
lspA	1	1	1	0	0	0
lsrA	4	4	3	0	-1	1
lsrB	1	2	1	1	0	1
lsrC	4	3	3	-1	-1	0
lsrD	3	4	3	1	0	1
lsrF	1	2	1	1	0	1
lsrG	1	2	2	1	1	0
lsrK	4	4	3	0	-1	1
lsrR	4	4	4	0	0	0
ltaE	1	1	1	0	0	0
luxS	1	1	1	0	0	0
lysA	4	4	3	0	-1	1
lysC	4	4	3	0	-1	1
lysO	3	4	3	1	0	1
lysP	4	2	2	-2	-2	0

lysQ	4	4	4	0	0	0
lysR	4	4	4	0	0	0
lysS	1	1	1	0	0	0
lysT	4	4	4	0	0	0
lysU	1	1	1	0	0	0
lysV	4	4	3	0	-1	1
lysW	2	3	2	1	0	1
lysY	4	4	4	0	0	0
lysZ	4	4	4	0	0	0
lyxK	1	2	2	1	1	0
maa	4	4	3	0	-1	1
macA	4	3	3	-1	-1	0
macB	4	4	3	0	-1	1
maeA	1	1	1	0	0	0
maeB	1	1	1	0	0	0
mak	3	3	2	0	-1	1
malE	1	1	1	0	0	0
malF	4	4	3	0	-1	1
malG	3	3	2	0	-1	1
malI	4	4	3	0	-1	1
malK	3	3	3	0	0	0
malM	3	3	2	0	-1	1
malP	2	3	2	1	0	1
malQ	1	1	1	0	0	0
malS	4	4	4	0	0	0
malT	4	4	4	0	0	0
malX	4	4	3	0	-1	1
malY	3	3	3	0	0	0
malZ	2	2	2	0	0	0
manA	3	3	3	0	0	0
manX	1	1	1	0	0	0
manY	1	1	1	0	0	0
manZ	1	1	1	0	0	0
maoP	3	3	2	0	-1	1
map	4	4	3	0	-1	1
marA	3	3	2	0	-1	1
marB	2	3	2	1	0	1
marC	4	4	3	0	-1	1
marR	4	4	4	0	0	0
matP	3	4	3	1	0	1
mazE	3	4	3	1	0	1
mazF	3	3	2	0	-1	1
mazG	1	2	1	1	0	1
mbiA	4	3	3	-1	-1	0
mcaS	4	4	4	0	0	0
mcbA	1	1	1	0	0	0
mcbR	3	3	3	0	0	0
mdaB	3	3	3	0	0	0
mdfA	4	4	4	0	0	0
mdh	1	1	1	0	0	0
mdlA	4	4	4	0	0	0

mdlB	4	4	3	0	-1	1
mdtA	4	4	3	0	-1	1
mdtB	4	4	3	0	-1	1
mdtC	2	2	2	0	0	0
mdtD	1	1	1	0	0	0
mdtE	2	3	3	1	1	0
mdtF	2	3	3	1	1	0
mdtG	4	4	4	0	0	0
mdtH	4	4	3	0	-1	1
mdtI	4	4	3	0	-1	1
mdtJ	4	4	3	0	-1	1
mdtK	4	4	3	0	-1	1
mdtL	4	4	4	0	0	0
mdtM	4	4	3	0	-1	1
mdtN	4	2	2	-2	-2	0
mdtO	4	4	3	0	-1	1
mdtP	4	3	2	-1	-2	1
melA	1	3	3	2	2	0
melB	3	4	3	1	0	1
melR	4	4	4	0	0	0
menA	4	4	4	0	0	0
menB	1	1	1	0	0	0
menC	1	1	1	0	0	0
menD	3	4	3	1	0	1
menE	1	1	1	0	0	0
menF	4	4	4	0	0	0
menH	1	2	2	1	1	0
menI	1	1	1	0	0	0
mepA	4	4	4	0	0	0
mepH	4	4	4	0	0	0
mepM	4	4	4	0	0	0
mepS	4	4	4	0	0	0
metA	4	4	3	0	-1	1
metB	4	4	3	0	-1	1
metC	4	4	3	0	-1	1
metE	2	2	2	0	0	0
metF	4	4	3	0	-1	1
metG	1	3	2	2	1	1
metH	2	3	3	1	1	0
metI	4	4	3	0	-1	1
metJ	4	3	3	-1	-1	0
metK	3	3	3	0	0	0
metL	4	4	3	0	-1	1
metN	4	4	3	0	-1	1
metQ	1	1	1	0	0	0
metR	4	3	2	-1	-2	1
metT	4	4	3	0	-1	1
metU	4	4	3	0	-1	1
metV	3	1	1	-2	-2	0
metW	2	1	1	-1	-1	0
metY	4	4	4	0	0	0

metZ	2	1	1	-1	-1	0
mfd	4	4	3	0	-1	1
mglA	2	3	2	1	0	1
mglB	1	1	1	0	0	0
mglC	1	3	1	2	0	2
mgrB	4	4	4	0	0	0
mgrR	4	4	3	0	-1	1
mgsA	3	4	3	1	0	1
mgtA	4	3	2	-1	-2	1
mgtL	4	4	4	0	0	0
mgtS	4	4	3	0	-1	1
mhpA	3	3	3	0	0	0
mhpB	2	3	3	1	1	0
mhpC	1	3	2	2	1	1
mhpD	1	2	1	1	0	1
mhpE	1	2	2	1	1	0
mhpF	1	3	1	2	0	2
mhpR	4	4	3	0	-1	1
mhpT	3	2	1	-1	-2	1
miaA	2	1	1	-1	-1	0
miaB	1	3	2	2	1	1
micA	2	3	2	1	0	1
micF	4	4	3	0	-1	1
micL	3	3	3	0	0	0
minC	4	4	4	0	0	0
minD	3	3	2	0	-1	1
minE	2	3	2	1	0	1
mioC	4	4	4	0	0	0
mipA	1	1	1	0	0	0
mlaA	2	2	1	0	-1	1
mlaB	1	2	2	1	1	0
mlaC	1	3	2	2	1	1
mlaD	3	4	3	1	0	1
mlaE	4	4	3	0	-1	1
mlaF	4	4	3	0	-1	1
mlc	4	4	4	0	0	0
mliC	4	3	2	-1	-2	1
mlrA	4	4	4	0	0	0
mltA	4	4	3	0	-1	1
mltB	1	3	3	2	2	0
mltC	4	4	4	0	0	0
mltD	4	3	3	-1	-1	0
mltF	4	4	4	0	0	0
mltG	3	3	3	0	0	0
mmuM	3	3	2	0	-1	1
mmuP	4	3	2	-1	-2	1
mnaT	4	4	4	0	0	0
mngA	1	1	1	0	0	0
mngB	2	1	1	-1	-1	0
mngR	1	1	1	0	0	0
mnmA	4	3	3	-1	-1	0

mnmC	4	4	4	0	0	0
mnmE	1	2	2	1	1	0
mnmG	4	2	2	-2	-2	0
mntH	4	4	3	0	-1	1
mntP	4	4	4	0	0	0
mntR	4	4	4	0	0	0
mntS	4	4	4	0	0	0
moaA	4	4	4	0	0	0
moaB	1	2	1	1	0	1
moaC	1	2	1	1	0	1
moaD	1	2	1	1	0	1
moaE	1	2	1	1	0	1
mobA	4	4	3	0	-1	1
mobB	4	4	3	0	-1	1
mocA	4	4	3	0	-1	1
modA	2	2	2	0	0	0
modB	4	3	3	-1	-1	0
modC	4	4	3	0	-1	1
modE	4	4	3	0	-1	1
modF	4	4	3	0	-1	1
moeA	4	4	3	0	-1	1
moeB	4	4	3	0	-1	1
mog	4	4	3	0	-1	1
mokB	1	1	1	0	0	0
mokC	4	4	3	0	-1	1
motA	4	4	3	0	-1	1
motB	3	3	3	0	0	0
mpaA	4	3	3	-1	-1	0
mpl	3	3	3	0	0	0
mppA	4	4	4	0	0	0
mprA	1	1	1	0	0	0
mqo	1	2	1	1	0	1
mqsA	4	4	1	0	-3	3
mqsR	4	3	1	-1	-3	2
mraY	1	1	1	0	0	0
mraZ	4	4	4	0	0	0
mrcA	4	4	4	0	0	0
mrcB	3	3	3	0	0	0
mrdA	1	1	1	0	0	0
mrdB	1	1	1	0	0	0
mreB	3	4	3	1	0	1
mreC	4	4	3	0	-1	1
mreD	4	4	3	0	-1	1
mrp	1	1	1	0	0	0
msbA	4	4	3	0	-1	1
mscK	4	4	4	0	0	0
mscL	1	1	1	0	0	0
mscM	3	3	3	0	0	0
mscS	1	1	1	0	0	0
msrA	2	3	2	1	0	1
msrB	2	2	2	0	0	0

msrC	4	3	3	-1	-1	0
msrP	3	3	2	0	-1	1
msrQ	4	4	3	0	-1	1
msyB	1	1	1	0	0	0
mtfA	4	4	4	0	0	0
mtgA	3	2	2	-1	-1	0
mtlA	2	3	2	1	0	1
mtlD	4	3	3	-1	-1	0
mtlR	4	4	3	0	-1	1
mtn	1	1	1	0	0	0
mtr	4	3	3	-1	-1	0
mug	2	2	1	0	-1	1
mukB	1	2	1	1	0	1
mukE	4	4	3	0	-1	1
mukF	4	4	4	0	0	0
murA	1	2	2	1	1	0
murB	4	4	4	0	0	0
murC	1	1	1	0	0	0
murD	1	1	1	0	0	0
murE	1	3	2	2	1	1
murF	1	2	1	1	0	1
murG	1	1	1	0	0	0
murI	4	3	3	-1	-1	0
murJ	4	4	4	0	0	0
murP	4	4	3	0	-1	1
murQ	3	3	3	0	0	0
murR	4	4	3	0	-1	1
mutH	4	4	4	0	0	0
mutL	3	3	3	0	0	0
mutM	2	3	3	1	1	0
mutS	4	4	4	0	0	0
mutT	3	4	3	1	0	1
mutY	3	3	3	0	0	0
mzrA	4	4	3	0	-1	1
nac	3	3	3	0	0	0
nadA	4	3	3	-1	-1	0
nadB	4	4	3	0	-1	1
nadC	4	4	3	0	-1	1
nadD	3	3	3	0	0	0
nadE	1	1	1	0	0	0
nadK	4	4	4	0	0	0
nadR	3	4	3	1	0	1
nagA	3	4	3	1	0	1
nagB	3	4	3	1	0	1
nagC	4	4	4	0	0	0
nagE	1	1	1	0	0	0
nagK	4	3	3	-1	-1	0
nagZ	1	1	1	0	0	0
nanA	2	3	2	1	0	1
nanE	1	2	1	1	0	1
nanK	1	1	1	0	0	0

nanR	4	4	4	0	0	0
nanT	1	3	2	2	1	1
napA	2	3	2	1	0	1
napB	1	1	1	0	0	0
napC	1	1	1	0	0	0
napD	4	4	3	0	-1	1
napF	4	3	3	-1	-1	0
napG	1	2	1	1	0	1
napH	2	2	1	0	-1	1
narG	1	3	2	2	1	1
narH	1	2	2	1	1	0
narI	1	1	1	0	0	0
narJ	1	2	2	1	1	0
narK	3	3	3	0	0	0
narL	1	1	1	0	0	0
narP	3	3	2	0	-1	1
narQ	4	4	4	0	0	0
narU	2	3	2	1	0	1
narV	1	1	1	0	0	0
narW	1	1	1	0	0	0
narX	3	3	3	0	0	0
narY	1	1	1	0	0	0
narZ	1	1	1	0	0	0
ndh	4	4	3	0	-1	1
ndk	1	1	1	0	0	0
nei	3	2	2	-1	-1	0
nemA	1	1	1	0	0	0
nemR	4	4	3	0	-1	1
nepI	4	4	3	0	-1	1
nfeF	1	2	2	1	1	0
nfeR	1	1	1	0	0	0
nfi	2	2	2	0	0	0
nfo	2	2	2	0	0	0
nfrA	4	4	3	0	-1	1
nfrB	4	4	4	0	0	0
nfsA	3	3	2	0	-1	1
nfsB	1	1	1	0	0	0
nfuA	2	1	1	-1	-1	0
nhaA	4	3	2	-1	-2	1
nhaB	4	3	2	-1	-2	1
nhaR	4	4	2	0	-2	2
nhoA	3	3	3	0	0	0
nikA	4	4	4	0	0	0
nikB	3	4	3	1	0	1
nikC	3	3	3	0	0	0
nikD	3	3	2	0	-1	1
nikE	3	4	3	1	0	1
nikR	4	4	3	0	-1	1
nimR	4	4	3	0	-1	1
nimT	3	3	3	0	0	0
nirB	4	4	3	0	-1	1

nirC	4	4	3	0	-1	1
nirD	4	3	3	-1	-1	0
nlpA	4	4	3	0	-1	1
nlpC	4	4	3	0	-1	1
nlpD	3	2	1	-1	-2	1
nlpE	4	3	2	-1	-2	1
nlpI	4	3	3	-1	-1	0
nnr	3	1	1	-2	-2	0
norR	4	4	3	0	-1	1
norV	4	4	3	0	-1	1
norW	4	4	3	0	-1	1
npr	1	1	1	0	0	0
nrdA	4	4	3	0	-1	1
nrdB	3	3	3	0	0	0
nrdD	1	1	1	0	0	0
nrdE	4	4	3	0	-1	1
nrdF	3	3	3	0	0	0
nrdG	1	1	1	0	0	0
nrdH	4	3	2	-1	-2	1
nrdI	4	4	3	0	-1	1
nrdR	4	4	3	0	-1	1
nrfA	4	3	3	-1	-1	0
nrfB	4	3	3	-1	-1	0
nrfC	3	3	2	0	-1	1
nrfD	3	3	2	0	-1	1
nrfE	3	3	2	0	-1	1
nrfF	4	4	3	0	-1	1
nrfG	4	4	4	0	0	0
nsrR	4	4	3	0	-1	1
nth	1	1	1	0	0	0
nudB	1	2	2	1	1	0
nudC	3	3	2	0	-1	1
nudE	4	4	4	0	0	0
nudF	4	4	3	0	-1	1
nudG	4	4	3	0	-1	1
nudI	2	1	1	-1	-1	0
nudJ	4	4	4	0	0	0
nudK	2	3	2	1	0	1
nudL	4	4	3	0	-1	1
nuoA	3	3	3	0	0	0
nuoB	3	3	3	0	0	0
nuoC	2	3	2	1	0	1
nuoE	1	2	1	1	0	1
nuoF	1	1	1	0	0	0
nuoG	1	1	1	0	0	0
nuoH	1	1	1	0	0	0
nuoI	1	1	1	0	0	0
nuoJ	1	1	1	0	0	0
nuoK	1	1	1	0	0	0
nuoL	1	1	1	0	0	0
nuoM	1	1	1	0	0	0

nuoN	1	1	1	0	0	0
nupC	2	2	2	0	0	0
nupG	3	4	3	1	0	1
nupX	4	4	3	0	-1	1
nusA	3	3	2	0	-1	1
nusB	1	2	1	1	0	1
nusG	3	4	3	1	0	1
obgE	4	4	3	0	-1	1
ogt	4	4	4	0	0	0
ompA	1	1	1	0	0	0
ompC	1	1	1	0	0	0
ompF	1	1	1	0	0	0
ompG	2	2	2	0	0	0
ompL	2	2	2	0	0	0
ompN	1	2	1	1	0	1
ompR	4	3	3	-1	-1	0
ompW	4	3	2	-1	-2	1
ompX	4	2	1	-2	-3	1
omrA	4	4	4	0	0	0
omrB	4	4	4	0	0	0
opgB	4	4	4	0	0	0
opgC	4	4	4	0	0	0
opgD	3	3	3	0	0	0
opgE	4	4	4	0	0	0
opgG	3	3	3	0	0	0
opgH	3	3	3	0	0	0
oppA	1	1	1	0	0	0
oppB	1	2	1	1	0	1
oppC	1	2	1	1	0	1
oppD	1	2	1	1	0	1
oppF	1	1	1	0	0	0
orn	2	2	1	0	-1	1
ortT	3	4	4	1	1	0
osmB	1	1	1	0	0	0
osmC	1	1	1	0	0	0
osmE	1	1	1	0	0	0
osmF	2	2	2	0	0	0
osmY	1	1	1	0	0	0
otsA	1	2	1	1	0	1
otsB	3	4	3	1	0	1
oxc	4	3	2	-1	-2	1
oxyR	1	1	1	0	0	0
oxyS	4	4	3	0	-1	1
paaB	3	2	2	-1	-1	0
paaC	2	3	2	1	0	1
paaD	2	3	3	1	1	0
paaE	3	3	3	0	0	0
paaF	3	3	3	0	0	0
paaG	1	2	2	1	1	0
paaH	2	2	2	0	0	0
paaI	1	2	2	1	1	0

paaJ	3	3	2	0	-1	1
paaK	3	3	2	0	-1	1
paaX	4	4	3	0	-1	1
paaY	3	3	3	0	0	0
pabA	4	4	3	0	-1	1
pabB	4	4	3	0	-1	1
pabC	3	2	2	-1	-1	0
pagP	4	4	3	0	-1	1
pal	1	1	1	0	0	0
panB	4	4	3	0	-1	1
panC	2	3	2	1	0	1
panD	1	3	2	2	1	1
panE	3	3	2	0	-1	1
panF	3	3	3	0	0	0
panZ	4	4	3	0	-1	1
paoA	3	4	3	1	0	1
paoB	1	3	2	2	1	1
paoC	1	3	2	2	1	1
paoD	2	3	2	1	0	1
parC	3	4	3	1	0	1
parE	4	4	3	0	-1	1
patA	1	3	2	2	1	1
patD	1	1	1	0	0	0
patZ	4	4	3	0	-1	1
pbpC	2	3	2	1	0	1
pbpG	4	4	3	0	-1	1
pck	1	1	1	0	0	0
pcm	3	4	3	1	0	1
pcnB	4	4	4	0	0	0
pdeA	4	4	4	0	0	0
pdeB	4	4	4	0	0	0
pdeC	4	4	4	0	0	0
pdeD	3	4	3	1	0	1
pdeF	4	4	3	0	-1	1
pdeG	4	4	4	0	0	0
pdeH	4	3	3	-1	-1	0
pdeI	3	3	3	0	0	0
pdeK	4	4	3	0	-1	1
pdeL	4	4	3	0	-1	1
pdeN	4	4	4	0	0	0
pdeR	4	4	4	0	0	0
pdhR	4	4	4	0	0	0
pdxA	4	4	3	0	-1	1
pdxB	1	2	2	1	1	0
pdxH	2	3	3	1	1	0
pdxI	1	2	2	1	1	0
pdxJ	1	1	1	0	0	0
pdxK	1	1	1	0	0	0
pdxY	1	1	1	0	0	0
pepA	3	3	2	0	-1	1
pepB	1	1	1	0	0	0

pepD	1	1	1	0	0	0
pepE	1	1	1	0	0	0
pepN	3	3	2	0	-1	1
pepP	3	3	2	0	-1	1
pepQ	2	1	1	-1	-1	0
pepT	1	2	1	1	0	1
pfkA	2	2	2	0	0	0
pfkB	1	1	1	0	0	0
pflA	1	3	2	2	1	1
pflB	1	1	1	0	0	0
pflC	4	4	3	0	-1	1
pflD	3	3	2	0	-1	1
pgaA	4	4	3	0	-1	1
pgaB	4	3	3	-1	-1	0
pgaC	1	1	1	0	0	0
pgaD	1	1	1	0	0	0
pgeF	4	4	3	0	-1	1
pgi	4	4	3	0	-1	1
pgk	2	3	2	1	0	1
pgl	1	1	1	0	0	0
pgm	1	1	1	0	0	0
pgpA	3	4	3	1	0	1
pgpB	4	4	3	0	-1	1
pgpC	4	4	3	0	-1	1
pgrR	4	4	4	0	0	0
pgsA	4	4	4	0	0	0
pheA	2	3	3	1	1	0
pheL	4	4	3	0	-1	1
pheM	4	4	4	0	0	0
pheP	4	3	3	-1	-1	0
pheS	1	2	2	1	1	0
pheT	1	1	1	0	0	0
pheU	1	2	2	1	1	0
pheV	4	4	4	0	0	0
phnC	4	3	3	-1	-1	0
phnD	3	3	3	0	0	0
phnF	2	2	3	0	1	-1
phnG	1	3	3	2	2	0
phnH	3	3	3	0	0	0
phnI	1	1	2	0	1	-1
phnJ	1	2	1	1	0	1
phnK	3	4	3	1	0	1
phnL	4	3	2	-1	-2	1
phnM	1	2	2	1	1	0
phnN	3	3	3	0	0	0
phnO	3	4	3	1	0	1
phnP	4	4	3	0	-1	1
phoA	3	3	2	0	-1	1
phoB	3	4	2	1	-1	2
phoE	4	4	4	0	0	0
phoH	1	1	1	0	0	0

phoP	3	4	3	1	0	1
phoQ	4	4	3	0	-1	1
phoR	3	4	3	1	0	1
phoU	1	1	1	0	0	0
php	1	1	1	0	0	0
phr	3	3	3	0	0	0
pinQ	4	4	4	0	0	0
pinR	4	4	3	0	-1	1
pitA	3	4	3	1	0	1
pitB	4	3	2	-1	-2	1
plaP	1	3	3	2	2	0
pldA	4	4	4	0	0	0
pldB	4	4	4	0	0	0
pliG	1	1	1	0	0	0
plsB	3	4	3	1	0	1
plsC	4	4	4	0	0	0
plsX	2	2	2	0	0	0
plsY	4	4	4	0	0	0
pmbA	3	3	3	0	0	0
pmrD	4	3	3	-1	-1	0
pmrR	4	4	3	0	-1	1
pncA	2	2	2	0	0	0
pncB	4	4	4	0	0	0
pncC	2	3	2	1	0	1
pnp	3	1	1	-2	-2	0
pntA	1	1	1	0	0	0
pntB	1	1	1	0	0	0
pnuC	4	3	2	-1	-2	1
polA	3	3	3	0	0	0
polB	4	4	3	0	-1	1
potA	3	4	3	1	0	1
potB	3	3	3	0	0	0
potC	2	3	2	1	0	1
potD	1	1	1	0	0	0
potE	4	3	2	-1	-2	1
potF	1	3	1	2	0	2
potG	1	3	1	2	0	2
potH	1	3	2	2	1	1
potI	2	3	2	1	0	1
poxB	2	1	1	-1	-1	0
ppa	2	2	2	0	0	0
ppc	2	3	2	1	0	1
ppdA	3	4	3	1	0	1
ppdB	4	3	2	-1	-2	1
ppdC	4	4	3	0	-1	1
ppdD	4	4	3	0	-1	1
pphA	4	4	4	0	0	0
pphB	4	4	4	0	0	0
pphC	4	4	3	0	-1	1
ppiA	3	4	4	1	1	0
ppiB	1	2	2	1	1	0

ppiC	3	3	2	0	-1	1
ppiD	2	3	2	1	0	1
ppk	4	4	4	0	0	0
ppnN	2	2	1	0	-1	1
ppnP	1	1	1	0	0	0
pppA	4	3	3	-1	-1	0
ppsA	1	1	1	0	0	0
ppsR	1	2	2	1	1	0
pptA	2	1	1	-1	-1	0
ppx	3	4	3	1	0	1
pqiA	2	3	2	1	0	1
pqiB	1	1	1	0	0	0
pqiC	1	1	1	0	0	0
pqqL	1	1	1	0	0	0
prc	4	4	4	0	0	0
preA	1	2	1	1	0	1
preT	1	2	1	1	0	1
prfA	4	4	4	0	0	0
prfB	2	2	2	0	0	0
prfC	3	4	4	1	1	0
priA	4	4	3	0	-1	1
priB	3	2	2	-1	-1	0
priC	4	4	2	0	-2	2
prkB	4	4	3	0	-1	1
prlC	3	3	2	0	-1	1
prlF	4	3	3	-1	-1	0
prmA	2	2	2	0	0	0
prmB	3	3	3	0	0	0
prmC	4	4	4	0	0	0
proA	2	3	2	1	0	1
proB	4	4	3	0	-1	1
proC	2	3	2	1	0	1
proK	4	4	3	0	-1	1
proL	4	4	4	0	0	0
proM	4	4	3	0	-1	1
proP	1	2	1	1	0	1
proQ	3	3	3	0	0	0
proS	1	2	2	1	1	0
proV	4	4	3	0	-1	1
proW	4	3	2	-1	-2	1
proX	1	2	2	1	1	0
proY	4	4	3	0	-1	1
prpB	4	3	3	-1	-1	0
prpC	3	3	2	0	-1	1
prpD	1	1	2	0	1	-1
prpE	2	2	2	0	0	0
prpR	4	4	3	0	-1	1
prs	1	1	1	0	0	0
psd	3	3	3	0	0	0
psiE	4	4	1	0	-3	3
psiF	1	2	2	1	1	0

pspA	1	1	1	0	0	0
pspB	1	1	1	0	0	0
pspC	1	1	1	0	0	0
pspD	1	1	1	0	0	0
pspE	1	1	1	0	0	0
pspF	4	4	4	0	0	0
pspG	1	1	1	0	0	0
pssA	3	4	3	1	0	1
pstA	2	3	2	1	0	1
pstB	2	2	1	0	-1	1
pstC	2	2	1	0	-1	1
pstS	1	1	1	0	0	0
psuG	1	1	1	0	0	0
psuK	3	2	2	-1	-1	0
psuT	3	3	3	0	0	0
pta	1	1	1	0	0	0
pth	1	3	2	2	1	1
ptrA	3	3	3	0	0	0
ptrB	3	4	3	1	0	1
ptsA	3	3	3	0	0	0
ptsG	1	1	1	0	0	0
ptsH	1	1	1	0	0	0
ptsI	1	1	1	0	0	0
ptsN	1	1	1	0	0	0
ptsP	4	4	4	0	0	0
purA	1	1	1	0	0	0
purB	1	2	1	1	0	1
purC	1	1	1	0	0	0
purD	1	2	2	1	1	0
purE	3	3	3	0	0	0
purF	1	2	1	1	0	1
purH	1	2	2	1	1	0
purK	2	3	3	1	1	0
purL	2	2	2	0	0	0
purM	2	3	3	1	1	0
purN	2	3	3	1	1	0
purR	4	3	3	-1	-1	0
purT	1	2	2	1	1	0
purU	2	3	3	1	1	0
putA	1	3	1	2	0	2
putP	3	4	2	1	-1	2
puuA	4	4	3	0	-1	1
puuB	1	1	1	0	0	0
puuC	1	1	1	0	0	0
puuD	3	3	2	0	-1	1
puuE	1	2	2	1	1	0
puuP	4	4	4	0	0	0
puuR	2	1	1	-1	-1	0
pxpA	2	2	1	0	-1	1
pxpB	1	1	1	0	0	0
pxpC	1	1	1	0	0	0

pykA	1	1	1	0	0	0
pykF	2	3	3	1	1	0
pyrB	1	1	1	0	0	0
pyrC	1	1	1	0	0	0
pyrD	1	2	2	1	1	0
pyrE	1	1	1	0	0	0
pyrF	2	3	3	1	1	0
pyrG	1	2	2	1	1	0
pyrH	1	1	1	0	0	0
pyrI	1	1	1	0	0	0
pyrL	1	1	1	0	0	0
qmcA	4	3	3	-1	-1	0
qorA	1	3	2	2	1	1
qorB	2	2	2	0	0	0
qseB	4	4	3	0	-1	1
qseC	4	4	3	0	-1	1
qseG	4	4	4	0	0	0
queA	4	4	4	0	0	0
queC	2	3	2	1	0	1
queD	4	4	4	0	0	0
queE	4	4	4	0	0	0
queF	3	3	3	0	0	0
queG	4	4	4	0	0	0
radA	4	4	3	0	-1	1
radD	4	4	4	0	0	0
raiA	1	1	1	0	0	0
rapA	3	4	3	1	0	1
rapZ	1	1	1	0	0	0
rarA	1	1	1	0	0	0
rarD	3	3	3	0	0	0
ratA	3	4	3	1	0	1
ravA	4	4	4	0	0	0
rbbA	4	4	3	0	-1	1
rbfA	2	1	1	-1	-1	0
rbn	4	4	3	0	-1	1
rbsA	4	4	4	0	0	0
rbsB	1	1	1	0	0	0
rbsC	3	4	3	1	0	1
rbsD	4	2	2	-2	-2	0
rbsK	3	4	3	1	0	1
rbsR	4	4	3	0	-1	1
rcdA	4	4	4	0	0	0
rclA	1	1	1	0	0	0
rclB	1	3	2	2	1	1
rclC	2	3	2	1	0	1
rclR	4	4	3	0	-1	1
rcnA	4	4	3	0	-1	1
rcnB	3	2	1	-1	-2	1
rcnR	4	4	3	0	-1	1
rcsA	4	3	3	-1	-1	0
rcsB	3	3	2	0	-1	1

rcsC	4	4	4	0	0	0
rcsD	4	4	3	0	-1	1
rcsF	3	3	3	0	0	0
rdgB	1	2	2	1	1	0
rdgC	3	4	3	1	0	1
rdlA	1	1	1	0	0	0
rdlB	1	1	1	0	0	0
rdlC	1	1	1	0	0	0
rdlD	1	1	1	0	0	0
recA	1	2	2	1	1	0
recB	1	2	2	1	1	0
recC	4	1	1	-3	-3	0
recD	1	1	1	0	0	0
recF	3	4	3	1	0	1
recG	4	4	3	0	-1	1
recJ	2	3	2	1	0	1
recN	4	4	4	0	0	0
recO	3	4	3	1	0	1
recQ	4	4	4	0	0	0
recR	1	1	1	0	0	0
recX	3	3	3	0	0	0
relA	4	4	3	0	-1	1
relB	1	1	1	0	0	0
relE	1	1	1	0	0	0
rem	4	3	3	-1	-1	0
rep	4	4	4	0	0	0
rfaD	4	4	3	0	-1	1
rfaE	1	2	2	1	1	0
rfaH	3	4	3	1	0	1
rfbA	3	3	2	0	-1	1
rfbB	3	3	3	0	0	0
rfbD	3	3	3	0	0	0
rfe	4	4	4	0	0	0
rffC	1	1	1	0	0	0
rffG	1	1	1	0	0	0
rffH	1	1	1	0	0	0
rffM	1	1	1	0	0	0
rffT	1	1	1	0	0	0
rhaA	2	2	2	0	0	0
rhaB	4	4	4	0	0	0
rhaD	1	2	1	1	0	1
rhaM	1	2	1	1	0	1
rhaR	4	4	3	0	-1	1
rhaS	4	4	4	0	0	0
rhaT	4	2	2	-2	-2	0
rhlB	4	4	4	0	0	0
rhlE	4	4	4	0	0	0
rhmD	1	1	1	0	0	0
rho	3	3	3	0	0	0
rhoL	4	4	4	0	0	0
rhsA	4	3	3	-1	-1	0

rhsB	4	3	3	-1	-1	0
rhsC	4	3	3	-1	-1	0
rhsD	2	2	2	0	0	0
rhtA	4	3	3	-1	-1	0
rhtB	4	4	3	0	-1	1
rhtC	4	4	4	0	0	0
ribA	3	3	3	0	0	0
ribB	4	4	3	0	-1	1
ribC	3	3	2	0	-1	1
ribD	4	4	3	0	-1	1
ribE	1	2	2	1	1	0
ribF	4	4	4	0	0	0
ridA	1	1	1	0	0	0
rihA	1	1	1	0	0	0
rihB	2	2	2	0	0	0
rihC	1	1	1	0	0	0
rimI	4	4	4	0	0	0
rimJ	3	3	2	0	-1	1
rimK	3	3	2	0	-1	1
rimL	4	4	3	0	-1	1
rimM	3	2	2	-1	-1	0
rimO	3	4	4	1	1	0
rimP	3	4	3	1	0	1
rlhA	4	4	4	0	0	0
rlmA	2	3	3	1	1	0
rlmB	1	1	1	0	0	0
rlmC	4	4	4	0	0	0
rlmD	4	4	3	0	-1	1
rlmE	4	4	3	0	-1	1
rlmF	3	4	3	1	0	1
rlmG	1	3	3	2	2	0
rlmH	1	3	2	2	1	1
rlmI	3	4	3	1	0	1
rlmJ	4	4	4	0	0	0
rlmL	4	4	4	0	0	0
rlmM	4	4	3	0	-1	1
rlmN	1	2	1	1	0	1
rlpA	1	1	1	0	0	0
rluA	2	3	2	1	0	1
rluB	4	4	3	0	-1	1
rluC	4	4	4	0	0	0
rluD	4	4	3	0	-1	1
rluE	4	4	4	0	0	0
rluF	3	3	2	0	-1	1
rmf	1	1	1	0	0	0
rmuC	4	4	4	0	0	0
rna	4	4	4	0	0	0
rnb	2	3	3	1	1	0
rnc	4	4	4	0	0	0
rnd	4	4	3	0	-1	1
rne	4	4	3	0	-1	1

rng	3	4	3	1	0	1
rnhA	4	4	3	0	-1	1
rnhB	1	1	1	0	0	0
rnk	1	1	1	0	0	0
rnpA	4	4	3	0	-1	1
rnpB	1	1	1	0	0	0
rnr	3	3	1	0	-2	2
rnt	4	3	3	-1	-1	0
rob	3	4	3	1	0	1
rodZ	4	4	4	0	0	0
rof	1	2	1	1	0	1
roxA	3	4	3	1	0	1
rpe	1	1	1	0	0	0
rph	3	4	3	1	0	1
rpiA	1	1	1	0	0	0
rpiB	4	3	2	-1	-2	1
rplA	3	3	2	0	-1	1
rplB	1	1	1	0	0	0
rplC	2	1	1	-1	-1	0
rplD	2	1	1	-1	-1	0
rplE	3	1	1	-2	-2	0
rplF	3	3	2	0	-1	1
rplI	3	2	2	-1	-1	0
rplJ	1	1	1	0	0	0
rplK	3	3	3	0	0	0
rplL	2	1	1	-1	-1	0
rplM	1	1	1	0	0	0
rplN	3	1	1	-2	-2	0
rplO	2	1	1	-1	-1	0
rplP	1	1	1	0	0	0
rplQ	1	1	1	0	0	0
rplR	3	2	2	-1	-1	0
rplS	3	2	2	-1	-1	0
rplT	2	1	1	-1	-1	0
rplU	3	4	3	1	0	1
rplV	1	1	1	0	0	0
rplW	2	1	1	-1	-1	0
rplX	3	1	1	-2	-2	0
rplY	1	1	1	0	0	0
rpmA	3	3	3	0	0	0
rpmB	2	2	2	0	0	0
rpmC	1	1	1	0	0	0
rpmD	2	1	1	-1	-1	0
rpmE	1	1	1	0	0	0
rpmF	1	1	1	0	0	0
rpmG	1	1	1	0	0	0
rpmH	3	2	2	-1	-1	0
rpmI	2	1	1	-1	-1	0
rpmJ	2	1	1	-1	-1	0
rpnA	4	3	3	-1	-1	0
rpnB	4	4	3	0	-1	1

rpnC	4	4	3	0	-1	1
rpnE	3	3	3	0	0	0
rpoA	1	1	1	0	0	0
rpoB	1	1	1	0	0	0
rpoC	1	1	1	0	0	0
rpoD	1	1	1	0	0	0
rpoE	4	4	3	0	-1	1
rpoH	4	4	2	0	-2	2
rpoN	2	2	2	0	0	0
rpoS	3	3	1	0	-2	2
rpoZ	2	2	2	0	0	0
rppH	4	4	4	0	0	0
rprA	3	3	3	0	0	0
rpsA	1	1	1	0	0	0
rpsB	1	1	1	0	0	0
rpsC	1	1	1	0	0	0
rpsD	1	1	1	0	0	0
rpsE	3	2	2	-1	-1	0
rpsF	3	2	2	-1	-1	0
rpsG	1	1	1	0	0	0
rpsH	3	3	3	0	0	0
rpsI	1	1	1	0	0	0
rpsJ	2	1	1	-1	-1	0
rpsK	2	1	1	-1	-1	0
rpsL	2	1	1	-1	-1	0
rpsM	2	1	1	-1	-1	0
rpsN	4	3	3	-1	-1	0
rpsO	4	3	3	-1	-1	0
rpsP	3	2	2	-1	-1	0
rpsQ	1	1	1	0	0	0
rpsR	3	2	2	-1	-1	0
rpsS	1	1	1	0	0	0
rpsT	3	3	2	0	-1	1
rpsU	3	3	3	0	0	0
rraA	3	3	2	0	-1	1
rraB	2	2	1	0	-1	1
rrfA	1	3	3	2	2	0
rrfB	1	2	3	1	2	-1
rrfC	1	3	3	2	2	0
rrfD	1	3	3	2	2	0
rrfE	1	3	3	2	2	0
rrfF	3	4	4	1	1	0
rrfG	1	1	3	0	2	-2
rrfH	1	3	3	2	2	0
rrlA	1	1	1	0	0	0
rrlB	1	1	1	0	0	0
rrlC	1	1	1	0	0	0
rrlD	1	1	1	0	0	0
rrlE	1	1	1	0	0	0
rrlG	1	1	1	0	0	0
rrlH	1	1	1	0	0	0

rrrQ	4	4	3	0	-1	1
rrsA	1	1	1	0	0	0
rrsB	1	1	1	0	0	0
rrsC	1	1	1	0	0	0
rrsD	1	1	1	0	0	0
rrsE	1	1	1	0	0	0
rrsG	1	1	1	0	0	0
rrsH	1	1	1	0	0	0
rsd	4	4	2	0	-2	2
rseA	4	4	3	0	-1	1
rseB	4	4	4	0	0	0
rseC	4	4	4	0	0	0
rseD	4	4	4	0	0	0
rseP	4	4	3	0	-1	1
rsfS	2	3	2	1	0	1
rsgA	4	3	3	-1	-1	0
rsmA	4	4	3	0	-1	1
rsmB	4	4	3	0	-1	1
rsmC	4	4	4	0	0	0
rsmD	4	4	4	0	0	0
rsmE	4	4	3	0	-1	1
rsmF	3	3	3	0	0	0
rsmG	3	4	4	1	1	0
rsmH	4	4	3	0	-1	1
rsmI	4	4	4	0	0	0
rsmJ	2	3	2	1	0	1
rspA	4	4	4	0	0	0
rspB	4	4	3	0	-1	1
rssA	4	4	4	0	0	0
rssB	4	4	4	0	0	0
rstA	4	4	4	0	0	0
rstB	4	4	4	0	0	0
rsuA	4	4	4	0	0	0
rsxA	4	4	4	0	0	0
rsxB	4	4	3	0	-1	1
rsxC	3	4	3	1	0	1
rsxD	2	3	3	1	1	0
rsxE	1	2	1	1	0	1
rsxG	1	2	2	1	1	0
rtcA	4	4	3	0	-1	1
rtcB	4	3	1	-1	-3	2
rtcR	4	4	3	0	-1	1
rttR	4	4	4	0	0	0
rutA	3	3	1	0	-2	2
rutB	4	4	3	0	-1	1
rutC	4	4	4	0	0	0
rutD	4	3	3	-1	-1	0
rutE	4	3	3	-1	-1	0
rutF	3	3	2	0	-1	1
rutG	3	3	3	0	0	0
rutR	4	3	2	-1	-2	1

ruvA	4	4	3	0	-1	1
ruvB	3	3	3	0	0	0
ruvC	1	1	1	0	0	0
rybA	4	4	4	0	0	0
rybB	4	4	4	0	0	0
rydB	4	4	4	0	0	0
rydC	3	3	3	0	0	0
ryeA	2	3	2	1	0	1
ryfA	1	1	1	0	0	0
ryfD	1	2	1	1	0	1
ryhB	4	4	4	0	0	0
ryjA	4	2	2	-2	-2	0
rzoQ	4	4	3	0	-1	1
rzpQ	4	4	3	0	-1	1
sad	2	3	2	1	0	1
sanA	4	4	4	0	0	0
sapA	4	4	4	0	0	0
sapB	4	4	4	0	0	0
sapC	3	4	3	1	0	1
sapD	3	4	3	1	0	1
sapF	1	2	2	1	1	0
satP	3	3	2	0	-1	1
sbcB	4	4	4	0	0	0
sbcC	4	4	3	0	-1	1
sbcD	4	4	3	0	-1	1
sbmA	4	4	3	0	-1	1
sbmC	2	2	1	0	-1	1
sbp	4	4	3	0	-1	1
scpA	4	3	2	-1	-2	1
scpB	3	3	2	0	-1	1
scpC	4	3	2	-1	-2	1
sdaA	1	2	2	1	1	0
sdaB	3	3	3	0	0	0
sdaC	3	4	3	1	0	1
sdhA	2	2	1	0	-1	1
sdhB	1	2	1	1	0	1
sdhC	4	4	3	0	-1	1
sdhD	4	3	3	-1	-1	0
sdhE	4	3	3	-1	-1	0
sdiA	4	4	4	0	0	0
sdsN	3	4	3	1	0	1
sdsR	2	3	2	1	0	1
secA	3	3	3	0	0	0
secB	1	1	1	0	0	0
secD	4	4	3	0	-1	1
secE	4	4	4	0	0	0
secF	4	4	3	0	-1	1
secG	4	4	4	0	0	0
secM	4	4	4	0	0	0
secY	2	1	1	-1	-1	0
selA	3	4	3	1	0	1

selB	2	3	2	1	0	1
selC	4	4	4	0	0	0
selD	3	2	2	-1	-1	0
selU	4	4	4	0	0	0
seqA	3	4	3	1	0	1
serA	2	3	2	1	0	1
serB	4	4	3	0	-1	1
serC	1	2	2	1	1	0
serS	1	1	1	0	0	0
serT	4	4	4	0	0	0
serU	4	4	4	0	0	0
serV	4	4	3	0	-1	1
serW	3	4	3	1	0	1
serX	1	3	2	2	1	1
setA	4	4	4	0	0	0
setB	4	4	3	0	-1	1
setC	4	4	3	0	-1	1
sfmA	1	2	1	1	0	1
sfmC	4	3	2	-1	-2	1
sfmD	4	4	3	0	-1	1
sfmF	3	2	2	-1	-1	0
sfmH	4	3	2	-1	-2	1
sfsA	4	4	3	0	-1	1
sfsB	4	4	4	0	0	0
sgbE	1	1	1	0	0	0
sgbH	1	1	1	0	0	0
sgbU	1	1	1	0	0	0
sgrR	4	4	3	0	-1	1
sgrS	1	1	1	0	0	0
sgrT	1	1	1	0	0	0
shiA	4	4	3	0	-1	1
shoB	1	1	1	0	0	0
sibA	4	3	3	-1	-1	0
sibB	4	4	4	0	0	0
sibC	2	2	3	0	1	-1
sibD	4	1	1	-3	-3	0
sibE	2	3	3	1	1	0
sixA	3	3	2	0	-1	1
skp	1	1	1	0	0	0
slmA	2	3	3	1	1	0
slp	2	3	3	1	1	0
slt	4	4	3	0	-1	1
slyA	2	3	2	1	0	1
slyB	3	3	3	0	0	0
slyD	1	1	1	0	0	0
slyX	1	1	1	0	0	0
smf	4	4	3	0	-1	1
smg	4	4	3	0	-1	1
smpB	3	3	3	0	0	0
smrA	4	4	4	0	0	0
smrB	4	4	4	0	0	0

sodA	1	1	1	0	0	0
sodB	1	1	1	0	0	0
sodC	2	3	2	1	0	1
sohB	2	2	2	0	0	0
sokB	1	1	1	0	0	0
sokC	4	4	3	0	-1	1
sokE	4	4	3	0	-1	1
sokX	4	4	4	0	0	0
solA	1	1	1	0	0	0
soxR	4	3	3	-1	-1	0
soxS	3	4	2	1	-1	2
speA	1	1	1	0	0	0
speB	1	1	1	0	0	0
speC	2	4	3	2	1	1
speD	1	1	1	0	0	0
speE	1	1	1	0	0	0
speF	4	3	3	-1	-1	0
speG	2	1	1	-1	-1	0
spf	2	3	2	1	0	1
spoT	4	4	3	0	-1	1
sppA	4	4	3	0	-1	1
spy	1	1	1	0	0	0
sra	1	1	1	0	0	0
sraB	2	1	1	-1	-1	0
sraG	4	1	3	-3	-1	-2
srkA	3	4	3	1	0	1
srlA	1	1	1	0	0	0
srlB	1	1	1	0	0	0
srlD	1	1	1	0	0	0
srlE	1	1	1	0	0	0
srlR	2	3	3	1	1	0
srmB	2	3	3	1	1	0
ssb	1	2	2	1	1	0
sseA	1	2	1	1	0	1
sseB	1	1	1	0	0	0
ssnA	1	3	3	2	2	0
sspA	2	3	2	1	0	1
sspB	2	2	2	0	0	0
ssrA	1	1	1	0	0	0
ssrS	1	1	1	0	0	0
sstT	4	4	3	0	-1	1
ssuA	3	3	2	0	-1	1
ssuB	4	4	3	0	-1	1
ssuC	2	2	2	0	0	0
ssuD	1	2	2	1	1	0
ssuE	3	4	3	1	0	1
sthA	1	2	1	1	0	1
stpA	2	2	1	0	-1	1
sucA	1	1	1	0	0	0
sucB	1	1	1	0	0	0
sucC	1	1	1	0	0	0

sucD	1	1	1	0	0	0
sufA	1	2	1	1	0	1
sufB	1	2	1	1	0	1
sufC	1	2	1	1	0	1
sufD	1	1	1	0	0	0
sufE	1	1	1	0	0	0
sufS	1	1	1	0	0	0
suhB	2	3	3	1	1	0
sulA	4	4	2	0	-2	2
surA	3	3	2	0	-1	1
sutR	4	4	4	0	0	0
sxy	3	4	3	1	0	1
syd	4	4	4	0	0	0
tabA	2	3	2	1	0	1
tadA	4	4	3	0	-1	1
tag	3	3	2	0	-1	1
talA	1	2	1	1	0	1
talB	1	1	1	0	0	0
tam	4	3	3	-1	-1	0
tamA	4	4	4	0	0	0
tamB	4	4	3	0	-1	1
tap	2	2	2	0	0	0
tar	4	3	3	-1	-1	0
tas	1	1	1	0	0	0
tatA	3	3	2	0	-1	1
tatB	3	3	2	0	-1	1
tatC	3	3	3	0	0	0
tatD	3	3	3	0	0	0
tatE	1	1	1	0	0	0
tauA	4	3	2	-1	-2	1
tauB	4	4	3	0	-1	1
tauC	4	4	3	0	-1	1
tauD	4	4	4	0	0	0
tcdA	3	3	3	0	0	0
tcyJ	1	2	1	1	0	1
tcyL	2	3	3	1	1	0
tcyN	1	3	2	2	1	1
tcyP	3	4	3	1	0	1
tdcA	4	4	4	0	0	0
tdcB	1	1	1	0	0	0
tdcC	1	2	1	1	0	1
tdcD	1	1	1	0	0	0
tdcE	1	1	1	0	0	0
tdcF	1	1	1	0	0	0
tdcG	1	1	1	0	0	0
tdcR	4	4	4	0	0	0
tdh	1	1	1	0	0	0
tdk	4	4	4	0	0	0
tehA	4	4	3	0	-1	1
tehB	1	1	1	0	0	0
tesA	3	3	3	0	0	0

tesB	2	3	2	1	0	1
tfaQ	4	4	3	0	-1	1
tfaR	4	4	3	0	-1	1
tff	1	1	1	0	0	0
tgt	2	2	2	0	0	0
thiB	4	4	4	0	0	0
thiC	3	3	2	0	-1	1
thiD	4	4	3	0	-1	1
thiE	3	2	1	-1	-2	1
thiF	2	1	1	-1	-1	0
thiG	2	1	1	-1	-1	0
thiH	1	1	1	0	0	0
thiI	3	3	3	0	0	0
thiK	2	2	2	0	0	0
thiL	3	4	3	1	0	1
thiM	4	4	3	0	-1	1
thiP	4	4	3	0	-1	1
thiQ	4	4	3	0	-1	1
thiS	2	1	1	-1	-1	0
thpR	4	4	4	0	0	0
thrA	2	3	2	1	0	1
thrB	3	2	2	-1	-1	0
thrC	2	2	2	0	0	0
thrL	4	4	3	0	-1	1
thrS	1	1	1	0	0	0
thrT	4	4	4	0	0	0
thrU	4	4	4	0	0	0
thrV	4	4	4	0	0	0
thrW	4	4	4	0	0	0
thyA	1	2	1	1	0	1
tig	1	1	1	0	0	0
tilS	3	2	2	-1	-1	0
tisB	1	1	1	0	0	0
tktA	1	1	1	0	0	0
tktB	1	1	1	0	0	0
tldD	1	1	1	0	0	0
tmcA	4	4	3	0	-1	1
tmk	3	3	3	0	0	0
tnaA	1	1	1	0	0	0
tnaB	3	4	2	1	-1	2
tnaC	1	1	1	0	0	0
tolA	4	4	3	0	-1	1
tolB	1	1	1	0	0	0
tolC	1	2	2	1	1	0
tolQ	4	4	3	0	-1	1
tolR	4	4	3	0	-1	1
tomB	4	4	3	0	-1	1
tonB	4	4	4	0	0	0
topA	2	3	2	1	0	1
topB	4	3	3	-1	-1	0
torA	3	3	3	0	0	0

torC	4	4	3	0	-1	1
torD	4	4	3	0	-1	1
torR	4	4	4	0	0	0
torS	4	4	4	0	0	0
torT	4	4	4	0	0	0
torY	4	3	2	-1	-2	1
torZ	1	1	1	0	0	0
tpiA	1	1	1	0	0	0
tpr	4	4	4	0	0	0
tpx	1	1	1	0	0	0
tqsA	4	4	3	0	-1	1
treA	1	2	1	1	0	1
treB	4	4	3	0	-1	1
treC	3	3	3	0	0	0
treF	3	4	3	1	0	1
treR	4	4	4	0	0	0
trg	4	4	4	0	0	0
trkA	2	3	2	1	0	1
trkH	4	4	3	0	-1	1
trmA	3	3	3	0	0	0
trmD	3	2	2	-1	-1	0
trmH	4	4	4	0	0	0
trmI	3	3	3	0	0	0
trmJ	4	4	4	0	0	0
trmL	3	3	2	0	-1	1
trmO	3	4	3	1	0	1
trpA	1	1	1	0	0	0
trpB	1	2	1	1	0	1
trpC	3	4	2	1	-1	2
trpD	4	4	3	0	-1	1
trpE	3	4	3	1	0	1
trpR	4	4	4	0	0	0
trpS	1	1	1	0	0	0
trpT	4	4	3	0	-1	1
truA	1	2	2	1	1	0
truB	2	1	1	-1	-1	0
truC	4	4	4	0	0	0
truD	3	2	2	-1	-1	0
trxA	2	2	2	0	0	0
trxB	1	1	1	0	0	0
trxC	4	4	3	0	-1	1
tsaB	4	4	4	0	0	0
tsaC	4	3	3	-1	-1	0
tsaD	3	4	3	1	0	1
tsaE	4	4	3	0	-1	1
tsf	1	1	1	0	0	0
tsgA	1	3	2	2	1	1
tsr	4	4	3	0	-1	1
tsx	2	2	1	0	-1	1
ttcA	4	4	4	0	0	0
ttdA	3	4	3	1	0	1

ttdB	2	3	3	1	1	0
ttdR	4	4	4	0	0	0
ttdT	4	4	4	0	0	0
tufA	1	1	1	0	0	0
tufB	1	1	1	0	0	0
tus	3	3	3	0	0	0
tusA	4	4	3	0	-1	1
tusB	4	4	4	0	0	0
tusC	4	4	4	0	0	0
tusD	4	4	4	0	0	0
tusE	3	4	3	1	0	1
tyrA	3	4	3	1	0	1
tyrB	1	1	1	0	0	0
tyrP	4	4	4	0	0	0
tyrR	4	4	3	0	-1	1
tyrS	1	1	1	0	0	0
tyrT	4	4	4	0	0	0
tyrU	4	4	4	0	0	0
tyrV	4	4	4	0	0	0
uacT	4	3	3	-1	-1	0
ubiA	4	4	3	0	-1	1
ubiB	4	4	3	0	-1	1
ubiC	4	4	4	0	0	0
ubiD	4	4	4	0	0	0
ubiE	4	4	3	0	-1	1
ubiF	3	3	3	0	0	0
ubiG	2	3	3	1	1	0
ubiH	3	3	3	0	0	0
ubiI	2	3	2	1	0	1
ubiJ	4	4	3	0	-1	1
ubiK	3	3	2	0	-1	1
ubiX	4	4	3	0	-1	1
ucpA	1	1	1	0	0	0
udk	3	3	2	0	-1	1
udp	1	1	1	0	0	0
ugd	1	1	1	0	0	0
ugpA	1	4	2	3	1	2
ugpB	1	4	2	3	1	2
ugpC	1	2	1	1	0	1
ugpE	1	3	2	2	1	1
ugpQ	1	2	1	1	0	1
uhpA	4	4	4	0	0	0
uhpB	4	4	4	0	0	0
uhpC	4	4	3	0	-1	1
uhpT	4	4	3	0	-1	1
uidA	1	3	3	2	2	0
uidB	2	3	3	1	1	0
uidC	2	3	2	1	0	1
uidR	4	4	3	0	-1	1
ulaA	2	3	2	1	0	1
ulaB	1	1	1	0	0	0

ulaC	1	1	1	0	0	0
ulaD	1	1	1	0	0	0
ulaE	1	1	1	0	0	0
ulaF	1	1	1	0	0	0
ulaG	2	3	2	1	0	1
ulaR	4	4	4	0	0	0
umpG	4	3	3	-1	-1	0
umpH	4	4	3	0	-1	1
umuC	4	4	3	0	-1	1
umuD	4	4	3	0	-1	1
ung	4	4	3	0	-1	1
uof	4	4	3	0	-1	1
upp	1	1	1	0	0	0
uraA	1	1	1	0	0	0
usg	2	2	2	0	0	0
ushA	2	2	2	0	0	0
uspA	1	1	1	0	0	0
uspB	4	4	2	0	-2	2
uspC	4	4	3	0	-1	1
uspD	1	1	1	0	0	0
uspE	1	1	1	0	0	0
uspF	1	1	1	0	0	0
uspG	1	1	1	0	0	0
uup	4	4	3	0	-1	1
uvrA	1	2	1	1	0	1
uvrB	3	4	3	1	0	1
uvrC	4	4	4	0	0	0
uvrD	3	4	3	1	0	1
uvrY	4	4	4	0	0	0
uxaA	2	3	2	1	0	1
uxaB	1	1	1	0	0	0
uxaC	1	3	2	2	1	1
uxuA	1	3	2	2	1	1
uxuB	1	2	1	1	0	1
uxuR	4	4	4	0	0	0
valS	2	3	3	1	1	0
valT	3	3	3	0	0	0
valU	4	4	3	0	-1	1
valV	4	4	4	0	0	0
valW	4	4	4	0	0	0
valX	4	4	3	0	-1	1
valY	4	4	3	0	-1	1
valZ	3	4	3	1	0	1
ves	4	4	4	0	0	0
viaA	4	4	4	0	0	0
vsr	4	4	4	0	0	0
waaA	4	4	4	0	0	0
waaC	2	1	2	-1	0	-1
waaF	4	3	3	-1	-1	0
waaG	3	4	3	1	0	1
waaH	4	3	3	-1	-1	0

waaQ	4	4	3	0	-1	1
wcaA	4	3	2	-1	-2	1
wcaB	3	3	2	0	-1	1
wcaC	3	3	2	0	-1	1
wcaD	2	1	1	-1	-1	0
wcaE	3	1	1	-2	-2	0
wcaF	3	2	1	-1	-2	1
wcaI	3	2	1	-1	-2	1
wcaJ	4	3	3	-1	-1	0
wcaK	2	3	2	1	0	1
wcaL	3	3	2	0	-1	1
wcaM	3	3	3	0	0	0
wecB	2	3	2	1	0	1
wecC	1	1	1	0	0	0
wecE	1	1	1	0	0	0
wecH	4	4	4	0	0	0
wrbA	1	1	1	0	0	0
wza	4	4	2	0	-2	2
wzb	3	4	2	1	-1	2
wzc	4	3	2	-1	-2	1
wzxC	3	3	2	0	-1	1
wzxE	1	1	1	0	0	0
wzyE	1	1	1	0	0	0
wzzB	2	3	3	1	1	0
wzzE	4	4	3	0	-1	1
xanP	4	4	4	0	0	0
xanQ	2	3	3	1	1	0
xapA	3	3	1	0	-2	2
xapB	4	4	3	0	-1	1
xapR	4	4	3	0	-1	1
xdhA	1	2	1	1	0	1
xdhB	1	2	1	1	0	1
xdhC	1	3	2	2	1	1
xdhD	1	2	2	1	1	0
xerC	4	4	3	0	-1	1
xerD	3	3	3	0	0	0
xseA	1	1	1	0	0	0
xseB	4	4	4	0	0	0
xthA	3	3	3	0	0	0
xylA	2	3	2	1	0	1
xylB	2	3	2	1	0	1
xylE	3	3	2	0	-1	1
xylF	1	2	2	1	1	0
xylG	3	3	3	0	0	0
xylH	2	4	3	2	1	1
xylR	3	4	3	1	0	1
xynR	4	4	4	0	0	0
yaaA	4	4	3	0	-1	1
yaaI	4	3	3	-1	-1	0
yaaJ	4	4	4	0	0	0
yaaU	3	2	2	-1	-1	0

yaaW	4	3	2	-1	-2	1
yaaX	4	3	2	-1	-2	1
yaaY	4	4	3	0	-1	1
yabI	4	4	4	0	0	0
yacC	4	4	4	0	0	0
yacG	4	4	3	0	-1	1
yacH	4	1	2	-3	-2	-1
yacL	2	2	2	0	0	0
yacM	4	4	4	0	0	0
yadC	4	4	3	0	-1	1
yadE	4	4	4	0	0	0
yadG	2	2	2	0	0	0
yadH	1	1	1	0	0	0
yadI	4	4	4	0	0	0
yadK	3	2	1	-1	-2	1
yadL	2	1	1	-1	-1	0
yadM	3	2	1	-1	-2	1
yadN	3	2	1	-1	-2	1
yadS	4	4	4	0	0	0
yadV	4	3	3	-1	-1	0
yadW	3	3	2	0	-1	1
yaeF	4	4	4	0	0	0
yaeH	2	2	1	0	-1	1
yaeI	4	4	3	0	-1	1
yaeP	1	2	1	1	0	1
yaeQ	4	4	3	0	-1	1
yaeR	1	1	1	0	0	0
yafC	3	3	3	0	0	0
yafD	4	4	3	0	-1	1
yafE	4	3	3	-1	-1	0
yafJ	4	4	3	0	-1	1
yafK	4	4	4	0	0	0
yafL	4	4	3	0	-1	1
yafP	4	4	4	0	0	0
yafQ	4	4	4	0	0	0
yafS	4	4	4	0	0	0
yafT	4	4	4	0	0	0
yafV	4	4	3	0	-1	1
yafW	3	3	2	0	-1	1
yafX	4	4	3	0	-1	1
yafY	1	1	1	0	0	0
yafZ	4	3	3	-1	-1	0
yagA	4	3	3	-1	-1	0
yagB	4	4	4	0	0	0
yagE	1	2	1	1	0	1
yagF	1	2	1	1	0	1
yagG	1	2	1	1	0	1
yagH	1	2	2	1	1	0
yagP	4	4	4	0	0	0
yagU	1	1	1	0	0	0
yahB	4	4	4	0	0	0

yahC	3	3	3	0	0	0
yahD	3	3	2	0	-1	1
yahE	4	3	3	-1	-1	0
yahF	3	2	1	-1	-2	1
yahG	3	3	2	0	-1	1
yahH	4	3	2	-1	-2	1
yahI	2	3	2	1	0	1
yahJ	2	3	2	1	0	1
yahK	1	2	1	1	0	1
yahL	4	4	3	0	-1	1
yahM	4	4	4	0	0	0
yahN	4	4	4	0	0	0
yahO	1	1	1	0	0	0
yaiA	3	4	3	1	0	1
yaiI	4	4	3	0	-1	1
yaiL	4	4	3	0	-1	1
yaiO	4	4	2	0	-2	2
yaiP	4	4	4	0	0	0
yaiS	4	3	2	-1	-2	1
yaiW	4	4	3	0	-1	1
yaiY	4	4	3	0	-1	1
yaiZ	4	4	4	0	0	0
yajC	1	1	1	0	0	0
yajD	4	4	3	0	-1	1
yajG	4	4	4	0	0	0
yajI	4	3	3	-1	-1	0
yajL	1	1	1	0	0	0
yajO	1	3	2	2	1	1
yajQ	2	2	1	0	-1	1
yajR	4	4	4	0	0	0
ybaA	4	4	4	0	0	0
ybaB	1	1	1	0	0	0
ybaE	4	4	4	0	0	0
ybaK	1	1	1	0	0	0
ybaL	4	4	3	0	-1	1
ybaM	4	3	2	-1	-2	1
ybaN	4	4	4	0	0	0
ybaP	4	4	4	0	0	0
ybaQ	4	4	3	0	-1	1
ybaT	4	4	3	0	-1	1
ybaV	2	2	1	0	-1	1
ybaY	1	1	1	0	0	0
ybbA	3	3	2	0	-1	1
ybbC	4	3	3	-1	-1	0
ybbJ	4	3	3	-1	-1	0
ybbO	3	3	2	0	-1	1
ybbP	2	3	2	1	0	1
ybbW	2	3	1	1	-1	2
ybbY	3	3	3	0	0	0
ybcF	3	3	3	0	0	0
ybcH	2	4	3	2	1	1

ybcI	4	4	4	0	0	0
ybcJ	2	3	2	1	0	1
ybdD	4	4	2	0	-2	2
ybdF	4	4	3	0	-1	1
ybdG	1	1	1	0	0	0
ybdJ	4	4	3	0	-1	1
ybdK	2	3	2	1	0	1
ybdL	4	4	3	0	-1	1
ybdM	2	3	2	1	0	1
ybdN	3	4	3	1	0	1
ybdO	4	4	4	0	0	0
ybdR	4	4	3	0	-1	1
ybdZ	3	3	3	0	0	0
ybeD	1	1	1	0	0	0
ybeF	4	4	4	0	0	0
ybeL	2	3	2	1	0	1
ybeQ	4	4	3	0	-1	1
ybeR	4	4	3	0	-1	1
ybeT	4	4	3	0	-1	1
ybeU	4	4	3	0	-1	1
ybeX	3	2	2	-1	-1	0
ybeY	3	3	2	0	-1	1
ybeZ	3	3	2	0	-1	1
ybfA	4	4	3	0	-1	1
ybfB	4	4	3	0	-1	1
ybfC	1	1	1	0	0	0
ybfD	3	1	1	-2	-2	0
ybfE	4	4	4	0	0	0
ybfF	3	4	3	1	0	1
ybfP	4	3	2	-1	-2	1
ybfQ	1	1	1	0	0	0
ybgA	3	2	2	-1	-1	0
ybgC	4	4	4	0	0	0
ybgE	1	1	1	0	0	0
ybgI	3	3	1	0	-2	2
ybgO	3	3	3	0	0	0
ybgP	3	3	3	0	0	0
ybgQ	4	4	3	0	-1	1
ybgS	1	1	1	0	0	0
ybgU	4	4	4	0	0	0
ybhA	4	4	4	0	0	0
ybhB	1	1	1	0	0	0
ybhC	2	3	2	1	0	1
ybhD	4	3	3	-1	-1	0
ybhF	4	4	3	0	-1	1
ybhG	4	4	3	0	-1	1
ybhH	4	3	2	-1	-2	1
ybhI	4	3	2	-1	-2	1
ybhJ	3	4	3	1	0	1
ybhK	4	3	3	-1	-1	0
ybhL	2	2	2	0	0	0

ybhM	4	4	3	0	-1	1
ybhN	4	4	4	0	0	0
ybhP	4	4	3	0	-1	1
ybhQ	4	4	3	0	-1	1
ybhR	4	4	3	0	-1	1
ybhS	3	4	3	1	0	1
ybiA	4	4	3	0	-1	1
ybiB	1	1	1	0	0	0
ybiI	4	4	4	0	0	0
ybiJ	4	4	3	0	-1	1
ybiO	4	4	3	0	-1	1
ybiR	4	4	4	0	0	0
ybiT	1	1	1	0	0	0
ybiU	1	2	2	1	1	0
ybiV	2	3	3	1	1	0
ybiW	2	3	2	1	0	1
ybiX	4	4	3	0	-1	1
ybiY	4	4	3	0	-1	1
ybjC	3	3	2	0	-1	1
ybjD	3	4	3	1	0	1
ybjG	4	4	3	0	-1	1
ybjH	3	4	3	1	0	1
ybjI	2	3	2	1	0	1
ybjJ	4	4	3	0	-1	1
ybjL	4	4	4	0	0	0
ybjM	4	4	3	0	-1	1
ybjN	3	3	2	0	-1	1
ybjO	4	4	4	0	0	0
ybjP	1	1	1	0	0	0
ybjQ	4	3	2	-1	-2	1
ybjS	4	4	4	0	0	0
ybjT	4	3	3	-1	-1	0
ybjX	4	4	4	0	0	0
ycaC	1	1	1	0	0	0
ycaD	4	4	4	0	0	0
ycaI	4	4	3	0	-1	1
ycaK	1	1	1	0	0	0
ycaL	3	2	2	-1	-1	0
ycaM	3	2	2	-1	-1	0
ycaN	4	3	2	-1	-2	1
ycaO	1	1	1	0	0	0
ycaP	4	3	3	-1	-1	0
ycaQ	4	4	3	0	-1	1
ycaR	2	2	2	0	0	0
ycbF	4	4	3	0	-1	1
ycbJ	4	3	3	-1	-1	0
ycbK	3	4	3	1	0	1
ycbU	4	2	2	-2	-2	0
ycbV	4	4	3	0	-1	1
ycbX	3	4	3	1	0	1
ycbZ	4	4	4	0	0	0

yccA	3	3	3	0	0	0
yccE	3	1	1	-2	-2	0
yccF	4	3	3	-1	-1	0
yccJ	1	1	1	0	0	0
yccM	4	4	4	0	0	0
yccS	4	3	3	-1	-1	0
yccT	4	4	3	0	-1	1
yccU	1	1	1	0	0	0
yccX	3	3	2	0	-1	1
ycdU	3	4	3	1	0	1
ycdX	2	2	2	0	0	0
ycdY	1	1	1	0	0	0
ycdZ	4	4	4	0	0	0
yceA	3	4	3	1	0	1
yceB	2	3	2	1	0	1
yceD	2	1	1	-1	-1	0
yceF	3	4	3	1	0	1
yceH	2	2	1	0	-1	1
yceI	1	1	1	0	0	0
yceJ	1	1	1	0	0	0
yceK	1	1	1	0	0	0
yceM	4	3	1	-1	-3	2
yceO	4	4	4	0	0	0
yceQ	4	4	3	0	-1	1
ycfH	1	2	2	1	1	0
ycfJ	4	4	2	0	-2	2
ycfL	1	1	1	0	0	0
ycfP	1	1	1	0	0	0
ycfT	4	4	4	0	0	0
ycfZ	4	4	3	0	-1	1
ycgB	4	4	3	0	-1	1
ycgJ	1	1	1	0	0	0
ycgL	4	4	3	0	-1	1
ycgM	4	4	2	0	-2	2
ycgN	4	4	4	0	0	0
ycgR	3	3	3	0	0	0
ycgV	4	3	3	-1	-1	0
ycgX	4	4	4	0	0	0
ycgY	4	4	3	0	-1	1
ycgZ	4	4	3	0	-1	1
ychA	4	4	4	0	0	0
ychE	4	4	4	0	0	0
ychF	2	3	3	1	1	0
ychH	4	4	2	0	-2	2
ychJ	4	4	4	0	0	0
ychN	1	1	1	0	0	0
ychO	3	3	3	0	0	0
ychQ	4	4	4	0	0	0
ychS	4	2	2	-2	-2	0
yciA	4	4	3	0	-1	1
yciB	2	2	2	0	0	0

yciC	2	3	2	1	0	1
yciE	2	2	1	0	-1	1
yciF	1	1	1	0	0	0
yciH	1	3	3	2	2	0
yciI	2	2	1	0	-1	1
yciK	4	4	4	0	0	0
yciN	2	1	1	-1	-1	0
yciO	3	3	3	0	0	0
yciQ	4	4	4	0	0	0
yciT	4	4	3	0	-1	1
yciU	3	3	3	0	0	0
yciV	4	4	3	0	-1	1
yciW	4	4	4	0	0	0
yciX	4	4	4	0	0	0
yciY	3	1	1	-2	-2	0
yciZ	4	4	3	0	-1	1
ycjD	1	1	1	0	0	0
ycjF	1	2	1	1	0	1
ycjG	3	2	2	-1	-1	0
ycjM	4	4	4	0	0	0
ycjN	2	2	1	0	-1	1
ycjO	1	2	2	1	1	0
ycjP	1	2	1	1	0	1
ycjQ	1	1	1	0	0	0
ycjR	1	1	1	0	0	0
ycjS	1	1	1	0	0	0
ycjT	1	1	1	0	0	0
ycjU	1	1	2	0	1	-1
ycjW	4	4	4	0	0	0
ycjX	2	3	2	1	0	1
ycjY	3	2	2	-1	-1	0
ydbD	3	3	3	0	0	0
ydbH	4	4	4	0	0	0
ydbJ	2	2	1	0	-1	1
ydbK	1	2	2	1	1	0
ydbL	4	4	4	0	0	0
ydcA	2	2	2	0	0	0
ydcC	4	2	2	-2	-2	0
ydcD	4	4	3	0	-1	1
ydcF	3	4	3	1	0	1
ydcH	1	1	1	0	0	0
ydcI	4	4	4	0	0	0
ydcJ	4	4	3	0	-1	1
ydcK	4	4	3	0	-1	1
ydcL	2	2	2	0	0	0
ydcO	3	2	2	-1	-1	0
ydcR	4	4	4	0	0	0
ydcS	3	3	3	0	0	0
ydcT	3	4	3	1	0	1
ydcU	3	4	3	1	0	1
ydcV	1	1	1	0	0	0

ydcY	3	3	2	0	-1	1
ydcZ	4	4	4	0	0	0
yddA	4	4	4	0	0	0
yddB	2	3	2	1	0	1
yddE	2	3	3	1	1	0
yddG	3	3	3	0	0	0
yddH	3	3	2	0	-1	1
yddK	4	3	3	-1	-1	0
yddL	4	4	3	0	-1	1
yddM	3	3	2	0	-1	1
yddW	4	4	4	0	0	0
yddY	4	4	4	0	0	0
ydeA	4	4	4	0	0	0
ydeE	4	4	4	0	0	0
ydeI	3	3	2	0	-1	1
ydeJ	4	3	3	-1	-1	0
ydeM	1	1	1	0	0	0
ydeN	1	1	1	0	0	0
ydeO	3	3	2	0	-1	1
ydeP	3	3	2	0	-1	1
ydeQ	4	4	2	0	-2	2
ydeR	4	4	3	0	-1	1
ydeS	4	4	2	0	-2	2
ydeT	4	4	3	0	-1	1
ydfA	4	4	4	0	0	0
ydfB	4	4	4	0	0	0
ydfC	4	4	4	0	0	0
ydfD	3	4	3	1	0	1
ydfG	1	1	1	0	0	0
ydfH	4	4	4	0	0	0
ydfI	3	2	2	-1	-1	0
ydfJ	4	2	2	-2	-2	0
ydfK	4	4	4	0	0	0
ydfN	4	3	2	-1	-2	1
ydfO	4	3	2	-1	-2	1
ydfR	4	4	3	0	-1	1
ydfT	3	3	2	0	-1	1
ydfU	4	4	4	0	0	0
ydfV	4	4	4	0	0	0
ydfW	4	4	4	0	0	0
ydfX	4	4	3	0	-1	1
ydfZ	4	4	3	0	-1	1
ydgA	1	1	1	0	0	0
ydgC	4	4	3	0	-1	1
ydgD	4	4	3	0	-1	1
ydgH	1	2	2	1	1	0
ydgI	4	4	4	0	0	0
ydgJ	3	3	3	0	0	0
ydgK	4	4	3	0	-1	1
ydgU	4	3	2	-1	-2	1
ydhB	4	4	4	0	0	0

ydhC	2	3	2	1	0	1
ydhF	4	4	3	0	-1	1
ydhI	4	4	3	0	-1	1
ydhJ	4	3	3	-1	-1	0
ydhK	3	3	3	0	0	0
ydhL	4	4	3	0	-1	1
ydhP	4	4	4	0	0	0
ydhQ	1	1	1	0	0	0
ydhR	1	2	1	1	0	1
ydhS	4	4	3	0	-1	1
ydhT	3	3	3	0	0	0
ydhU	3	4	3	1	0	1
ydhV	1	2	2	1	1	0
ydhW	3	3	3	0	0	0
ydhX	3	3	3	0	0	0
ydhY	1	2	1	1	0	1
ydiB	1	1	1	0	0	0
ydiE	4	4	4	0	0	0
ydiF	1	1	1	0	0	0
ydiH	3	2	2	-1	-1	0
ydiJ	1	1	1	0	0	0
ydiK	3	2	2	-1	-1	0
ydiL	4	4	4	0	0	0
ydiM	4	4	4	0	0	0
ydiN	3	3	2	0	-1	1
ydiO	1	1	1	0	0	0
ydiP	2	2	2	0	0	0
ydiQ	3	3	3	0	0	0
ydiR	2	3	2	1	0	1
ydiS	1	2	2	1	1	0
ydiT	1	2	1	1	0	1
ydiU	4	4	4	0	0	0
ydiV	4	4	3	0	-1	1
ydiY	3	2	2	-1	-1	0
ydiZ	2	1	1	-1	-1	0
ydjA	3	2	1	-1	-2	1
ydjE	4	4	4	0	0	0
ydjF	4	4	4	0	0	0
ydjG	3	2	2	-1	-1	0
ydjH	1	1	1	0	0	0
ydjI	1	1	1	0	0	0
ydjJ	2	2	2	0	0	0
ydjK	4	4	3	0	-1	1
ydjL	4	4	4	0	0	0
ydjM	4	4	4	0	0	0
ydjO	4	4	4	0	0	0
ydjX	4	4	3	0	-1	1
ydjY	2	2	1	0	-1	1
ydjZ	3	3	2	0	-1	1
yeaC	1	1	1	0	0	0
yeaD	1	1	1	0	0	0

yeaE	3	3	3	0	0	0
yeaG	2	3	2	1	0	1
yeaH	3	3	2	0	-1	1
yeaK	3	2	2	-1	-1	0
yeaL	3	3	3	0	0	0
yeaO	2	2	1	0	-1	1
yeaQ	1	1	1	0	0	0
yeaR	3	3	2	0	-1	1
yeaV	4	4	3	0	-1	1
yeaW	1	1	1	0	0	0
yeaX	2	3	3	1	1	0
yeaY	3	4	3	1	0	1
yebB	3	3	3	0	0	0
yebC	1	1	1	0	0	0
yebE	2	2	2	0	0	0
yebF	1	2	3	1	2	-1
yebG	4	4	3	0	-1	1
yebK	4	4	4	0	0	0
yebO	4	3	3	-1	-1	0
yebQ	4	4	4	0	0	0
yebS	4	4	4	0	0	0
yebT	4	4	4	0	0	0
yebV	4	2	1	-2	-3	1
yebW	4	4	4	0	0	0
yebY	3	3	2	0	-1	1
yebZ	4	3	3	-1	-1	0
yecA	1	2	1	1	0	1
yecD	4	3	2	-1	-2	1
yecE	4	4	3	0	-1	1
yecF	1	1	1	0	0	0
yecH	3	4	3	1	0	1
yecJ	2	2	2	0	0	0
yecM	2	3	2	1	0	1
yecN	4	3	3	-1	-1	0
yecR	4	4	3	0	-1	1
yecT	4	4	3	0	-1	1
yecU	1	1	1	0	0	0
yedA	4	4	3	0	-1	1
yedD	1	1	1	0	0	0
yedE	1	1	1	0	0	0
yedF	1	1	1	0	0	0
yedI	3	2	1	-1	-2	1
yedJ	4	4	3	0	-1	1
yedK	4	4	4	0	0	0
yedL	4	4	3	0	-1	1
yedP	3	3	2	0	-1	1
yedR	4	3	2	-1	-2	1
yedX	1	2	1	1	0	1
yeeA	2	3	2	1	0	1
yeeD	4	4	4	0	0	0
yeeE	4	4	3	0	-1	1

yeeJ	4	4	3	0	-1	1
yeeN	2	2	2	0	0	0
yeeO	4	4	4	0	0	0
yeeS	4	3	2	-1	-2	1
yeeT	4	3	3	-1	-1	0
yeeW	4	3	2	-1	-2	1
yeeX	3	3	2	0	-1	1
yeeY	4	4	3	0	-1	1
yeeZ	3	4	3	1	0	1
yefM	4	4	3	0	-1	1
yegD	2	4	3	2	1	1
yegH	3	3	3	0	0	0
yegI	4	4	3	0	-1	1
yegJ	2	1	1	-1	-1	0
yegL	4	4	3	0	-1	1
yegP	1	1	1	0	0	0
yegQ	3	3	3	0	0	0
yegR	2	3	1	1	-1	2
yegS	2	3	2	1	0	1
yegT	2	2	2	0	0	0
yegU	1	1	1	0	0	0
yegV	2	2	2	0	0	0
yegW	4	4	4	0	0	0
yegX	4	4	4	0	0	0
yehA	4	4	4	0	0	0
yehB	4	4	3	0	-1	1
yehE	3	4	3	1	0	1
yehF	4	3	3	-1	-1	0
yehI	4	4	3	0	-1	1
yehK	4	3	3	-1	-1	0
yehL	4	4	3	0	-1	1
yehM	4	3	3	-1	-1	0
yehP	3	3	2	0	-1	1
yehQ	3	3	3	0	0	0
yehR	3	3	2	0	-1	1
yehS	4	4	3	0	-1	1
yehW	4	4	3	0	-1	1
yehX	4	4	3	0	-1	1
yehY	3	3	2	0	-1	1
yeiB	4	4	3	0	-1	1
yeiE	4	4	3	0	-1	1
yeiG	1	2	2	1	1	0
yeiH	2	2	1	0	-1	1
yeiI	4	3	2	-1	-2	1
yeiL	4	4	3	0	-1	1
yeiP	3	3	3	0	0	0
yeiQ	4	4	4	0	0	0
yeiR	3	4	3	1	0	1
yeiS	4	4	4	0	0	0
yeiW	4	4	3	0	-1	1
yejA	4	4	4	0	0	0

yejB	4	4	4	0	0	0
yejE	3	4	3	1	0	1
yejF	2	3	3	1	1	0
yejG	4	4	3	0	-1	1
yejK	2	2	2	0	0	0
yejL	4	4	3	0	-1	1
yejM	4	4	4	0	0	0
yfaA	4	3	3	-1	-1	0
yfaE	3	3	2	0	-1	1
yfaL	4	4	3	0	-1	1
yfaP	2	1	1	-1	-1	0
yfaQ	2	1	1	-1	-1	0
yfaT	4	4	4	0	0	0
yfaU	1	2	1	1	0	1
yfaV	1	1	1	0	0	0
yfaX	2	3	3	1	1	0
yfaY	1	1	1	0	0	0
yfaZ	1	1	1	0	0	0
yfbK	3	1	1	-2	-2	0
yfbL	3	1	1	-2	-2	0
yfbM	2	1	1	-1	-1	0
yfbN	1	1	1	0	0	0
yfbO	1	1	1	0	0	0
yfbP	1	1	1	0	0	0
yfbR	3	3	2	0	-1	1
yfbS	4	4	3	0	-1	1
yfbU	1	2	1	1	0	1
yfbV	4	3	2	-1	-2	1
yfcA	4	4	4	0	0	0
yfcC	4	3	3	-1	-1	0
yfcD	1	1	1	0	0	0
yfcE	1	1	1	0	0	0
yfcF	2	3	2	1	0	1
yfcG	3	3	3	0	0	0
yfcH	4	3	2	-1	-2	1
yfcJ	4	3	3	-1	-1	0
yfcL	1	1	1	0	0	0
yfcO	4	4	3	0	-1	1
yfcP	4	4	3	0	-1	1
yfcQ	4	3	3	-1	-1	0
yfcR	4	4	3	0	-1	1
yfcS	4	4	3	0	-1	1
yfcV	3	3	2	0	-1	1
yfcZ	1	1	1	0	0	0
yfdC	4	4	3	0	-1	1
yfdE	4	4	3	0	-1	1
yfdF	3	4	4	1	1	0
yfdV	3	2	1	-1	-2	1
yfdX	2	2	2	0	0	0
yfdY	4	4	3	0	-1	1
yfeC	4	4	4	0	0	0

yfeD	4	4	4	0	0	0
yfeH	4	4	3	0	-1	1
yfeK	4	4	3	0	-1	1
yfeN	4	4	3	0	-1	1
yfeO	4	4	3	0	-1	1
yfeR	4	4	3	0	-1	1
yfeS	4	4	3	0	-1	1
yfeW	4	4	3	0	-1	1
yfeX	1	1	1	0	0	0
yfeY	1	2	1	1	0	1
yfeZ	4	4	3	0	-1	1
yffB	3	3	2	0	-1	1
yfgD	1	1	1	0	0	0
yfgG	4	4	4	0	0	0
yfgH	4	2	2	-2	-2	0
yfgI	3	3	3	0	0	0
yfgJ	1	1	1	0	0	0
yfgM	1	2	1	1	0	1
yfgO	4	4	3	0	-1	1
yfhH	4	4	3	0	-1	1
yfhL	4	4	4	0	0	0
yfhM	3	3	3	0	0	0
yfhR	4	4	4	0	0	0
yfiB	4	4	3	0	-1	1
yfiC	4	4	4	0	0	0
yfiE	4	4	4	0	0	0
yfiF	1	2	1	1	0	1
yfiL	4	4	3	0	-1	1
yfiM	3	2	1	-1	-2	1
yfiP	4	4	4	0	0	0
yfiR	4	4	4	0	0	0
yfjD	4	4	4	0	0	0
yfjF	3	4	3	1	0	1
yfjI	4	4	4	0	0	0
yfjQ	4	2	2	-2	-2	0
yfjR	4	3	2	-1	-2	1
yfjS	3	2	2	-1	-1	0
ygaC	4	4	3	0	-1	1
ygaH	4	4	4	0	0	0
ygaM	1	1	1	0	0	0
ygaP	3	2	2	-1	-1	0
ygaV	3	3	2	0	-1	1
ygaZ	4	4	4	0	0	0
ygbA	4	4	3	0	-1	1
ygbE	4	4	4	0	0	0
ygbI	3	3	2	0	-1	1
ygbJ	2	3	2	1	0	1
ygbK	1	3	2	2	1	1
ygbL	1	2	1	1	0	1
ygbM	1	1	1	0	0	0
ygbN	1	3	3	2	2	0

ygcE	1	2	2	1	1	0
ygcG	3	2	2	-1	-1	0
ygcN	4	4	4	0	0	0
ygcO	4	4	3	0	-1	1
ygcP	3	4	3	1	0	1
ygcQ	4	4	3	0	-1	1
ygcR	3	3	2	0	-1	1
ygcS	3	4	3	1	0	1
ygcU	2	3	3	1	1	0
ygcW	2	2	2	0	0	0
ygdB	4	2	2	-2	-2	0
ygdD	4	4	3	0	-1	1
ygdG	4	4	3	0	-1	1
ygdI	2	4	3	2	1	1
ygdQ	4	4	4	0	0	0
ygdR	4	3	2	-1	-2	1
ygdT	4	4	4	0	0	0
ygeA	3	3	2	0	-1	1
ygeH	4	2	2	-2	-2	0
ygeR	4	4	4	0	0	0
ygeV	4	4	3	0	-1	1
ygeW	1	2	2	1	1	0
ygeX	2	3	2	1	0	1
ygeY	1	1	1	0	0	0
ygfB	4	4	4	0	0	0
ygfI	4	4	3	0	-1	1
ygfK	3	2	1	-1	-2	1
ygfM	1	2	2	1	1	0
ygfS	3	3	2	0	-1	1
ygfT	4	3	2	-1	-2	1
ygfX	4	3	3	-1	-1	0
ygfZ	3	4	3	1	0	1
yggC	4	4	4	0	0	0
yggE	1	1	1	0	0	0
yggF	3	2	2	-1	-1	0
yggI	4	4	4	0	0	0
yggL	1	1	1	0	0	0
yggM	4	4	3	0	-1	1
yggN	2	3	2	1	0	1
yggP	1	1	1	0	0	0
yggR	4	3	3	-1	-1	0
yggS	4	4	3	0	-1	1
yggT	3	3	2	0	-1	1
yggU	3	3	2	0	-1	1
yggW	3	3	2	0	-1	1
yggX	1	2	1	1	0	1
yghA	1	2	1	1	0	1
yghB	4	4	4	0	0	0
yghD	1	3	2	2	1	1
yghG	3	4	3	1	0	1
yghJ	4	3	3	-1	-1	0

yghO	4	4	4	0	0	0
yghQ	4	3	3	-1	-1	0
yghR	4	3	3	-1	-1	0
yghS	4	3	2	-1	-2	1
yghT	4	3	2	-1	-2	1
yghU	1	2	1	1	0	1
yghW	4	4	4	0	0	0
ygiA	3	3	3	0	0	0
ygiB	3	3	2	0	-1	1
ygiC	2	2	2	0	0	0
ygiD	4	4	3	0	-1	1
ygiF	4	4	3	0	-1	1
ygiL	1	1	1	0	0	0
ygiM	3	4	3	1	0	1
ygiN	1	2	1	1	0	1
ygiQ	2	3	3	1	1	0
ygiS	2	1	1	-1	-1	0
ygiV	4	2	2	-2	-2	0
ygiW	1	1	1	0	0	0
ygiZ	4	3	2	-1	-2	1
ygjH	2	2	2	0	0	0
ygjI	1	1	1	0	0	0
ygjJ	2	3	3	1	1	0
ygjK	2	3	3	1	1	0
ygjP	4	4	4	0	0	0
ygjQ	4	4	4	0	0	0
ygjR	4	4	4	0	0	0
ygjV	4	4	4	0	0	0
yhaB	4	4	3	0	-1	1
yhaC	4	3	2	-1	-2	1
yhaH	1	1	1	0	0	0
yhaI	4	4	4	0	0	0
yhaJ	4	4	3	0	-1	1
yhaK	4	4	3	0	-1	1
yhaL	3	2	1	-1	-2	1
yhaV	4	4	3	0	-1	1
yhbE	4	4	3	0	-1	1
yhbO	1	2	1	1	0	1
yhbP	3	3	2	0	-1	1
yhbQ	2	2	1	0	-1	1
yhbS	1	1	1	0	0	0
yhbT	2	1	1	-1	-1	0
yhbU	4	3	2	-1	-2	1
yhbV	4	3	3	-1	-1	0
yhbW	1	2	1	1	0	1
yhbX	4	4	4	0	0	0
yhbY	3	3	2	0	-1	1
yhcA	3	2	3	-1	0	-1
yhcB	2	2	1	0	-1	1
yhcC	4	4	3	0	-1	1
yhcD	4	4	4	0	0	0

yhcF	4	4	4	0	0	0
yhcG	4	4	3	0	-1	1
yhcH	1	1	1	0	0	0
yhcN	3	3	2	0	-1	1
yhcO	1	1	1	0	0	0
yhdE	4	4	4	0	0	0
yhdH	1	1	1	0	0	0
yhdJ	4	4	4	0	0	0
yhdN	1	1	1	0	0	0
yhdP	2	2	2	0	0	0
yhdT	2	3	3	1	1	0
yhdU	4	4	4	0	0	0
yhdV	4	4	3	0	-1	1
yhdX	3	3	3	0	0	0
yhdY	1	3	2	2	1	1
yhdZ	1	1	1	0	0	0
yheO	4	4	4	0	0	0
yheS	4	4	4	0	0	0
yheT	4	4	3	0	-1	1
yheU	4	3	2	-1	-2	1
yheV	1	1	1	0	0	0
yhfA	4	4	4	0	0	0
yhfG	4	4	3	0	-1	1
yhfK	4	4	4	0	0	0
yhfL	1	1	1	0	0	0
yhfS	1	1	1	0	0	0
yhfT	1	1	1	0	0	0
yhfU	1	1	1	0	0	0
yhfW	1	3	1	2	0	2
yhfX	3	4	2	1	-1	2
yhfY	4	4	3	0	-1	1
yhfZ	4	4	4	0	0	0
yhgE	4	4	4	0	0	0
yhgF	1	1	1	0	0	0
yhgH	2	1	1	-1	-1	0
yhgN	4	4	3	0	-1	1
yhhA	2	2	1	0	-1	1
yhhH	3	3	1	0	-2	2
yhhI	3	2	2	-1	-1	0
yhhJ	3	3	3	0	0	0
yhhL	4	4	3	0	-1	1
yhhM	4	4	3	0	-1	1
yhhN	4	4	3	0	-1	1
yhhQ	4	4	4	0	0	0
yhhS	4	4	4	0	0	0
yhhT	4	4	3	0	-1	1
yhhW	3	4	3	1	0	1
yhhX	3	3	3	0	0	0
yhhY	3	2	1	-1	-2	1
yhhZ	4	3	3	-1	-1	0
yhiD	4	4	3	0	-1	1

yhiI	4	4	3	0	-1	1
yhiJ	4	4	4	0	0	0
yhiM	3	4	3	1	0	1
yhiN	4	4	4	0	0	0
yhjB	3	3	3	0	0	0
yhjC	4	4	3	0	-1	1
yhjD	4	4	3	0	-1	1
yhjE	3	4	3	1	0	1
yhjG	3	3	2	0	-1	1
yhjJ	3	4	3	1	0	1
yhjR	4	4	4	0	0	0
yhjV	1	2	1	1	0	1
yhjX	4	4	3	0	-1	1
yhjY	4	4	3	0	-1	1
yiaA	4	4	4	0	0	0
yiaB	4	3	3	-1	-1	0
yiaC	2	3	2	1	0	1
yiaD	1	1	1	0	0	0
yiaF	1	1	1	0	0	0
yiaG	1	1	1	0	0	0
yiaJ	4	4	3	0	-1	1
yiaK	3	3	2	0	-1	1
yiaL	3	2	2	-1	-1	0
yiaM	4	4	3	0	-1	1
yiaN	3	4	3	1	0	1
yiaO	1	2	1	1	0	1
yiaT	4	3	3	-1	-1	0
yiaU	4	4	4	0	0	0
yiaV	4	4	3	0	-1	1
yiaY	1	1	1	0	0	0
yibA	3	3	2	0	-1	1
yibF	2	3	2	1	0	1
yibG	3	2	2	-1	-1	0
yibH	4	4	2	0	-2	2
yibI	4	4	3	0	-1	1
yibL	4	4	4	0	0	0
yibN	3	3	2	0	-1	1
yibQ	4	4	4	0	0	0
yibT	3	4	2	1	-1	2
yicC	3	3	3	0	0	0
yicG	4	4	4	0	0	0
yicH	4	4	3	0	-1	1
yicI	3	3	2	0	-1	1
yicJ	3	3	3	0	0	0
yicL	4	4	3	0	-1	1
yicN	4	4	3	0	-1	1
yicR	4	3	2	-1	-2	1
yicS	4	4	4	0	0	0
yidA	3	3	3	0	0	0
yidB	3	3	3	0	0	0
yidC	3	4	3	1	0	1

yidD	4	4	4	0	0	0
yidE	4	4	4	0	0	0
yidF	4	4	4	0	0	0
yidG	4	4	3	0	-1	1
yidH	4	4	3	0	-1	1
yidI	4	4	3	0	-1	1
yidL	4	4	4	0	0	0
yidP	4	4	4	0	0	0
yidQ	4	4	4	0	0	0
yidR	4	4	4	0	0	0
yidX	4	3	3	-1	-1	0
yidZ	4	4	4	0	0	0
yieE	3	3	3	0	0	0
yieF	1	1	1	0	0	0
yieH	3	3	3	0	0	0
yieK	3	3	2	0	-1	1
yieL	2	2	1	0	-1	1
yieP	4	3	3	-1	-1	0
yifB	4	4	3	0	-1	1
yifK	1	1	1	0	0	0
yifL	4	4	4	0	0	0
yigA	4	4	3	0	-1	1
yigB	3	4	3	1	0	1
yigE	3	4	3	1	0	1
yigF	4	4	3	0	-1	1
yigG	4	4	3	0	-1	1
yigI	4	4	3	0	-1	1
yigL	4	4	3	0	-1	1
yigZ	2	2	2	0	0	0
yihA	3	4	3	1	0	1
yihD	1	1	1	0	0	0
yihF	3	4	3	1	0	1
yihG	4	4	4	0	0	0
yihI	3	4	3	1	0	1
yihL	4	4	4	0	0	0
yihM	4	4	4	0	0	0
yihN	1	2	1	1	0	1
yihO	3	3	2	0	-1	1
yihP	2	2	2	0	0	0
yihQ	1	2	2	1	1	0
yihR	2	3	3	1	1	0
yihS	1	2	2	1	1	0
yihT	1	2	1	1	0	1
yihU	1	3	2	2	1	1
yihV	3	3	3	0	0	0
yihW	4	4	3	0	-1	1
yihX	4	4	3	0	-1	1
yihY	4	4	3	0	-1	1
yiiD	3	3	2	0	-1	1
yiiE	4	4	4	0	0	0
yiiF	4	4	3	0	-1	1

yiiG	4	3	3	-1	-1	0
yiiM	1	2	2	1	1	0
yiiQ	4	4	4	0	0	0
yiiR	4	4	3	0	-1	1
yiiS	1	1	1	0	0	0
yiiX	4	4	4	0	0	0
yijD	1	1	1	0	0	0
yijE	4	4	4	0	0	0
yijF	4	4	3	0	-1	1
yijO	4	4	4	0	0	0
yjaB	2	2	2	0	0	0
yjaG	3	4	3	1	0	1
yjaH	4	3	3	-1	-1	0
yjbB	4	4	3	0	-1	1
yjbD	3	3	3	0	0	0
yjbE	1	1	1	0	0	0
yjbF	4	2	2	-2	-2	0
yjbG	4	2	1	-2	-3	1
yjbH	4	3	3	-1	-1	0
yjbJ	1	1	1	0	0	0
yjbM	3	1	1	-2	-2	0
yjbQ	4	4	3	0	-1	1
yjbR	3	3	2	0	-1	1
yjbT	3	3	3	0	0	0
yjcB	4	4	3	0	-1	1
yjcE	1	1	1	0	0	0
yjcF	3	2	2	-1	-1	0
yjcH	1	1	1	0	0	0
yjcO	4	4	4	0	0	0
yjcS	3	4	4	1	1	0
yjcZ	4	4	3	0	-1	1
yjdC	1	2	1	1	0	1
yjdF	4	4	3	0	-1	1
yjdI	1	1	1	0	0	0
yjdJ	1	1	1	0	0	0
yjdM	1	1	1	0	0	0
yjdN	1	2	2	1	1	0
yjdP	4	3	3	-1	-1	0
yjeH	4	4	3	0	-1	1
yjeI	2	1	1	-1	-1	0
yjeJ	4	4	3	0	-1	1
yjeM	4	4	3	0	-1	1
yjeN	4	4	3	0	-1	1
yjeO	4	4	4	0	0	0
yjeT	1	1	1	0	0	0
yjeV	4	4	4	0	0	0
yjfC	4	3	3	-1	-1	0
yjfF	2	3	3	1	1	0
yjfI	4	4	3	0	-1	1
yjfJ	4	4	3	0	-1	1
yjfK	4	4	3	0	-1	1

yjfL	4	4	3	0	-1	1
yjfM	4	3	3	-1	-1	0
yjfN	4	4	2	0	-2	2
yjfP	4	4	3	0	-1	1
yjfY	4	3	3	-1	-1	0
yjgA	3	3	2	0	-1	1
yjgH	2	3	3	1	1	0
yjgL	3	3	2	0	-1	1
yjgM	4	4	3	0	-1	1
yjgN	4	3	3	-1	-1	0
yjgR	1	3	2	2	1	1
yjiA	2	3	2	1	0	1
yjiC	4	4	3	0	-1	1
yjiG	1	1	1	0	0	0
yjiH	3	1	1	-2	-2	0
yjiJ	4	3	3	-1	-1	0
yjiK	4	4	4	0	0	0
yjiL	4	4	3	0	-1	1
yjiM	3	3	2	0	-1	1
yjiN	4	4	4	0	0	0
yjiR	4	4	3	0	-1	1
yjiS	4	4	4	0	0	0
yjiX	3	3	2	0	-1	1
yjjA	4	4	4	0	0	0
yjjB	4	4	3	0	-1	1
yjjG	3	4	4	1	1	0
yjjI	3	3	3	0	0	0
yjjP	4	4	3	0	-1	1
yjjQ	4	4	3	0	-1	1
yjjU	3	3	2	0	-1	1
yjjV	3	3	2	0	-1	1
yjjW	3	3	3	0	0	0
yjjX	3	3	3	0	0	0
yjjY	3	3	3	0	0	0
yjjZ	4	4	4	0	0	0
yjtD	3	3	3	0	0	0
ykfA	4	3	3	-1	-1	0
ykfB	2	2	1	0	-1	1
ykfC	1	1	1	0	0	0
ykfF	4	4	3	0	-1	1
ykfG	4	3	2	-1	-2	1
ykfH	3	3	3	0	0	0
ykfI	4	2	2	-2	-2	0
ykfM	3	3	3	0	0	0
ykgE	2	2	1	0	-1	1
ykgF	1	1	1	0	0	0
ykgG	1	1	1	0	0	0
ykgH	4	4	3	0	-1	1
ykgJ	3	3	2	0	-1	1
ykgL	4	4	4	0	0	0
ykgM	1	1	1	0	0	0

ykgO	2	2	2	0	0	0
ykgR	4	4	3	0	-1	1
ykgS	4	4	4	0	0	0
ykiD	4	4	3	0	-1	1
ylaC	4	4	4	0	0	0
ylbE	1	2	2	1	1	0
ylbF	2	2	1	0	-1	1
ylbG	4	4	4	0	0	0
yldA	4	4	3	0	-1	1
yliI	2	3	3	1	1	0
yliM	4	3	2	-1	-2	1
ymcE	3	4	3	1	0	1
ymcF	4	4	4	0	0	0
ymdA	4	4	3	0	-1	1
ymdB	4	4	3	0	-1	1
ymdF	1	1	1	0	0	0
ymdG	4	4	2	0	-2	2
ymfA	4	3	3	-1	-1	0
ymgA	3	4	2	1	-1	2
ymgC	3	2	1	-1	-2	1
ymgD	1	1	1	0	0	0
ymgE	2	4	3	2	1	1
ymgF	4	4	4	0	0	0
ymgG	1	1	1	0	0	0
ymgI	1	1	1	0	0	0
ymgJ	4	4	4	0	0	0
ymgL	4	3	3	-1	-1	0
ymgM	4	4	3	0	-1	1
ymiA	4	4	4	0	0	0
ymiB	4	4	3	0	-1	1
ymiC	4	4	4	0	0	0
ymjA	4	4	4	0	0	0
ymjC	3	2	2	-1	-1	0
ymjD	3	2	2	-1	-1	0
ymjE	4	4	4	0	0	0
ynaE	4	3	4	-1	0	-1
ynaI	4	4	4	0	0	0
ynaJ	4	4	3	0	-1	1
ynaL	3	3	2	0	-1	1
ynbA	3	3	3	0	0	0
ynbB	2	3	2	1	0	1
ynbC	4	4	3	0	-1	1
ynbD	4	4	3	0	-1	1
ynbE	4	4	4	0	0	0
yncD	4	4	4	0	0	0
yncE	1	2	1	1	0	1
yncG	4	4	4	0	0	0
yncJ	4	4	4	0	0	0
yncL	2	3	3	1	1	0
yncO	4	4	3	0	-1	1
yneE	4	4	3	0	-1	1

yneG	4	4	3	0	-1	1
yneJ	4	4	4	0	0	0
yneK	4	4	3	0	-1	1
yneP	1	2	3	1	2	-1
ynfA	4	4	4	0	0	0
ynfB	4	3	2	-1	-2	1
ynfC	4	4	3	0	-1	1
ynfD	3	3	2	0	-1	1
ynfE	4	4	3	0	-1	1
ynfF	1	1	1	0	0	0
ynfG	1	1	1	0	0	0
ynfH	3	3	2	0	-1	1
ynfK	4	4	4	0	0	0
ynfL	4	4	3	0	-1	1
ynfM	4	4	3	0	-1	1
ynfN	4	3	1	-1	-3	2
ynfQ	4	4	2	0	-2	2
ynfR	4	4	2	0	-2	2
ynfS	4	4	4	0	0	0
ynfT	4	4	4	0	0	0
ynhF	2	3	3	1	1	0
yniA	3	2	2	-1	-1	0
yniB	4	3	2	-1	-2	1
yniD	4	4	4	0	0	0
ynjA	3	3	3	0	0	0
ynjB	2	3	2	1	0	1
ynjC	3	2	2	-1	-1	0
ynjD	3	2	2	-1	-1	0
ynjE	1	1	1	0	0	0
ynjF	4	4	3	0	-1	1
ynjH	4	4	3	0	-1	1
ynjI	4	4	4	0	0	0
yoaA	4	4	4	0	0	0
yoaB	1	1	1	0	0	0
yoaC	1	2	1	1	0	1
yoaE	3	2	2	-1	-1	0
yoaF	2	2	2	0	0	0
yoaG	3	3	1	0	-2	2
yoaH	4	4	3	0	-1	1
yoaI	3	2	2	-1	-1	0
yoaJ	4	4	4	0	0	0
yoaK	4	4	4	0	0	0
yoaL	3	3	3	0	0	0
yobA	4	4	3	0	-1	1
yobB	4	3	3	-1	-1	0
yobD	1	1	1	0	0	0
yobF	4	4	4	0	0	0
yobH	3	3	3	0	0	0
yodC	2	3	2	1	0	1
yodD	2	2	1	0	-1	1
yoeB	4	4	2	0	-2	2

yoeF	4	4	4	0	0	0
yoeI	1	3	3	2	2	0
yohC	4	3	2	-1	-2	1
yohD	4	4	3	0	-1	1
yohF	3	4	3	1	0	1
yohJ	4	4	3	0	-1	1
yohK	4	4	3	0	-1	1
yohO	4	4	4	0	0	0
yohP	2	2	2	0	0	0
yojI	4	4	4	0	0	0
ypdA	4	4	4	0	0	0
ypdB	4	4	4	0	0	0
ypdC	4	4	3	0	-1	1
ypdE	3	2	2	-1	-1	0
ypdF	3	1	1	-2	-2	0
ypdI	4	4	4	0	0	0
ypdK	4	4	3	0	-1	1
ypeA	4	4	3	0	-1	1
ypeB	3	3	2	0	-1	1
ypeC	4	3	3	-1	-1	0
ypfG	1	2	3	1	2	-1
ypfH	3	4	3	1	0	1
ypfJ	4	4	3	0	-1	1
ypfM	3	3	1	0	-2	2
ypfN	4	3	2	-1	-2	1
yphA	2	1	1	-1	-1	0
yphB	1	2	1	1	0	1
yphC	1	1	1	0	0	0
yphD	1	2	1	1	0	1
yphE	2	3	2	1	0	1
yphF	1	1	1	0	0	0
yphG	3	3	3	0	0	0
yphH	4	4	3	0	-1	1
ypjA	4	4	3	0	-1	1
ypjB	3	2	2	-1	-1	0
ypjD	4	4	4	0	0	0
ypjK	4	2	2	-2	-2	0
yqaA	4	4	3	0	-1	1
yqaB	4	4	4	0	0	0
yqaE	3	2	1	-1	-2	1
yqcA	2	3	2	1	0	1
yqcC	4	4	4	0	0	0
yqcE	3	4	4	1	1	0
yqeA	2	2	2	0	0	0
yqeB	3	4	2	1	-1	2
yqeC	4	4	3	0	-1	1
yqeF	4	4	4	0	0	0
yqeG	4	4	4	0	0	0
yqeH	4	4	3	0	-1	1
yqeI	4	4	4	0	0	0
yqeJ	4	4	4	0	0	0

yqeK	4	1	2	-3	-2	-1
yqeL	4	3	4	-1	0	-1
yqfA	4	3	2	-1	-2	1
yqfB	3	3	3	0	0	0
yqfI	3	3	3	0	0	0
yqgA	3	4	3	1	0	1
yqgB	1	2	2	1	1	0
yqgC	2	3	3	1	1	0
yqgD	3	4	3	1	0	1
yqgE	4	3	3	-1	-1	0
yqgF	4	3	3	-1	-1	0
yqhA	1	2	2	1	1	0
yqhC	4	4	4	0	0	0
yqhD	1	2	1	1	0	1
yqhG	4	3	3	-1	-1	0
yqhH	2	3	3	1	1	0
yqhI	4	4	3	0	-1	1
yqiA	4	4	4	0	0	0
yqiB	4	4	3	0	-1	1
yqiD	4	3	3	-1	-1	0
yqiH	4	4	3	0	-1	1
yqiI	4	4	4	0	0	0
yqiJ	4	4	4	0	0	0
yqiK	4	4	3	0	-1	1
yqjA	4	4	4	0	0	0
yqjC	2	1	1	-1	-1	0
yqjD	1	1	1	0	0	0
yqjE	1	1	1	0	0	0
yqjF	4	3	2	-1	-2	1
yqjG	2	1	1	-1	-1	0
yqjK	1	1	1	0	0	0
yraH	3	3	3	0	0	0
yraI	4	4	3	0	-1	1
yraJ	4	3	3	-1	-1	0
yraK	4	4	4	0	0	0
yraN	4	4	4	0	0	0
yraP	1	1	1	0	0	0
yraQ	3	2	2	-1	-1	0
yraR	4	4	3	0	-1	1
yrbG	4	4	4	0	0	0
yrbL	4	3	2	-1	-2	1
yrbN	2	3	3	1	1	0
yrdA	3	3	3	0	0	0
yrdB	3	3	2	0	-1	1
yrdD	4	4	3	0	-1	1
yrfG	3	3	2	0	-1	1
yrhB	4	4	3	0	-1	1
ysaA	4	3	3	-1	-1	0
ysaB	4	4	4	0	0	0
ysdD	4	4	3	0	-1	1
ysgA	1	2	2	1	1	0

yshB	1	2	2	1	1	0
ytfB	4	4	3	0	-1	1
ytfE	4	4	3	0	-1	1
ytfF	4	4	3	0	-1	1
ytfH	4	4	3	0	-1	1
ytfJ	4	3	3	-1	-1	0
ytfK	2	1	1	-1	-1	0
ytfL	4	4	4	0	0	0
ytfP	3	3	2	0	-1	1
ytfQ	2	3	2	1	0	1
ytfR	4	3	2	-1	-2	1
ytfT	3	2	1	-1	-2	1
ytiA	4	4	3	0	-1	1
ytiC	4	3	3	-1	-1	0
ytiD	4	3	3	-1	-1	0
ytjA	1	1	1	0	0	0
ytjB	4	4	3	0	-1	1
ytjC	2	3	2	1	0	1
yzcX	3	3	3	0	0	0
yzfA	3	3	2	0	-1	1
yzgL	2	1	2	-1	0	-1
zapA	4	4	3	0	-1	1
zapB	2	2	1	0	-1	1
zapC	4	4	4	0	0	0
zapD	4	4	4	0	0	0
zapE	4	3	3	-1	-1	0
zinT	1	1	1	0	0	0
zipA	4	4	4	0	0	0
zitB	4	3	3	-1	-1	0
zntA	4	4	3	0	-1	1
zntB	3	4	3	1	0	1
zntR	1	1	1	0	0	0
znuA	3	3	3	0	0	0
znuB	4	4	4	0	0	0
znuC	4	4	4	0	0	0
zraP	3	4	3	1	0	1
zraR	4	4	3	0	-1	1
zraS	4	4	4	0	0	0
zupT	4	4	2	0	-2	2
zur	4	4	4	0	0	0
zwf	1	1	1	0	0	0

[a] Only genes with more than 10 reads in at least one strain were considered

[b]Decreasing RNA stability from Cat. 1 to Cat. 4

[c]Negative values, genes destabilized in presence of EcPNPase; positive values, genes stabilized in presence of EcPNPase; 0, no difference in presence or absence of EcPNPase

[d]Negative values, genes more stable in presence of hPNPase than with EcPNPase; positive values, genes less stable in presence of hPNPase than with EcPNPase; 0, no difference in the two strains

[e]Negative values, genes destabilized in presence of hPNPase; positive values, genes stabilized in presence of hPNPase; 0, no difference in presence or absence of hPNPase
